# Supplementary material for: Enhancer recruitment of transcription repressors RUNX1 and TLE3 by mis-expressed FOXC1 blocks differentiation in acute myeloid leukemia
Source: Cell Rep. 2021 Sep 21;36(12):109725. doi: 10.1016/j.celrep.2021.109725 (PMC8480281; doi:10.1016/j.celrep.2021.109725)
Supplement: Document S1. Figures S1–S7 and Table S1 [file mmc1.pdf]

Cell Reports, Volume *36*

Supplemental information

Enhancer recruitment of transcription repressors RUNX1 and TLE3 by mis-expressed FOXC1 blocks differentiation in acute myeloid leukemia

Fabrizio Simeoni, Isabel Romero-Camarero, Francesco Camera, Fabio M.R. Amaral, Oliver J. Sinclair, Evangelia K. Papachristou, Gary J. Spencer, Michael Lie-A-Ling, Georges Lacaud, Daniel H. Wiseman, Jason S. Carroll, and Tim C.P. Somervaille

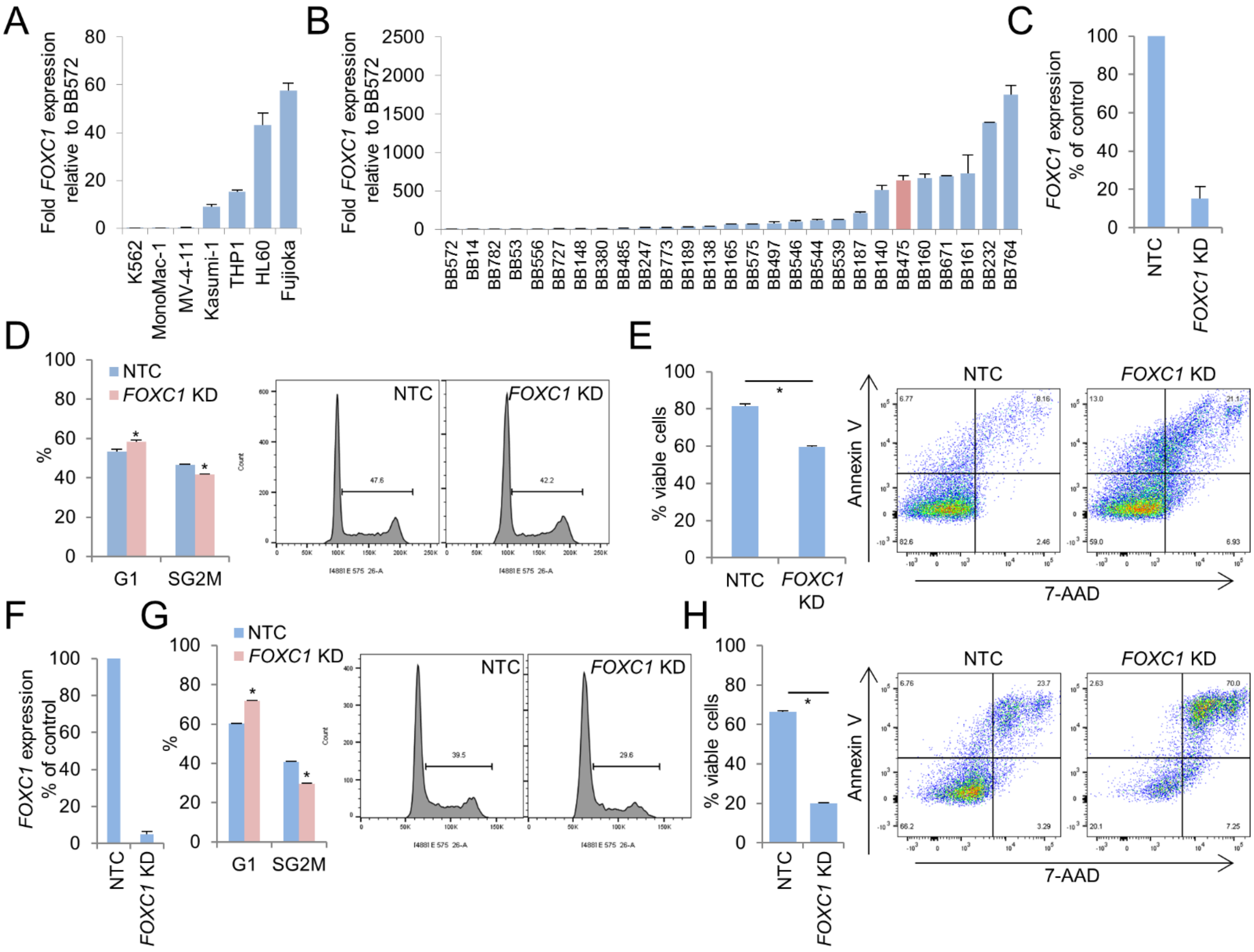

Figure S1. *FOXC1* expression in AML cells. Related to Figure 1.

Bar charts shows mean+SEM relative expression of *FOXC1* in (A) the indicated human AML cell lines and (B) bulk primary human AML samples (n=28), by Q-PCR. (C-E) Human Fujioka AML cells were infected with a lentivirus targeting *FOXC1* for KD or a non-targeting control vector (NTC). (C) Bar chart shows mean+SEM relative expression of *FOXC1* in KD versus control cells (n=3) 72hrs after initiation of KD. (D) Bar chart (left panel) shows mean+SEM percentage of cells in G1 or SG_2M six days following initiation of KD. Right panels: representative cell cycle profiles. (E) Bar chart (left panel) shows mean+SEM percentage of viable cells as determined by Annexin-V/7-AAD analysis seven days following initiation of KD (n=3). Right panels: representative flow cytometry plots. (F-H) Primary patient AML cells (BB475) were infected with a lentivirus targeting *FOXC1* for KD or NTC (n=2). (F) Bar chart shows mean+SEM relative expression of *FOXC1* KD in KD versus control cells (n=2) 72hrs after initiation of KD. (G) Bar chart (left panel) shows mean+SEM percentage of cells in G1 or SG_2M six days following initiation of KD. Right panels: representative cell cycle profiles (n=2). (H) Bar chart (left panel) shows mean+SEM percentage of viable cells as determined by Annexin-V/7-AAD analysis seven days following initiation of KD (n=2). * indicates $P<0.05$ for the indicated comparisons by t-test.

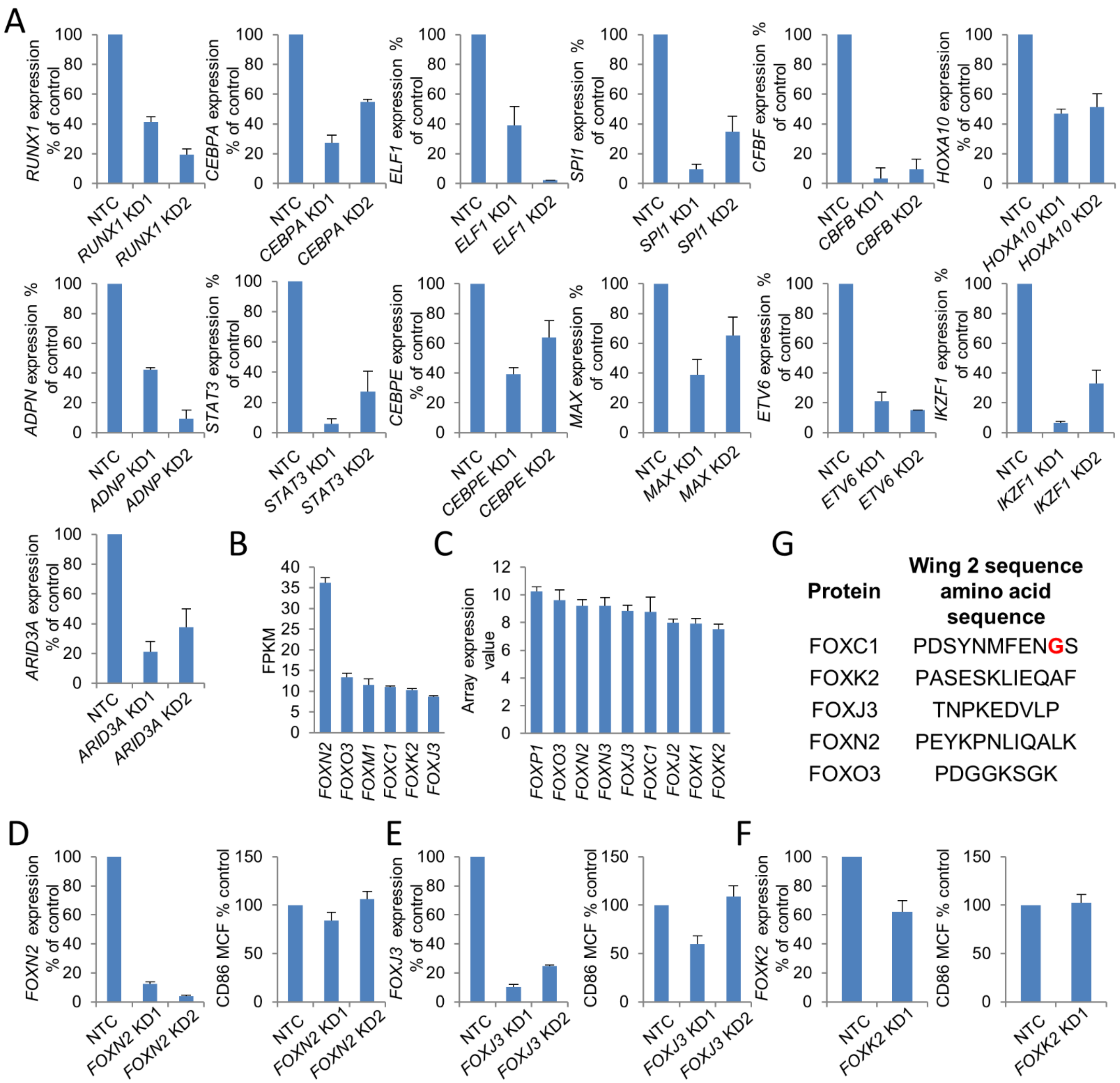

Figure S2. Knockdown of transcription factor genes in Fujioka AML cells. Related to Figure 2.

(A) Graphs show mean+SEM (n=3) relative expression following KD of the indicated genes in human Fujioka AML cells with the indicated lentiviral KD constructs relative to a non-targeting control (NTC). Mean+SEM expression values for all Forkhead family genes where expression values were among the top 33% of all protein coding genes in (B) Fujioka cells (fragments per kilobase per million mapped reads) (n=2) or (C) primary human FOXC1^{high} AML cases (n=100); data extracted from Wouters et al. (2009). (D-F) Graphs (left panels) show mean+SEM (n=3) relative expression following KD of the indicated genes in human Fujioka AML cells with the indicated lentiviral KD constructs relative to a non-targeting control (NTC) and (right panels) mean+SEM CD86 cell fluorescence as determined by flow cytometry analysis on Day 5 (n=3). (G) Amino acid sequences of Wing 2 of the Forkhead domains in the indicated human proteins. G165 of FOXC1 is highlighted in red.

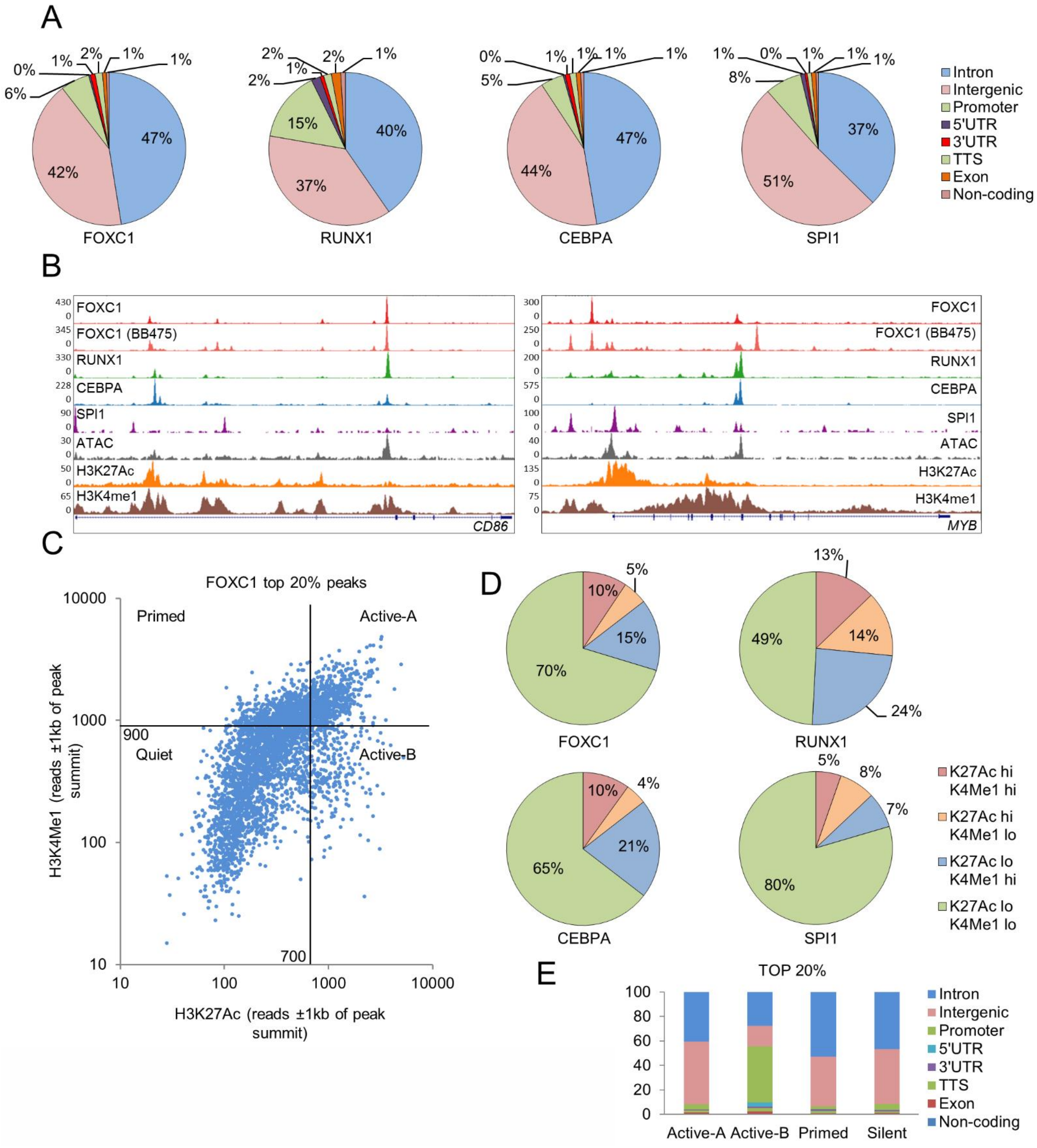

Figure S3. Chromatin context of FOXC1, RUNX1, CEBPA and SPI1 binding peaks. Related to Figure 3.

(A) Pie charts show genome annotations for all transcription factor binding peaks. (B) Exemplar ChIP-seq tracks. (C) Dot plot shows H3K27Ac versus H3K4Me1 reads ±1kB from the absolute summit of each of the strongest 20% of FOXC1 peaks (n=3,773). This facilitated the annotation of transcription factor binding peaks according to their surrounding chromatin into four categories. (D) Pie charts show chromatin categories for all transcription factor binding peaks. (E) Genome region annotations for the strongest 20% of FOXC1 peaks (n=3,773) according to chromatin category shown in (C).

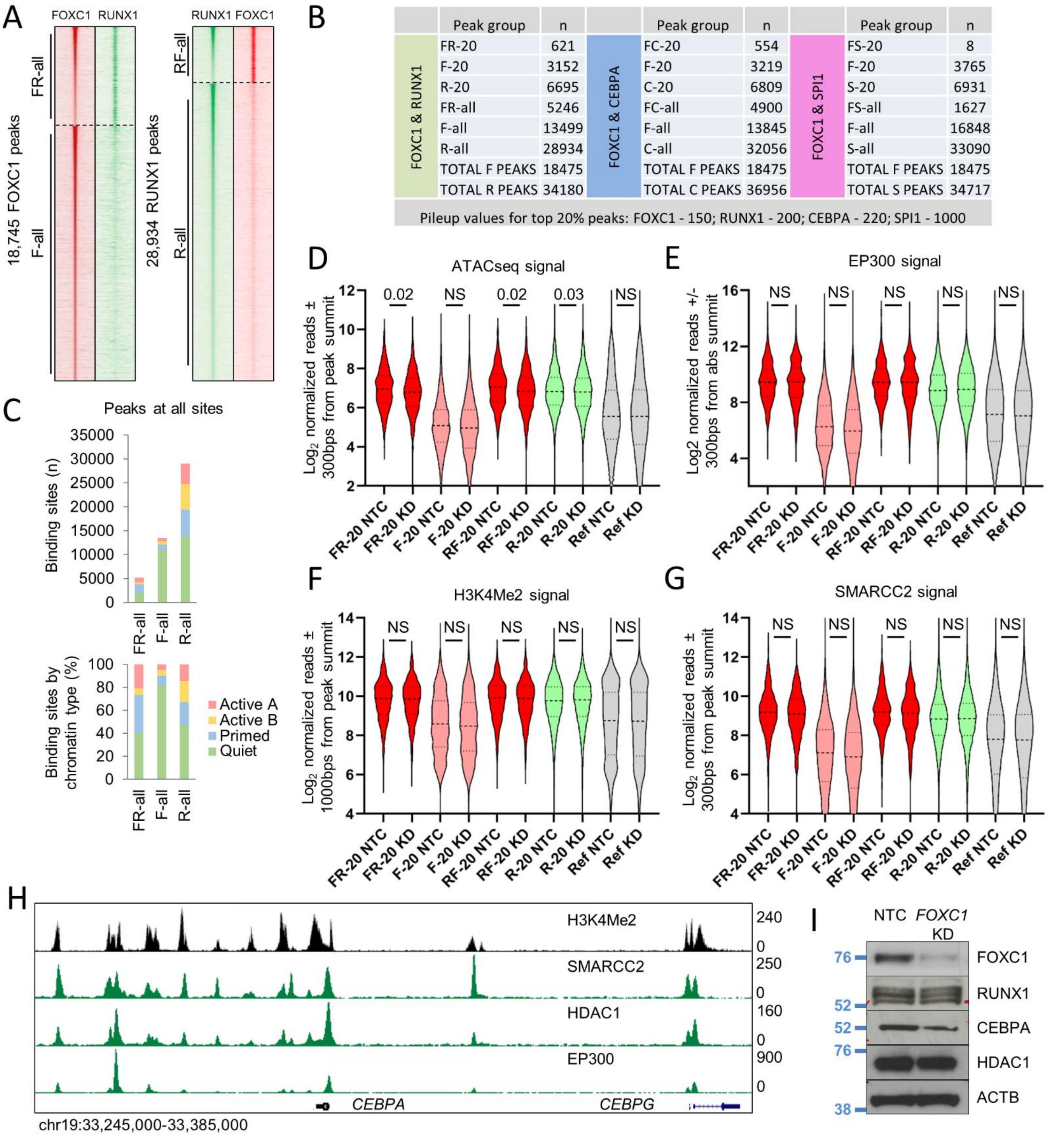

	Peak group	n		Peak group	n		Peak group	n
FOXC1 & RUNX1	FR-20	621	FOXC1 & CEBPA	FC-20	554	FOXC1 & SPI1	FS-20	8
	F-20	3152		F-20	3219		F-20	3765
	R-20	6695		C-20	6809		S-20	6931
	FR-all	5246		FC-all	4900		FS-all	1627
	F-all	13499		F-all	13845		F-all	16848
	R-all	28934		C-all	32056		S-all	33090
	TOTAL F PEAKS	18475		TOTAL F PEAKS	18475		TOTAL F PEAKS	18475
	TOTAL R PEAKS	34180		TOTAL C PEAKS	36956		TOTAL S PEAKS	34717

Pileup values for top 20% peaks: FOXC1 - 150; RUNX1 - 200; CEBPA - 220; SPI1 - 1000

Figure S4. FOXC1 colocalization with transcription and epigenetic factors. Related to Figure 4.

(A) Heatmaps show ChIP signal for RUNX1 at all FOXC1 binding sites (left panel) and FOXC1 at all RUNX1 binding sites (right panel). (B) Table shows the number of binding peaks in each of the indicated categories. (C) Bar charts show chromatin categories for the indicated classes of FOXC1 and RUNX1 binding peaks in Fujioka cells by number (upper panel) and proportion (lower panel). (D-G) Violin plots show distribution, median (thick dotted line) and interquartile range (light dotted lines) for ChIP signal for the indicated proteins at sites with strong FOXC1 and RUNX1 binding (FR-20, FOXC1 centered; RF-20 RUNX1 centered), FOXC1 binding (F-20) or RUNX1 binding (R-20) in Fujioka AML cells in control cells (NTC) or following *FOXC1* KD. Ref, reference cohort used for normalization between experiments; NS, not significant. *P* values, unpaired t-test. (H) Exemplar ChIP-seq tracks. (I) Western blots for the indicated proteins in Fujioka cells on Day 4 following initiation of FOXC1 knockdown.

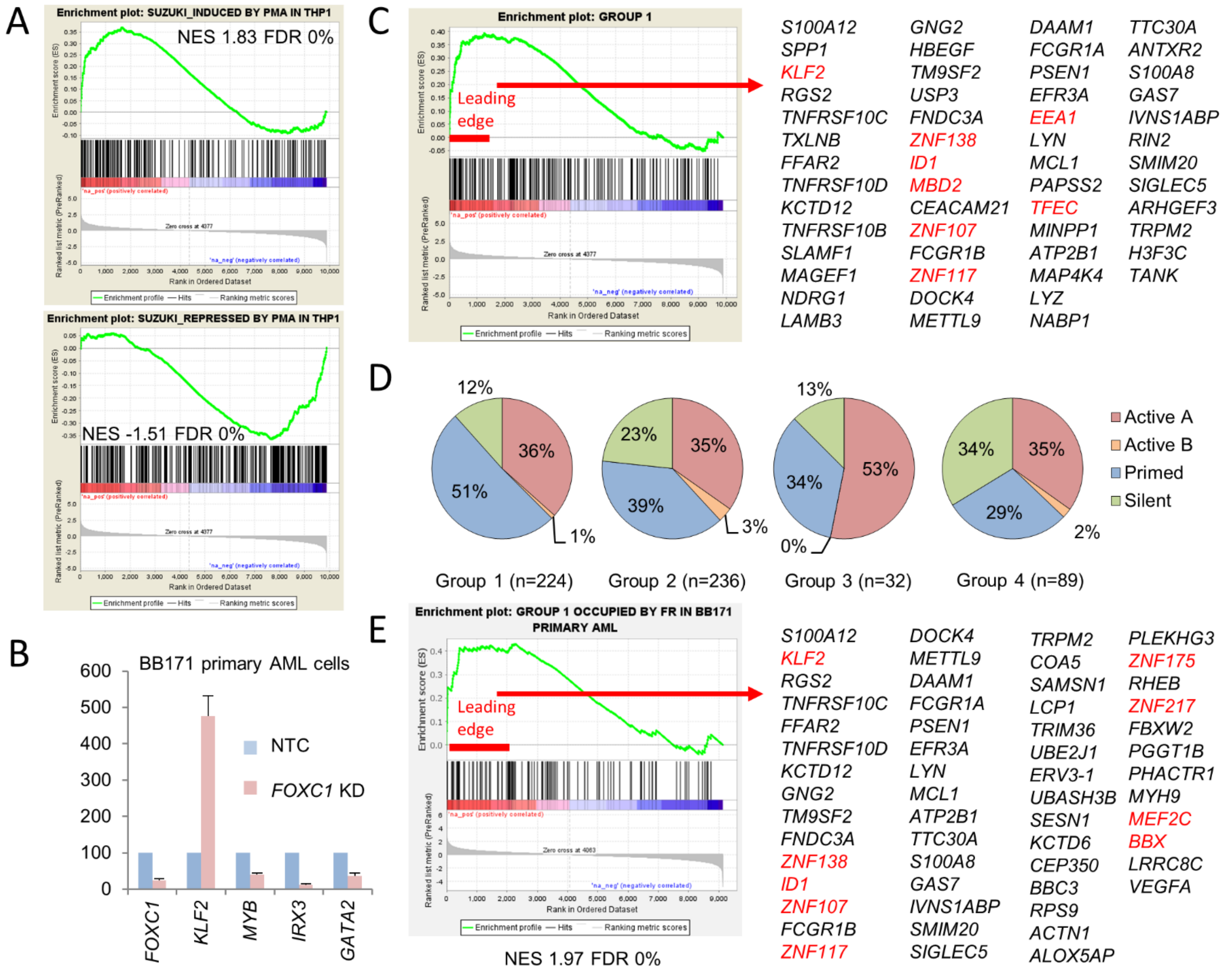

Figure S5. Gene expression and enhancer binding changes after *FOXC1* knockdown. Related to Figure 5.

(A) GSEA plots. NES, normalized enrichment score: FDR, false discovery rate. (B) Bar chart shows mean+SEM relative expression of the indicated genes in BB171 primary AML cells on D5 after initiation of FOXC1 KD. (C) Leading edge analysis of genes located near Group 1 FR-20 enhancers. Genes shown in red code for transcription regulators. (D) Pie charts show chromatin categories of the four FR-20 enhancer groups. (E) GSEA plot and leading edge analysis of Group 1 genes which are also co-bound by FOXC1 and RUNX1 in primary AML cells (BB171).

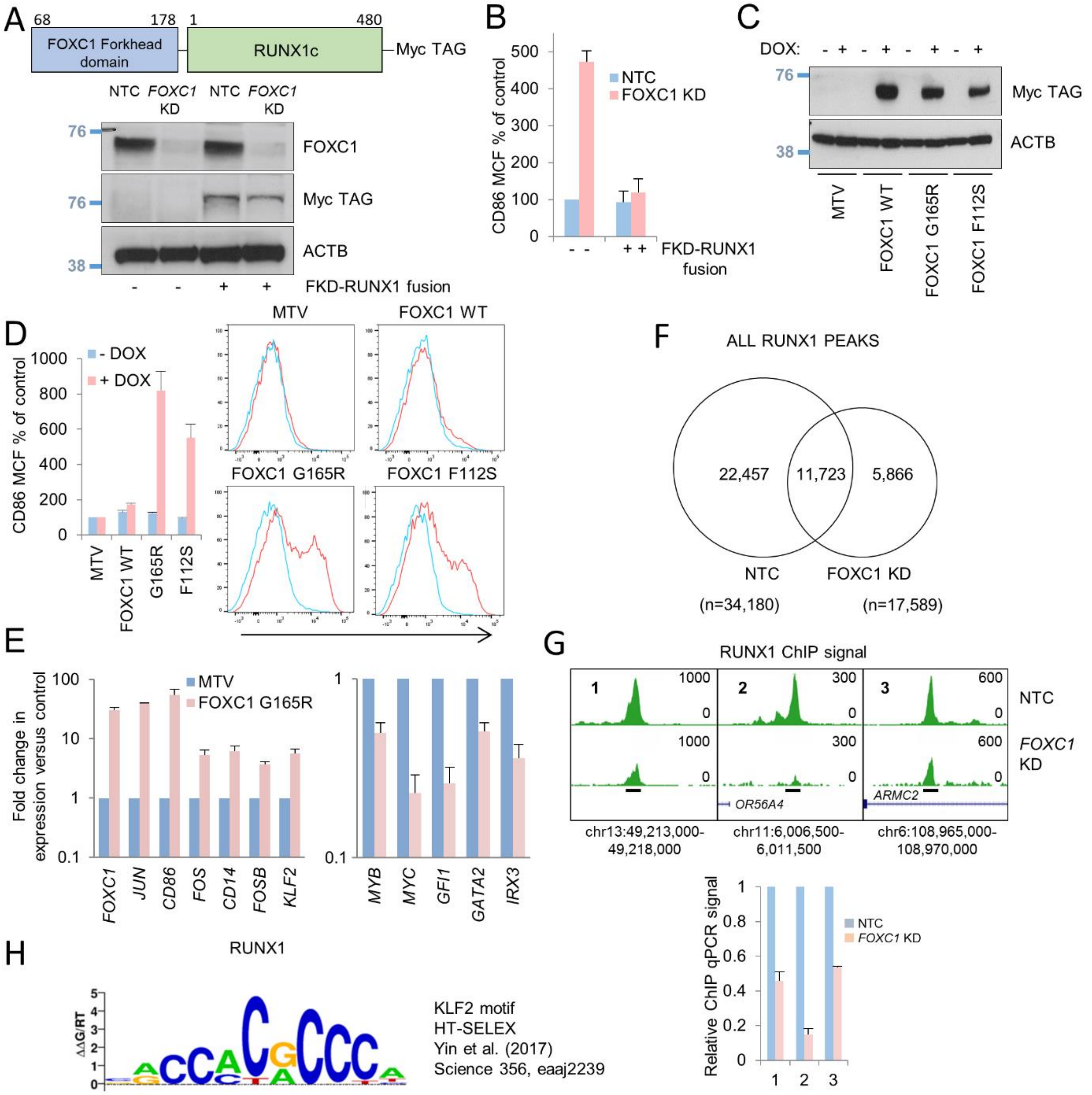

Figure S6. Forced recruitment or displacement of RUNX1 from FOXC1 binding sites regulates expression of differentiation genes; *FOXC1* knockdown triggers redistribution of RUNX1 binding. Related to Figure 6.

(A-B) Fujioka cells were infected with lentiviruses expressing a FOXC1 Forkhead domain–RUNX1c fusion protein under the control of a doxycycline-regulated promoter. (A) Western blots show expression of the indicated protein and transcription factors constructs. (B) Bar chart shows mean+SEM CD86 mean cell fluorescence (MCF) as determined by flow cytometry in the indicated conditions (n=3). (C-E) Fujioka cells were infected with lentiviruses expressing sequences coding for FOXC1 WT, FOXC1 G165R and FOXC1 F112S under the control of a doxycycline-regulated promoter. (C) Western blots show expression of the indicated proteins. (D) Bar chart (left panel) shows mean+SEM CD86 mean cell fluorescence as determined by flow cytometry in the indicated conditions (n=3). Right panel: representative flow cytometry plots. (E) Bar chart shows mean+SEM relative expression of the indicated genes in Fujioka cells of Day 4 following induced expression of FOXC1 G165R. (F) Venn diagram shows intersection of all RUNX1 binding peaks in control (NTC) or *FOXC1* KD Fujioka AML cells. (G) Exemplar ChIPseq tracks at three genomic locations (upper panel) with confirmatory ChIP-PCR for the indicated proteins (lower panel); mean+SEM relative ChIP signal is shown (n=3). (H) KLF2 binding motif.

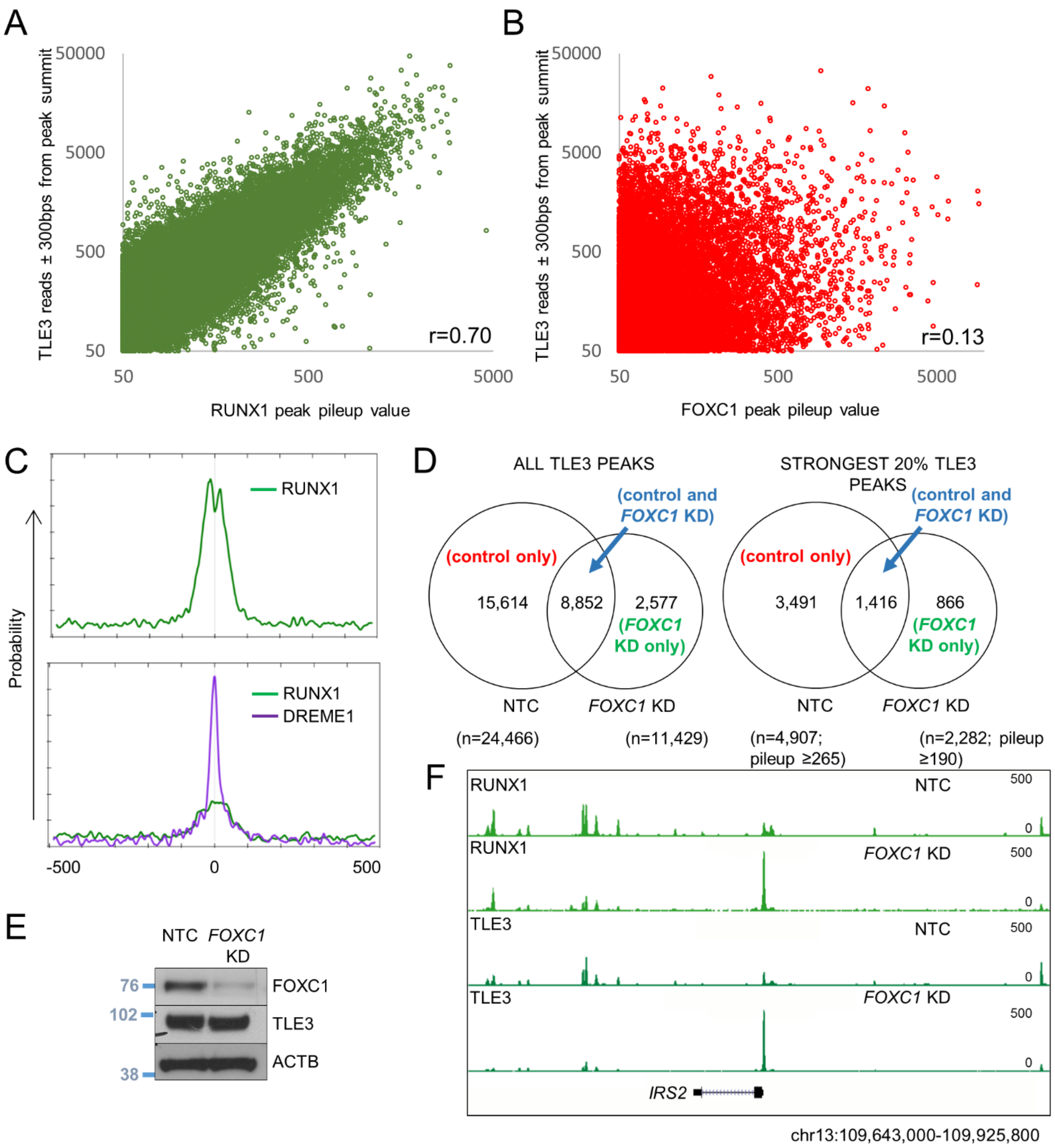

Figure S7. Correlation of RUNX1 and TLE3 ChIP signal and redistribution upon *FOXC1* KD. Related to Figure 7.

Dot plots show TLE3 ChIP signal at (A) RUNX1 and (B) FOXC1 binding peaks in control Fujioka AML cells. (C) MEME-ChIP motif enrichment plots at the strongest 20% of TLE3 binding peaks in control (upper panel) and *FOXC1* KD Fujioka AML cells (lower panel). The DREME1 motif is shown in Figure 7. (D) Venn diagrams show intersection of all (left panel) or the strongest 20% (right panel) of TLE3 binding peaks in control (NTC) or FOXC1 KD Fujioka AML cells. (E) Western blots for the indicated proteins in Fujioka cells on Day 4 following initiation of FOXC1 knockdown (panels for FOXC1 and ACTB are from the same experiment as shown in Figure S4I). (F) Exemplar ChIP-seq tracks.

Table S1. Karyotype & mutations of Fujioka AML cells and primary patient AML samples. Related to Figures 1-7.

Cell line name or biobank number	BM or PB	Karyotype	Identified mutations
Fujioka AML cells		50,X,?add(Y)(q11.2),del(2q)(q35q37),del(4)(q2?8),7,der(7)t(3;7)(q12;q32),inv(9)(p13q12)?c,t(10;11)(p12;q21),+13,+13,+del(13)(q12q14),add(18)(q?21),?+add(18)(q?23),+19	EZH2 p.A736fs, TP53 R196X, ETV6 R399C, NRAS G12C, TP53 Y236C
BB14	PB	46,XY [20]	SFSR2 P95R, ASXL1 G646Wfs*, FLT3 D835E, BCORL1 S953X, CEBPA P189del
BB53	PB	46,XY [20]	FLT3-ITD, BPA P112Sfs, K313dup
BB138	BM	46,XX [20]	NPM1 L287fs, NRAS G12A/G12S/G13D, RAD21 W18X, NOTCH1 V2229
BB140	PB	46,XY [20]	FLT3-ITD, NPM1 L287fs
BB148	PB	47,XY,+11[1]/48,sl,+8[7]/49,sdl,+4[2]	KRAS G12V G13D
BB160	PB	46,XY [20]	IDH2 R140Q, BCOR L884P
BB161	PB	46,XY,t(6;11)(q27;q23)[10]/48,idem,+der(6)t(6;11),+21[4]	SRSF2 P95H, ASXL1 T655Pfs*63, IDH2 R140Q, NMP1 W288Cfs*12, GATA2 G200Vfs*18, CEBPA P14L
BB165	BM	46,XX,t(8;22)(p11;q13),del(9)(q13q32)[10]	TET2 G1754R, DNTM3A R55H
BB171	PB	46,XX [20]	IDH1 R132H EZH2 P432LFS*31
BB187	BM	47,XY,+8[5]/46,XY[5]	SRSF2 P95R, IDH1 R132C, DNMT3A R882H, RUNX1 Y414Ffs*187, PHF6 A288_I290del, BCOR P910L
BB189	PM	46,XX [20]	DNMT3A R882L, NPM1 L287fs, FLT-ITD
BB232	PB	46,XX [20]	ASXL1 T655Pfs*63 (44bp ins), IDH1 R132C, DNMT3A R882H, WT1<br>R302Lfs*3/S313Lfs*70, NOTCH1 A1778V, CELSR2 T1454M, CSMD3 D2372E
BB247	PB	46,XY [20]	DNMT3A S349X, FLT3 D835V, NMP1 W288Cfs*12, GATA2 T354delinsTQ, CDKN2A I27L
BB380	PB	46,XX [20]	TET2 Q913Ffs*11, SH2B3 G451S
BB475	PB	46,XX [20]	IDH2 R140Q, DNMT3A R882H, FLT3-ITD, NPM1 W288C fs*12, RAD21 A544V
BB485	PB	46,XY [20]	IDH1 R132H, DNMT3A S714C, FLT3 D839G, NMP1 W288Cfs*12, KRAS Q61L, PTPN11 D61H
BB497	PM	Failed	DNMT3A R882H, FLT3-ITD, NPM1 W288Cfs*12, WT1 T382Ifs*9
BB539	BM	46,XY [20]	IDH1 R132H, NMP1 W290Efs*10, PTPN11 W290Efs*10
BB544	PM	46,XX [20]	FLT3 D835Y, NMP1 W288Cfs*12
BB546	PB	46,XY [20]	SF3B1 K666N, FLT3-ITD, WT1<br>V368fs, STAG2 K692R
BB556	PB	46,XY [20]	DNMT3A R882C, NMP1 L287fs, NRAS G13D, STAG2 T626fs
BB572	PB	46,XY [20]	TET2 T1091fs / Q1274E, ASLX1 G642fs, RUNX1 R201P / Y287fs / S318fs , KRAS A59E, BCOR E1185fs, ZRSR2 E79fs
BB575	PM	46,XX [20]	SRSF2 M89V, DNMT3A R882H, FLT3-ITD, NPM1 L287fs, SMC3 L242P
BB671	PB	46,XY [20]	DNMT3A R882H, FLT3-ITD, NPM1 W288Cfs*12
BB727	PB	Inv(16)	FLT3-ITD, FLT3 D835Y
BB764	BM	Failed	DNMT3A F755S, FLT3-ITD, FLT3 D835Y, NPM1 W288Cfs*12, PTPN11 E76K
BB773	PB	46,XY [20]	DNMT3A R882H, FLT3-ITD, NPM1 W288Cfs*12,
BB782	PB	46,XX,t(9;11)(p21.3;q23)[10]	No detected mutation
