# Supplementary material for: Enhancer recruitment of transcription repressors RUNX1 and TLE3 by mis-expressed FOXC1 blocks differentiation in acute myeloid leukemia
Source: Cell Rep. 2021 Sep 21;36(12):109725. doi: 10.1016/j.celrep.2021.109725 (PMC8480281; doi:10.1016/j.celrep.2021.109725)
Supplement: Table S4. Oligonucleotide sequences [file mmc4.docx]

**Table S4. Oligonucleotide sequences**

*Oligonucleotide sequences for lentiviral knockdown vectors*

*ADNP* KD1

F: ccggtggcagcttagccggagttaactcgagttaactccggctaagctgccattttttg

R: aattcaaaaatggcagcttagccggagttaactcgagttaactccggctaagctgcca

*ADNP* KD2

F: ccggatcgaagaccatgaacgtatactcgagtatacgttcatggtcttcgattttttg

R: aattcaaaaaatcgaagaccatgaacgtatactcgagtatacgttcatggtcttcga

*ARID3A* KD1

F: ccggccctgaaccaagatcactgaactcgagttcagtgatcttggttcagggtttttg

R: aattcaaaaaccctgaaccaagatcactgaactcgagttcagtgatcttggttcaggg

*ARID3A* KD2

F: ccggtggacttacgaggagcagtttctcgagaaactgctcctcgtaagtccatttttg

R: aattcaaaaatggacttacgaggagcagtttctcgagaaactgctcctcgtaagtcca

*CBFB* KD1

F: ccgggcgagtgtgagattaagtacactcgagtgtacttaatctcacactcgcttttttg

R: aattcaaaaagcgagtgtgagattaagtacactcgagtgtacttaatctcacactcgc

*CBFB* KD2

F: ccggggagaacagcgacaaacacctctcgagaggtgtttgtcgctgttctcctttttg

R: aattcaaaaaggagaacagcgacaaacacctctcgagaggtgtttgtcgctgttctcc

*CEBPA* KD1

F: ccgggctggagctgaccagtgacaactcgagttgtcactggtcagctccagctttttg

R: aattcaaaaagctggagctgaccagtgacaactcgagttgtcactggtcagctccagc

*CEBPA* KD2

F: ccgggcacgagacgtccatcgacatctcgagatgtcgatggacgtctcgtgctttttg

R: aattcaaaaagcacgagacgtccatcgacatctcgagatgtcgatggacgtctcgtgc

*CEBPE* KD1

F: ccggcctttgcctaccctccacatactcgagtatgtggagggtaggcaaaggtttttg

R: aattcaaaaacctttgcctaccctccacatactcgagtatgtggagggtaggcaaagg

*CEBPE* KD2

F: ccgggcagtgaacaaagatagccttctcgagaaggctatctttgttcactgctttttg

R: aattcaaaaagcagtgaacaaagatagccttctcgagaaggctatctttgttcactgc

*ELF1* KD1

F: ccgggccacttcaaataggaatcaactcgagttgattcctatttgaagtggctttttg

R: aattcaaaaagccacttcaaataggaatcaactcgagttgattcctatttgaagtggc

*ELF1* KD1

F: ccgggcactgtaatcacttcagttactcgagtaactgaagtgattacagtgctttttg

R: aattcaaaaagcactgtaatcacttcagttactcgagtaactgaagtgattacagtgc

*ETV6* KD1

F: ccggccataagaacagaacaaacatctcgagatgtttgttctgttcttatggtttttg

R: aattcaaaaaccataagaacagaacaaacatctcgagatgtttgttctgttcttatgg

*ETV6* KD2

F: ccgggcgccactactacaaactaaactcgagtttagtttgtagtagtggcgctttttg

R: aattcaaaaagcgccactactacaaactaaactcgagtttagtttgtagtagtggcgc

*HOXA10* KD1

F: ccggtcgcccatagacctgtggctactcgagtagccacaggtctatgggcgatttttg

R: aattcaaaaatcgcccatagacctgtggctactcgagtagccacaggtctatgggcga

*HOXA10* KD2

F: ccggctcacggacagacaagtgaaactcgagtttcacttgtctgtccgtgagtttttg

R: aattcaaaaactcacggacagacaagtgaaactcgagtttcacttgtctgtccgtgag

*IKZF1* KD1

F: ccggccgttggtaaacctcacaaatctcgagatttgtgaggtttaccaacggtttttg

R: aattcaaaaaccgttggtaaacctcacaaatctcgagatttgtgaggtttaccaacgg

*IKZF1* KD2

F: ccgggccgaagctataaacagcgaactcgagttcgctgtttatagcttcggctttttg

R: aattcaaaaagccgaagctataaacagcgaactcgagttcgctgtttatagcttcggc

*MAX* KD1

F: ccggacacacaccagcaagatattgctcgagcaatatcttgctggtgtgtgttttttg

R: aattcaaaaaacacacaccagcaagatattgctcgagcaatatcttgctggtgtgtg

*MAX* KD2

F: ccggccacagaatatatccagtatactcgagtatactggatatattctgtggtttttg

R: aattcaaaaaccacagaatatatccagtatactcgagtatactggatatattctgtgg

*RUNX1* KD1

F: ccggcctcgaagacatcggcagaaactcgagtttctgccgatgtcttcgaggtttttg

R: aattcaaaaacctcgaagacatcggcagaaactcgagtttctgccgatgtcttcgagg

*RUNX1* KD2

F: ccgggaaccactccactgcctttaactcgagttaaaggcagtggagtggttctttttg

R: aattcaaaaagaaccactccactgcctttaactcgagttaaaggcagtggagtggttc

*SPI1* KD1

F: ccggcggatctataccaacgccaaactcgagtttggcgttggtatagatccgtttttg

R: aattcaaaaacggatctataccaacgccaaactcgagtttggcgttggtatagatccg

*SPI1* KD2

F: ccggcaagaagaagatccgcctgtactcgagtacaggcggatcttcttcttgtttttg

R: aattcaaaaacaagaagaagatccgcctgtactcgagtacaggcggatcttcttcttg

*STAT3* KD1

F: ccgggcaaagaatcacatgccacttctcgagaagtggcatgtgattctttgctttttg

R: aattcaaaaagcaaagaatcacatgccacttctcgagaagtggcatgtgattctttgc

*STAT3* KD2

F: ccgggcacaatctacgaagaatcaactcgagttgattcttcgtagattgtgctttttg

R: aattcaaaaagcacaatctacgaagaatcaactcgagttgattcttcgtagattgtgc

*TLE3* KD

F: ccggcctatggcttgaacattgaaactcgagtttcaatgttcaagccataggtttttg

R: aattcaaaaacctatggcttgaacattgaaactcgagtttcaatgttcaagccatagg

*FOXN2* KD1

F: ccgggaagccatggcaaggttaatgctcgagcattaaccttgccatggcttctttttg

R: aattcaaaaagaagccatggcaaggttaatgctcgagcattaaccttgccatggcttc

*FOXN2* KD2

F: ccggtggtgtgttgatccggaatatctcgagatattccggatcaacacac

R: aattcaaaaatggtgtgttgatccggaatatctcgagatattccggatcaacacacca

*FOXK2* KD1

F: ccggcgagttcgagtatctgatgaactcgagttcatcagatactcgaactcgtttttg

R: aattcaaaaacgagttcgagtatctgatgaactcgagttcatcagatactcgaactcg

*FOXJ3* KD1

F: ccggtggaagtgtgcatagttatacctcgaggtataactatgcacacttccatttttg

R: aattcaaaaatggaagtgtgcatagttatacctcgaggtataactatgcacacttcca

*FOXJ3* KD2

F: ccgggaatcagtctctcaatcattactcgagtaatgattgagagactgattctttttg

R: aattcaaaaagaatcagtctctcaatcattactcgagtaatgattgagagactgattc

*Oligonucleotides for tetracycline inducible FOXC1 lentiviral expression:*

F: cacGAATTCACCATGCAGGCGC

R:cacACTAGTtcacagatcctcttctgagatgagtttttgttcacccgaaccCACAGATCCTCTTCTGA

*Oligonucleotides for generation of FOXC1 deletion mutants for lentiviral expression:*

FOXC1 Δ(1-50)

F: cacgaattcaccatgGCGCACGCCGAGCAGTAC

R: cacACTAGTtcacagatcctcttctgagatgagtttttgttcacccgaaccCACAGATCCTCTTCTGA

FOXC1 Δ(436-553)

F: cacGAATTCACCATGCAGGCGC

R:cacACTAGTtcacagatcctcttctgagatgagtttttgttcacccgaaccCGGAGGCAGAGAGTAGTCGG

*Primer sequences for generation of FOXC1 deletion mutants for lentiviral expression:*

FOXC1 Δ(69-178)

F: cgggccctacacggcggtgaaggaca

R: tgtccttcaccgccgtgtagggcccg

FOXC1 Δ(215-366)

F: caacgcgcccggtcagacctccagcg

R: cgctggaggtctgaccgggcgcgttg

FOXC1 G165R

F: aacatgttcgagaaccgcagcttcctgcggcgg

R: ccgccgcaggaagctgcggttctcgaacatgtt

FOXC1 F112S

F: ccagttcatcatggaccgctcccccttctaccggg

R: cccggtagaagggggagcggtccatgatgaactgg

*Primer sequences for generation of doxycycline-inducible FOXC1-DBD RUNX1c*

F: atagaattcgccaccatggaacaaaaactcatctcagaagaggatctgccgcagccgcagcccaag

R: atagctagcacccgaaccgtccttcttcttgaagcgccgccg

*Primers used for amplification of full length RUNX1c:*

F: atagctagcatggcttcagacagcatatttgagtc

R: ataatcgattcagtagggcctccacacgg

*Primer sequences for generation of RUNX1 deletion mutants :*

RUNX1b WT
F: gagaagatctGCCACCATGGATTACAAGGATGACGACGATAAGcgtatccccgtagatg

R: gagactcgagTCAgtagggccgccacacgg

RUNX1b Δ(1-56)

F: gagaagatctGCCACCATGGATTACAAGGATGACGACGATAAGgaccaccctggcgag

R: gagactcgagTCAgtagggccgccacacgg

RUNX1b Δ(242-451)

F: gagaagatctGCCACCATGGATTACAAGGATGACGACGATAAGcgtatccccgtagatg

R: gagactcgagTCActgcatctgactttgag

RUNX1b Δ(372-451)

F: gagaagatctGCCACCATGGATTACAAGGATGACGACGATAAGcgtatccccgtagatg

R: gagactcgagTCAgccggtctggaaggg

RUNX1b Δ(186-241)

F: ggcagaaactagatgatgatgccaggcagatcc

R: ggatctgcctggcatcatcatctagtttctgcc

RUNX1b Δ(50-175)

F: gagcggcgaccgcccccgaagacatc

R: gatgtcttcgggggcggtcgccgctc

*Primers used for generation of KLF2 lentiviral expression construct:*

F: cacgaattcaccatggcgctgagtgaacccatc

R: cactctagatcacatgtgccgtttcatgtgcagc

*Primers and probe sets used for quantitative PCR for the indicated genes:*

*ACTB*: F: ATTGGCAATGAGCGGTTC, R: GGATGCCACAGGACTCCAT, probe 11

*ADNP*: F: GGGTGACCTCACAGGTGTTC, R: ACTGGCATTTGGGACCTG, probe 32

*ARID3A*: F: CCACGGCGACTGGACTTA, R: GCTGAACAAGTCATCCAGGAAT, probe 68

*CBFB*: F: ACTGGATGGTATGGGCTGTC, R: AAGGCCTGTTGTGCTAATGC, probe 3

*CD14*: F: CAAGTAGATTCTCTGGGATATAAGGAA, R: CCTCCTCTGTGAACCCTGAT, probe 69

*CD86*: F: GGAATGCTGCTGTGCTTATG, R: GAATGTTACTCAGTCCCATAGTGC, probe 54

*CEBPA*: TaqMan Gene Expression Assay, Assay ID Hs00268872_s1

*CEBPE*: F: CTCTGCGCGTTCTCAAGG, R: GCCGGTACTCAAGGCTATCTT, probe 8

*ELF1*: F: TGTTGTCCAACAGAACGACCT, R: GGAAAAATAGCTGGATCACCA, probe 88

*ETV6*: F: CCCTGCGCCACTACTACAA, R: TGATTTCATCTGGGGTTTTCA, probe 12

*FOS* F: AAGGAGAATCCGAAGGGAAA, R: GTGTATCAGTCAGCTCCCTCCT, probe 46

*FOSB*: F: AAGAGGTACAGCGGCATCC, R: CGTTCCAACAATGGCAAAGT, probe 4

*FOXC1*: F: TGAACGGGAATAGTAGCTGTCA, R: GGACGTGCGGTACAGAGAC, probe 11

*GATA2*: F: GATGAGCATCCTGCGAGTG, R: CACACAGCACATCCACCCTA, probe 10

*GFI1*: TaqMan Gene Expression Assay, Assay ID Hs00382207_m1

*HOXA10*: TaqMan Gene Expression Assay, Assay ID Hs00172012_m1

*IKZF1*: F: CAATGTGCTCATGGTTCACAA, R: GTTGCCCTTCTGGGTGAAT, probe 47

*IRX3*: F: AAAAGTTACTCAAGACAGCTT, R: GGATGAGGAGAGAGCCGATA, probe 57

*JUN*: TaqMan Gene Expression Assay, Assay ID Hs01103582_s1

*KLF2*: F: TGGTCTGGTTGCTTGGAACT, R: CTGCCCTCCATCAAACTCTC, probe 52

*MAX*: F: CGGTGGGTACAAGATGACG, R: CTTGCGGGTGCTTTCTACA, probe 39

*MYB*: TaqMan Gene Expression Assay, Assay ID Hs00920556_m1

*MYC*: TaqMan Gene Expression Assay, Assay ID hs00153408_m1

*RUNX1*: TaqMan Gene Expression Assay, Assay ID Hs01021970_m1

*SPI1:* F: CCACTGGAGGTGTCTGACG, R: CTGGTACAGGCGGATCTTCT, probe 51

*STAT3*: F: GAGCAGAGATGTGGGAATGG, R: CGGTCTCAAAGGTGATCAGG, probe 17

*TLE3*: F: TGGTGAGCTTTGGAGCTGT, R: ACATGGAATGAGTACGCTGGT, probe 65

*FOXN2*: F: TCCATATTTTGCTACTGCACCA, R: CATAAGGAACCTTTTCCATTAACC, probe 11

*FOXK2*: F: AGCATCTGAGGTGGTCACG, R: CAACATGTGCAAAGGACTCG, probe 61

*FOXJ3*: F: AGCACTCCAGGAACAACGAT, R: AAGGCTTGGGAAGGCATC, probe 21

*KLF2 enhancer deletion:*

Negative control: GCACUACCAGAGCUAACUCA

26F: GACCAGTCTGGAAACCCACCTGG

21R: GGTCTCCTCTGCTCTCCAGGTGG

492F: AATGTTGGCCCCTCGACCTGGGG

491R: GAATGTTGGCCCCTCGACCTGGG

*Primers for detection of KLF2 enhancer deletion:*

Deletion_F: CTGGAAGGGAGTGGTAGCTT

Deletion_R: GAGTCCTTCTTCTGCCCCTG

*Primers and probe sets used for ChIP qPCR:*

GROUP A

1: F: AGAGTGGCACCAGCCTACAG, R: GTCTTGCAAAACCGGAAGC, probe 87

2: F: CCAGTTCAACCACATCCTGA, R: AACCAGTATGAAATGGAGCAAAA, probe 72

3: F: GGGGCAGTGTACCTGGAAG, R: CCCAGATACCAAGGGGTGA, probe 27

GROUP C

1: F: CGCACACACACAGCAAAAG, R: AAGGGCTAGAAGTACAGCTGAGAT, probe 34

2: F: CACGAGCTCGATGTGTCG, R: TGAAAGGGAAACAGAAAGTCG, probe 30

3: F: CGTCTTAATACAGTGAAAGAATTGAGG, R: CGTTCCCGTCTTTCAAACC, probe 77
