# Supplementary material for: Enhancer recruitment of transcription repressors RUNX1 and TLE3 by mis-expressed FOXC1 blocks differentiation in acute myeloid leukemia
Source: Cell Rep. 2021 Sep 21;36(12):109725. doi: 10.1016/j.celrep.2021.109725 (PMC8480281; doi:10.1016/j.celrep.2021.109725)
Supplement: Document S2. Article plus supplemental information [file mmc5.pdf]

Cell Reports

Article

Enhancer recruitment of transcription repressors RUNX1 and TLE3 by mis-expressed FOXC1 blocks differentiation in acute myeloid leukemia

Graphical abstract

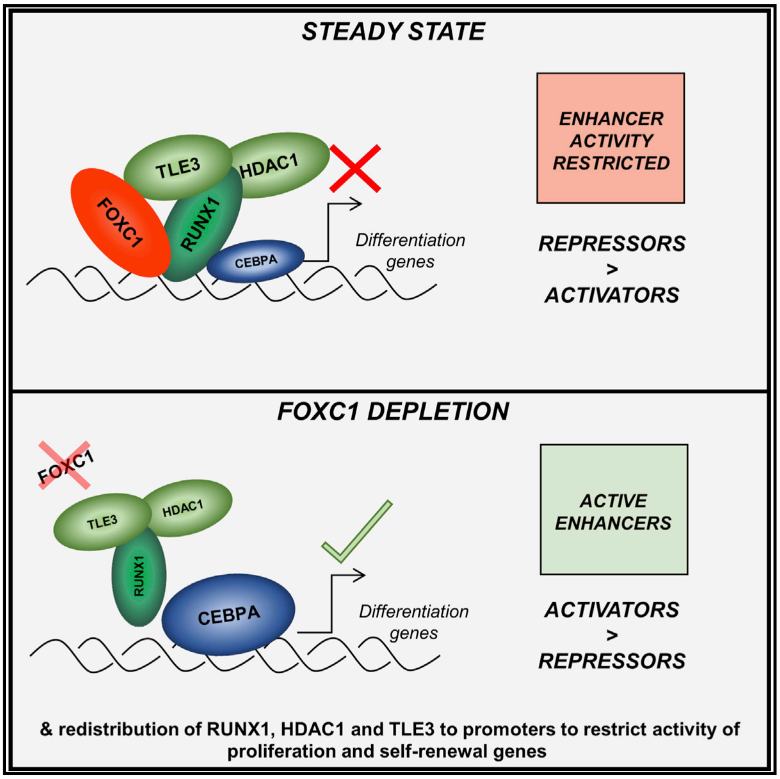

Highlights

- FOXC1 contributes to a monocyte-macrophage lineage differentiation block in AML
- FOXC1 and RUNX1 colocalize on chromatin via interaction of their DNA binding domains
- A FOXC1/RUNX1/TLE3 repressor complex limits monocyte gene enhancer activity
- FOXC1 depletion in AML initiates widespread redistribution of RUNX1/TLE3 on chromatin

Authors

Fabrizio Simeoni, Isabel Romero-Camarero, Francesco Camera, ..., Daniel H. Wiseman, Jason S. Carroll, Tim C.P. Somervaille

Correspondence

tim.somervaille@cruk.manchester.ac.uk

In brief

Simeoni et al. demonstrate that the Forkhead factor FOXC1 recruits a repressor complex of RUNX1, HDAC1, and Groucho family member TLE3 to a discrete set of enhancers distributed close to genes controlling monocyte/ macrophage differentiation, thereby contributing to the differentiation block, which is the pathognomonic feature of acute myeloid leukemia.

Simeoni et al., 2021, Cell Reports *36*, 109725
September 21, 2021 © 2021 The Authors.
https://doi.org/10.1016/j.celrep.2021.109725

CellPress

Cell Reports

CellPress
OPEN ACCESS

Article

Enhancer recruitment of transcription repressors RUNX1 and TLE3 by mis-expressed FOXC1 blocks differentiation in acute myeloid leukemia

Fabrizio Simeoni,[1] Isabel Romero-Camarero,[1] Francesco Camera,[1] Fabio M.R. Amaral,[1] Oliver J. Sinclair,[1] Evangelia K. Papachristou,[2] Gary J. Spencer,[1] Michael Lie-A-Ling,[3] Georges Lacaud,[3] Daniel H. Wiseman,[4] Jason S. Carroll,[2] and Tim C.P. Somervaille[1,5,*]

[1]Leukaemia Biology Laboratory, Cancer Research UK Manchester Institute, The University of Manchester, Manchester M20 4GJ, UK
[2]Cancer Research UK Cambridge Institute, Cambridge CB2 0RE, UK
[3]Stem Cell Biology Group, Cancer Research UK Manchester Institute, The University of Manchester, Macclesfield SK10 4TG, UK
[4]Epigenetics of Haematopoiesis Group, Oglesby Cancer Research Building, The University of Manchester, Manchester M20 4GJ, UK
[5]Lead contact
*Correspondence: tim.somervaille@cruk.manchester.ac.uk
https://doi.org/10.1016/j.celrep.2021.109725

SUMMARY

Despite absent expression in normal hematopoiesis, the Forkhead factor FOXC1, a critical mesenchymal differentiation regulator, is highly expressed in ~30% of HOXA^{high} acute myeloid leukemia (AML) cases to confer blocked monocyte/macrophage differentiation. Through integrated proteomics and bioinformatics, we find that FOXC1 and RUNX1 interact through Forkhead and Runt domains, respectively, and co-occupy primed and active enhancers distributed close to differentiation genes. FOXC1 stabilizes association of RUNX1, HDAC1, and Groucho repressor TLE3 to limit enhancer activity: *FOXC1* knockdown induces loss of repressor proteins, gain of CEBPA binding, enhancer acetylation, and upregulation of nearby genes, including *KLF2*. Furthermore, it triggers genome-wide redistribution of RUNX1, TLE3, and HDAC1 from enhancers to promoters, leading to repression of self-renewal genes, including *MYC* and *MYB*. Our studies highlight RUNX1 and CEBPA transcription factor swapping as a feature of leukemia cell differentiation and reveal that FOXC1 prevents this by stabilizing enhancer binding of a RUNX1/HDAC1/TLE3 transcription repressor complex to oncogenic effect.

INTRODUCTION

Acute myeloid leukemia (AML) is a blood cancer characterized by a block to normal myeloid lineage differentiation. This results in accumulation of myeloid blast cells in bone marrow (BM) and blood with consequent failure of normal hematopoiesis (Khwaja et al., 2016). Although the range of balanced translocations, point mutations, and indels associated with this malignancy is largely characterized, the mechanisms by which these genetic lesions confer a differentiation block is less well understood. This is emphasized by studies that show that many AML-associated mutations, including some chromosomal abnormalities, may be found in chemotherapy-treated patients in complete remission, in patients with myelodysplasia prior to evolution to AML, or in aging individuals with normal blood counts (i.e., clonal hematopoiesis of indeterminate potential) (Wiseman et al., 2016; Sperling et al., 2017; Jongen-Lavrencic et al., 2018; Jaiswal and Ebert, 2019). This is consistent with an emergent theme in AML that many disease-associated mutations promote expansion of hematopoietic stem and progenitor cells (HSPCs) that otherwise retain relatively normal differentiation potential, rather than immediately conferring a differentiation block (Challen and Goodell, 2020). Few AML-associated genetic lesions are exclusively found in AML, and even those such as *FLT3* internal tandem duplications or *NPM1* mutations, which are rarely found in clinical contexts other than AML, yield prominent myeloproliferative phenotypes when modeled in mice (Kelly et al., 2002; Vassiliou et al., 2011). Even murine models of *MLL* fusions often exhibit a prominent antecedent myeloproliferation ahead of pre-terminal acute leukemic transformation (Warren et al., 1994; Somervaille et al., 2009). The presence of certain combinations of genetic lesions within a long-lived progenitor cell is likely necessary for the generation of a differentiation block, but how mutations co-operate to arrest normal differentiation is often unclear. Improved understanding of the mechanisms involved will facilitate development of therapeutic approaches to promote differentiation, an approach already exemplified by all-*trans* retinoic acid in the treatment of acute promyelocytic leukemia (Khwaja et al., 2016). In addition to killing leukemia cells with chemotherapy, induction of differentiation is a major goal of treatment.

We previously reported that the Forkhead family transcription factor gene *FOXC1*, which is a critical regulator of normal mesenchymal and mesodermal differentiation, is highly expressed in

This is an open access article under the CC BY license (http://creativecommons.org/licenses/by/4.0/).

around 20% of cases of AML, but not expressed in normal hematopoietic lineages (Somerville et al., 2015). Although it indirectly regulates hematopoietic stem cells (HSCs) through controlling the function of HSC niche cells, it makes no cell-intrinsic contribution to blood cell function (Omatsu et al., 2014). High *FOXC1* expression in AML is almost invariably found in association with high *HOXA/B* gene expression, and ~30% of human *HOXA/B*-expressing AML cases (e.g., those with *NPM1* mutations, *MLL*-fusions, or a t(6;9) translocation) exhibit high *FOXC1* expression. *In vitro* and *in vivo* experimental evidence confirm that FOXC1 confers a monocyte/macrophage lineage differentiation block and sustains clonogenic activity in both murine and primary human *FOXC1*^{high} *HOXA*^{high} AML cells. Co-expression of *FOXC1* with *Hoxa9* accelerates the onset of AML in murine modeling, with the resulting leukemias exhibiting a higher level of differentiation block by comparison with those initiated by *Hoxa9* alone. Further, patients with high *FOXC1* expression exhibit inferior survival (Somerville et al., 2015). More widely, high-level *FOXC1* expression is also observed in a multitude of solid malignancies, including breast, colorectal, cervical, gastric, and liver cancers (Gilding and Somervaille, 2019), where functional experiments confirm that it promotes increased migration and metastasis and, as in AML, typically confers an inferior survival.

Despite the importance of FOXC1 in human AML, and more broadly in solid malignancies, the mechanisms by which FOXC1 confers adverse outcomes in human cancers remain largely unexplored. To begin to address this in AML, we performed an integrated analysis of the protein-protein interactions and genome-wide binding sites of FOXC1 in human myeloid leukemia cells.

RESULTS

FOXC1 confers a differentiation block in human AML cells

We first determined *FOXC1* expression levels in a panel of AML cell lines and primary AML samples by quantitative PCR (qPCR) (Figures S1A and S1B). Of the cell lines tested, the highest *FOXC1* transcript levels were observed in Fujioka cells. These are derived from a child with acute monocytic leukemia and exhibit a t(10;11) translocation indicative of a *CALM-AF10* fusion, as well as mutations in *NRAS*, *ETV6*, *TP53*, and *EZH2*, among others (Table S1; Hirose et al., 1982; Narita et al., 1999). To confirm that FOXC1 contributes to the differentiation block exhibited by Fujioka cells, we performed *FOXC1* knockdown (KD) and observed differentiation, as evidenced by morphology, increased expression of the monocyte/macrophage lineage differentiation marker CD86, reduced clonogenic activity, a reduced proportion of cells in the SG2M phase of the cell cycle, as well as an increase in apoptosis (Figures 1A–1D and S1C–S1E). We confirmed that the KD phenotype was an on-target effect by co-expressing a *FOXC1* cDNA engineered by site-directed mutagenesis to generate KD-resistant transcripts (*FOXC1* SDM3) (Figures 1C–1E). We performed similar experiments in *FOXC1*^{high} primary human AML cells from a patient with normal karyotype AML with mutations in *NPM1*, *FLT3*, *DNMT3A*, and *IDH2* (BB475; Table S1), with similar results (Figures 1F–1I and S1F–S1H). Thus, in support of our prior conclusions and those of others (Somerville et al., 2015; Assi et al., 2019), misexpressed *FOXC1* confers a differentiation block in human AML cells.

Identification of chromatin-bound FOXC1 interacting proteins

To identify in an unbiased manner for FOXC1 interacting proteins with potential functional roles, we performed rapid immunoprecipitation mass spectrometry of endogenous protein (RIME) (Mohammed et al., 2016). We generated a polyclonal antibody to a version of human FOXC1 engineered to lack the Forkhead domain shared by other Forkhead family transcription factors. We performed three separate analyses, two in Fujioka cells and a third in primary AML blast cells (BB475), and identified 131 proteins present in all three experiments. We deemed these high-confidence FOXC1 interacting proteins (Figure 2A; Table S2). FOXC1 was the only Forkhead family member identified. As expected, there was strong enrichment for proteins with Gene Ontology biological process annotations such as "mRNA splicing, via spliceosome" ($p = 10^{-20}$), "ATP-dependent chromatin remodelling" ($p = 10^{-12}$), and "transcription from RNA polymerase II promoter" ($p = 10^{-10}$).

We focused our initial interest on the 12 transcription factors identified, because transcription factors are critical regulators of differentiation and cell fate. To determine which of these might be functionally linked to the differentiation block conferred by FOXC1, we performed KD of each gene in Fujioka cells; we included *CBFB*, which we also identified as a FOXC1 interacting protein, in view of its coding for the obligate heterodimeric binding partner of RUNX1. KD of *RUNX1*, *CBFB*, *CEBPA*, *STAT3*, and *CEBPE* using two separate short hairpin RNA (shRNA) hairpins for each gene increased expression of CD86, which we used as a surrogate marker for upregulation of a differentiation program (Figures 2B and S2A). Transcription factor interactions with FOXC1 identified by RIME may include those mediated by direct protein-protein interaction, as well as those mediated by a short intervening sequence of DNA (Figure 2A). To eliminate the latter, we performed confirmatory co-immunoprecipitation experiments in the presence of Benzonase endonuclease to remove DNA and RNA and noted that only RUNX1, CBFB, and CEBPA were pulled down by FOXC1 immunoprecipitation (Figure 2C). Domain mapping experiments demonstrated that FOXC1 interacted with RUNX1 and CEBPA through its Forkhead DNA binding domain (Figures 2D and 2E), and that the interactions were reduced by introduction of G165R and F112S mutations (Figure 2F). Although the G165R and F112S mutants that occur in the Axenfeld-Rieger syndrome (an autosomal dominant syndrome of congenital malformation of the human eye) are reported to retain DNA binding capacity, the residues are predicted by molecular modeling studies to sit opposite the DNA-binding interface, suggesting a role in protein-protein interaction; G165 resides within Wing 2 of the Forkhead binding domain (Murphy et al., 2004; Huang et al., 2008). In reciprocal analyses, we found that the FOXC1-RUNX1 interaction was mediated by the Runt domain of RUNX1 (Figures 2G and 2H).

It is interesting to note that although Forkhead transcription factor genes *FOXK2*, *FOXN2*, *FOXJ3*, and *FOXO3* are highly

CellPress
OPEN ACCESS

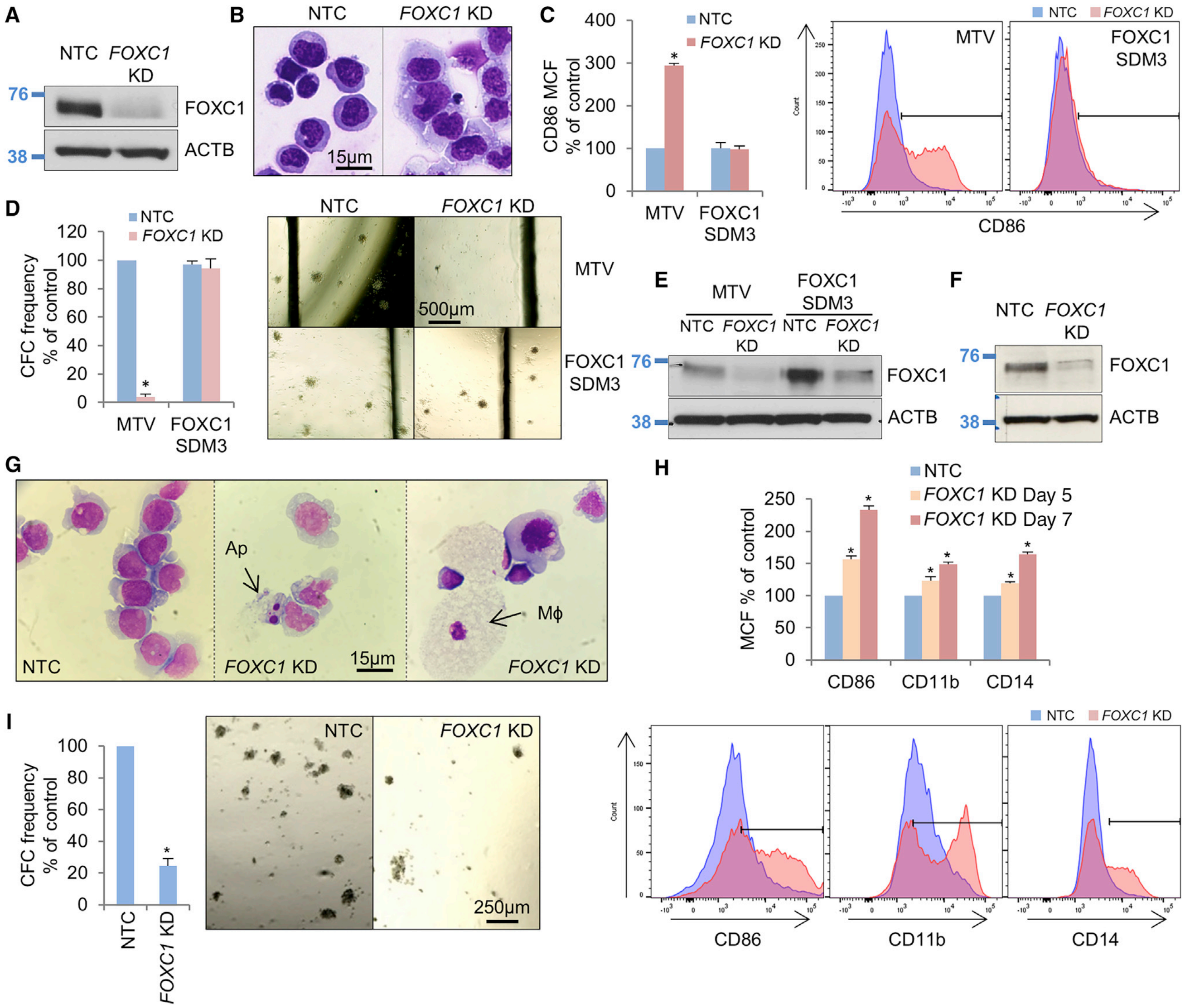

Figure 1. FOXC1 confers a differentiation block in human AML cells

(A–E) Human Fujioka AML cells were infected with a lentivirus targeting *FOXC1* for KD or a non-targeting control (NTC). (A) Western blot shows *FOXC1* KD 72 h after KD initiation. (B) Day 7 cytospins. (C) Bar chart (left panel) shows mean + SEM CD86 cell fluorescence (MCF) on day 5 (n = 3). Representative flow cytometry plots (right panel). (D) Bar chart (left panel) shows mean + SEM colony-forming cell (CFC) frequencies relative to control cells after 12 days in semi-solid culture (n = 3). Right panel: representative images. (E) Western blot.

(F–I) Primary patient AML cells (BB475) were infected with a lentivirus targeting *FOXC1* for KD or a NTC with puromycin drug resistance as selectable marker (n = 2). (F) Western blot shows *FOXC1* KD in BB475 AML cells 72 h following KD initiation. (G) Day 7 cytospins. (H) Bar chart (top panel) shows mean + SEM cell fluorescence (n = 2). Bottom panel: representative flow cytometry plots. (I) Bar chart (left panel) shows mean + SEM CFC frequencies of KD cells relative to control cells after 10 days in semi-solid culture (n = 2). Right panel: representative colonies. *$p < 0.05$ for the indicated comparisons by t test (C, D, and I) or one-way ANOVA with Fisher's least significant difference post hoc test (H).

Ap, apoptotic cell; Mϕ, macrophage; MTV, empty vector; SDM3, site-directed mutagenesis construct #3. See also Figure S1.

expressed in both Fujioka AML cells and FOXC1^{high} primary AMLs (Figures S2B and S2C), their gene products all lack the conserved Wing 2 amino acid sequence found in FOXC1 required for the RUNX1 and CEBPA interaction (Figure S2G). KD of *FOXK2*, *FOXN2*, and *FOXJ3* all failed to induce differentiation of Fujioka cells (Figures S2D–S2F). Depletion of FOXO3, which is predominantly cytoplasmic, is known to promote differentiation in AML through a mechanism involving increased stress-activated kinase signaling (Sykes et al., 2011).

Thus, FOXC1 interacts with CEBPA and with the Runt domain of RUNX1 through residues in its Forkhead domain, including the Wing 2 region, raising a question as to whether the functional effects of *FOXC1* misexpression in AML are mediated through its interaction with one or both of these proteins.

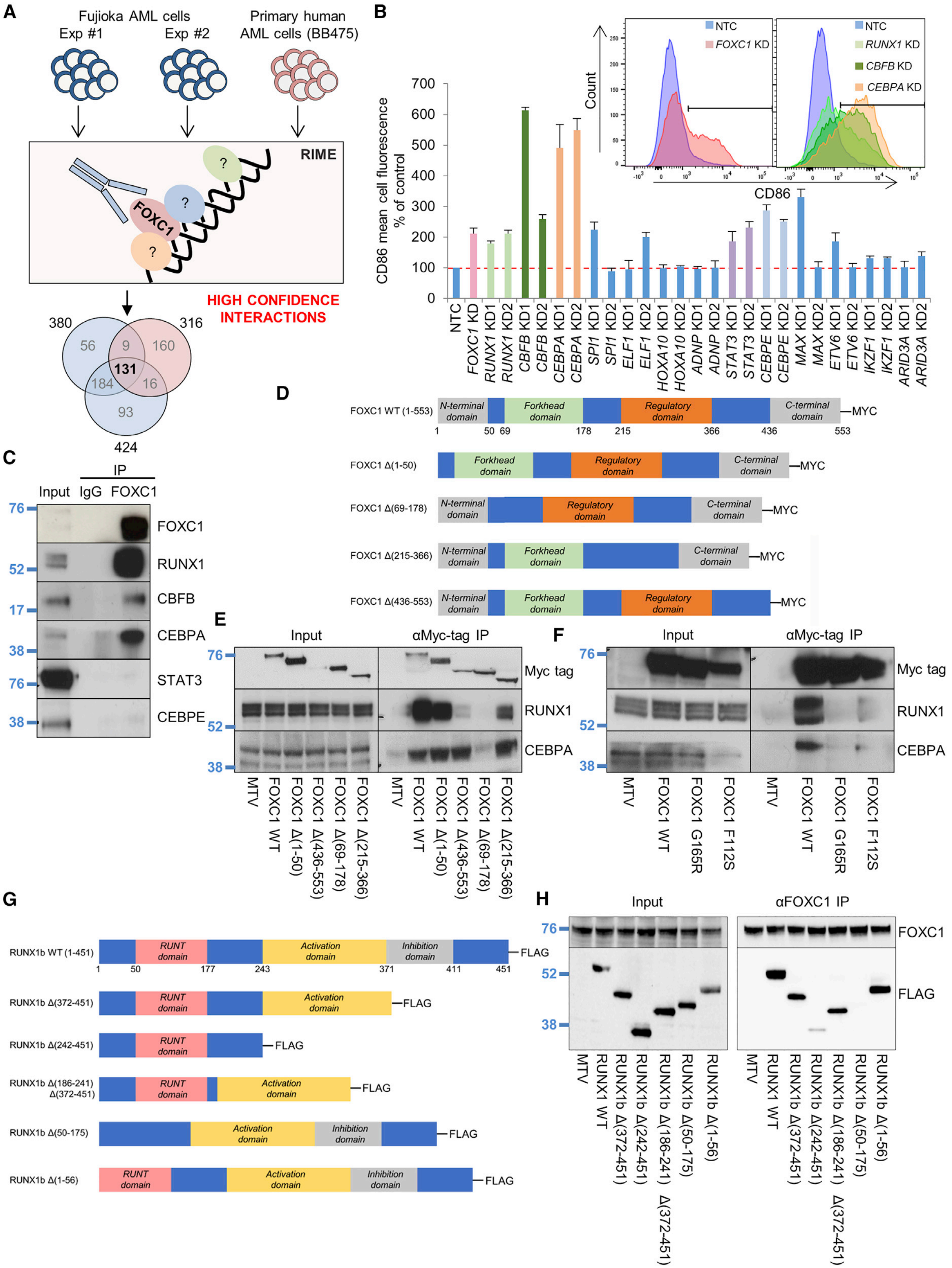

(legend on next page)

Genome-wide binding profiles of FOXC1, RUNX1, CEBPA, and SPI1

To identify FOXC1 binding sites genome wide and to determine their proximity to RUNX1 and CEBPA binding sites, we performed chromatin immunoprecipitation (ChIP) sequencing (ChIP-seq) for FOXC1, RUNX1, and CEBPA in Fujioka AML cells. In view of its critical role in myeloid development (Iwasaki et al., 2005), we also performed ChIP-seq for SPI1 (also known as PU.1).

In Fujioka cells, after excluding blacklisted genomic regions prone to artifact and making use of stringent threshold criteria (called peaks had pileup value $\geq$ 50 and fold enrichment over input $\geq$ 5), Model-based Analysis of ChIP-seq v2 (MACS2) (Zhang et al., 2008) identified 18,745 FOXC1 peaks, 34,180 RUNX1 peaks, 36,856 CEBPA peaks, and 34,717 SPI1 peaks. Multiple Expectation maximization for Motif Elicitation (MEME)-ChIP (Machanick and Bailey, 2011) confirmed that genomic sequences at the center of transcription factor binding peaks were strongly enriched for the appropriate consensus binding motif (Figure 3A). In all cases, the great majority of peaks were distributed over intronic and intergenic regions versus promoter regions (Figures 3B, S3A, and S3B), consistent with putative roles at enhancers. We next performed ChIP-seq for H3K27Ac and H3K4Me1 in Fujioka cells and categorized the chromatin surrounding each transcription factor binding peak as active A (H3K27Ac^{high}, H3K4Me1^{high}), active B (H3K27Ac^{high}, H3K4Me1^{low}), primed (H3K27Ac^{low}, H3K4Me1^{high}), or quiet (H3K27Ac^{low}, H3K4Me1^{low}) (Figures 3C, 3D, S3C, and S3D). Many active B sites were located at gene promoters (Figure S3E), in contrast with the other classes of binding site. Considering the strongest 20% of binding peaks by pileup value for each transcription factor, we found that 29% and 41% of CEBPA or RUNX1 peaks, respectively, were bound at sites of active chromatin (i.e., active A or active B), but consistent with its role as a pioneer factor, only 2% of SPI1 peaks. The reverse pattern was observed for quiet chromatin with 98%, 35%, and 24% of SPI1, CEBPA, and RUNX1 peaks, respectively, bound in these regions. Consistent with pioneer activity, as for FOXA transcription factors, and a dual role in regulating the function of primed and active enhancers, the chromatin distribution of the strongest 20% of FOXC1 binding sites showed an intermediate distribution: 59% of strong peaks were bound to quiet chromatin and 20% to active chromatin (Figure 3D). A similar pattern was observed when all transcription factor binding peaks for the four transcription factors were considered (Figure S3D). The differences in the strength and distribution of ChIP signal for H3K27Ac and H3K4Me1 surrounding the binding peaks of the four transcription factors are further demonstrated in the line and violin plots shown in Figure 3E. We also performed assay for transposase accessible chromatin (ATAC) sequencing in Fujioka cells and observed consistent findings: the strongest RUNX1 and CEBPA peaks bound more accessible chromatin, whereas the opposite was the case for SPI1 (Figures 3D and 3F). FOXC1 exhibited an intermediate pattern of association.

To confirm a similar distribution of FOXC1 binding sites in Fujioka cells by comparison with primary patient blast cells, we performed FOXC1 ChIP-seq in a normal karyotype AML sample (BB475; Table S1); MACS2 identified 39,941 FOXC1 peaks. There was a substantial overlap of FOXC1 binding peaks in the two cell populations with, for example, 85.6% of the strongest 20% of FOXC1 peaks in Fujioka cells being represented in the BB475 primary sample (Figures 4A and 4B). There was also a strong positive correlation of FOXC1 peak strength in the two samples (Figures 4A, 4C, and 4D). Of note, in *FOXC1* KD Fujioka cells MACS identified just 567 peaks, with no evidence of peak redistribution (Figure 4A). Thus, FOXC1 exhibits a mixed pattern of binding to quiet, primed, and active chromatin predominantly at intergenic and intronic locations, with largely overlapping binding sites in primary and Fujioka AML cells.

Close physical interaction of FOXC1 with RUNX1 on chromatin

Our RIME and immunoprecipitation (IP) data (Table S2; Figures 2D–2H) suggested a strong physical interaction of FOXC1 with RUNX1. We therefore addressed how and where these two factors co-localized with each other on chromatin. Considering first all FOXC1 and RUNX1 binding sites in Fujioka AML cells, we found 5,246 genomic locations where the absolute summit of a FOXC1 peak was 200 bp or closer to the absolute summit of a RUNX1 peak (i.e., 28.0% of FOXC1 peaks and 15.3% of RUNX1 peaks) (Figures S4A and S4B). Considering the strongest 20% of FOXC1 and RUNX1 peaks, we identified 621 sites where a strong FOXC1 peak (pileup value $\geq$ 150) was co-located with a strong RUNX1 peak (pileup value $\geq$ 200) (termed "FR-20" sites) (Figure 4E). The genome-wide coincidence of FOXC1 and CEBPA peaks (called FC-20 sites) was lower (Figure S4B; 554 sites of coincident strong FOXC1 and CEBPA binding; 26.5% of FOXC1 peaks and 13.3% of CEBPA peaks). There was virtually no genome-wide coincident strong FOXC1 and SPI1 binding (Figure S4B; FS-20 sites; 8 sites genome wide).

On the assumption that stronger peaks by pileup value were more likely to be functionally relevant (Maiques-Diaz et al., 2018), we focused our attention on an evaluation of the consequences of *FOXC1* KD at sites of strong dual FOXC1 and RUNX1 binding. Given the predominant distribution of FOXC1

Figure 2. Identification of chromatin-bound FOXC1 interacting proteins

(A) Experimental outline.

(B) Human Fujioka AML cells were infected with lentiviruses targeting the indicated genes for KD or a NTC. Bar chart shows mean + SEM CD86 mean cell fluorescence on day 5 (n = 3). Embedded panel: representative flow cytometry plots.

(C) Anti-FOXC1 immunoprecipitation (IP) in Fujioka AML cells (representative of n = 3).

(D–F) Fujioka AML cells were infected with lentiviruses expressing coding sequences for full-length or domain mutant versions of *FOXC1*. (D) FOXC1 and domain mutants used. (E and F) Western blots show expression of the indicated proteins in the indicated conditions in coimmunoprecipitation (coIP) experiments (representative of n = 3).

(G and H) 293 cells were transfected with vectors expressing coding sequences for full-length or domain mutant versions of *RUNX1b*. (G) RUNX1b and domain mutants used. (H) Western blots show expression of the indicated proteins in the indicated conditions in coIP experiments (representative of n = 2).

See also Figure S2.

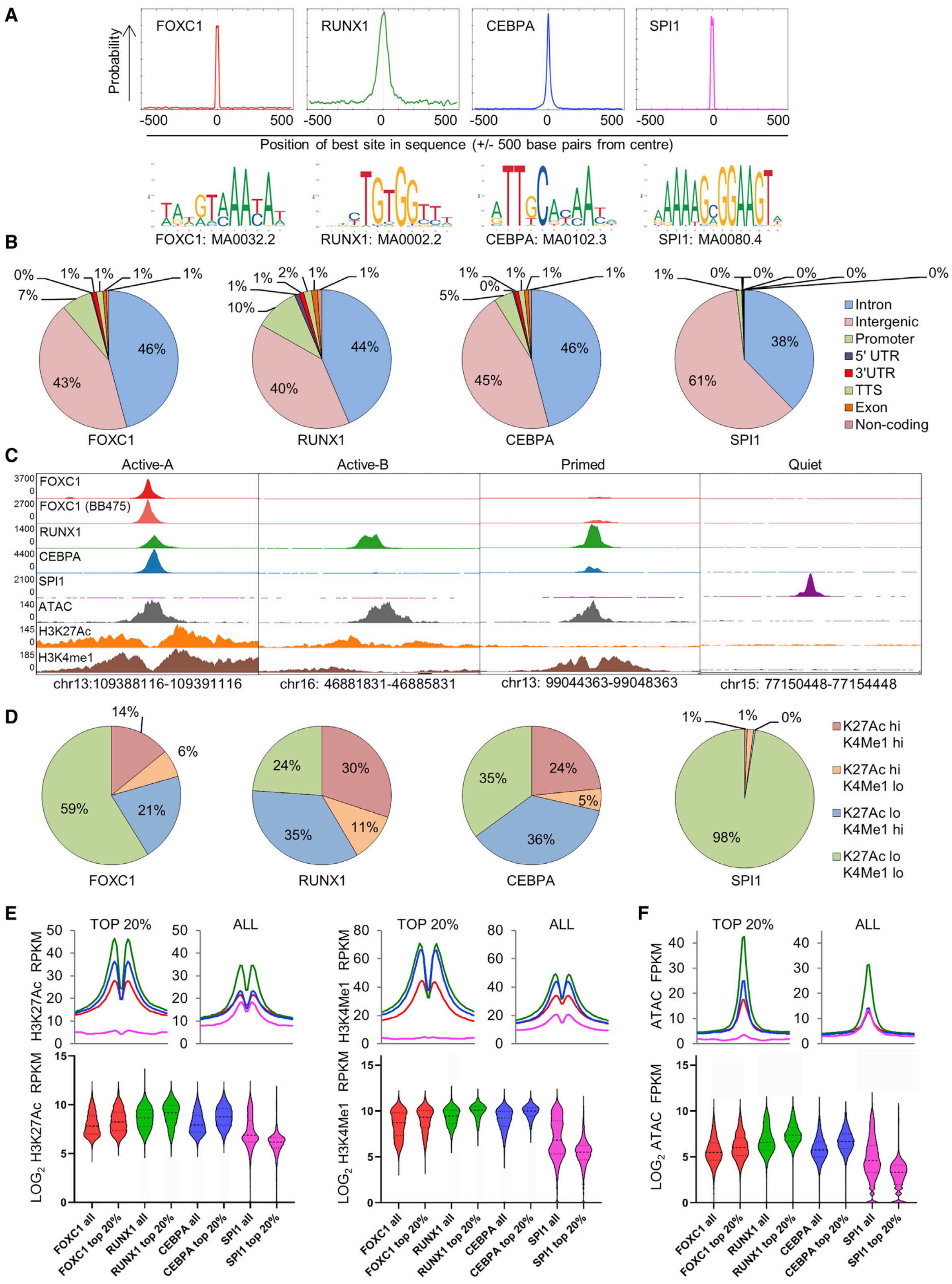

(legend on next page)

and RUNX1 peaks at putative enhancers, we excluded sites located at promoter and 5′ UTR sequences from the analysis in the first instance. There were 581 such genomic locations (which we term "FR-20 enhancer" sites). As comparators, we evaluated strong FOXC1 binding sites without a nearby strong RUNX1 peak ("F-20 enhancer" sites, n = 2,911) and vice versa for RUNX1 ("R-20 enhancer" sites, n = 5,885). Most F-20 enhancer sites were at regions of quiet chromatin, whereas the great majority of R-20 and FR-20 enhancer sites were at regions of primed or active chromatin (Figure 4F). A similar pattern was observed when all peaks were considered (Figure S4C).

Importantly, consistent with the physical interaction between FOXC1 and RUNX1 stabilizing their interaction with chromatin, we noted that at FR-20 enhancer sites, there was significantly greater FOXC1 ChIP signal by comparison with F-20 sites (mean ± SEM, 1,672 ± 97 versus 1,097 ± 19 reads/600 bp; t test, $p = 10^{-9}$; Figure 4G). Likewise, at RF-20 enhancer sites, there was significantly greater RUNX1 ChIP signal by comparison with R-20 sites (mean ± SEM, 2,076 ± 71 versus 1,675 ± 22 reads/600 bp; t test, $p = 10^{-7}$; Figure 4G). Note that FR-20 and RF-20 refer to the same set of 581 genomic locations where the summits of a strong FOXC1 and a strong RUNX1 binding peak occur within 200 bp of each other. However, for FR-20 sites, the ChIP signal shown is that surrounding the absolute summit of the FOXC1 binding peak, whereas for RF-20 sites it is that surrounding the absolute summit of the RUNX1 peak.

To determine whether a similar pattern of interaction was observed in primary patient AML blast cells, we performed ChIP-seq for FOXC1 and RUNX1 using cells from patient BB171 (Table S1). In these samples, we identified 17,539 and 21,872 peaks, respectively, and there were 8,708 genomic locations where the absolute summit of a FOXC1 peak was 200 bp or closer to the absolute summit of a RUNX1 peak. As for Fujioka cells, at FR-20 enhancer sites (n = 1,096), there was a significantly greater FOXC1 ChIP signal by comparison with F-20 sites (mean ± SEM, 1,595 ± 37 versus 1,031 ± 13 reads/600 base pairs; t test, $p = 10^{-62}$; Figures 4H and 4I). Likewise, at RF-20 enhancer sites, there was significantly greater RUNX1 ChIP signal by comparison with R-20 sites (mean ± SEM, 1,199 ± 22 versus 982 ± 9 reads/600 bp; t test, $p = 10^{-23}$; Figures 4H and 4I). A majority (324/581; 56%) of FR-20 enhancer sites in Fujioka cells overlapped with sites of colocalized FOXC1 and RUNX1 binding in BB171 AML cells; 459/1,096 (42%) sites overlapped in the opposite comparison. Thus, in both Fujioka AML cells and in primary patient AML blast cells, FOXC1 and RUNX1 associate together on chromatin with overlapping distributions.

Loss of RUNX1 and HDAC1 from FR-20 enhancer sites after FOXC1 depletion

To evaluate the consequences of FOXC1 depletion on colocalization of RUNX1, we performed *FOXC1* KD in Fujioka cells followed by ChIP-seq for RUNX1. Considering the population of 581 enhancer sites strongly co-bound by FOXC1 and RUNX1 (RF-20 enhancer sites), we observed overall a relative reduction of mean RUNX1 ChIP signal by comparison with R-20 sites (RF-20: mean ± SEM, 2,026 ± 68 [non-targeting control (NTC)] versus 1,640 ± 73 [*FOXC1* KD] reads/600 bp; t test, $p = 10^{-4}$; R-20: NTC versus KD comparison, p = not significant; Figure 4J). Interestingly, there was also a relative reduction of mean RUNX1 ChIP signal from the population of F-20 enhancer sites (F-20: mean ± SEM, 349 ± 8 [NTC] versus 227 ± 21 [*FOXC1* KD] reads/600 bp; t test, $p = 10^{-7}$; Figure 4J), suggesting that FOXC1 may stabilize RUNX1 binding to chromatin at many of these sites even where baseline RUNX1 ChIP signal is lower. We also performed ChIP-seq for CEBPA in *FOXC1* KD cells and observed that overall there was an increase in mean relative CEBPA ChIP signal at FR-20 enhancer sites following *FOXC1* KD (mean ± SEM, 1,248 ± 72 versus 1,622 ± 170 reads/600 bp; t test, p = 0.03), but no change at F-20 or R20 enhancer sites (Figure 4K).

To provide additional context for these initial analyses, as well as for subsequent analyses, we performed ChIP-seq for histone acetyltransferase EP300 and SWI/SNF chromatin remodeling complex protein SMARCC2, both of which we identified as FOXC1 interacting proteins (Table S2), histone deacetylase HDAC1, which is known to be recruited to chromatin by RUNX1, and H3K4Me2. Although HDAC2 was identified as a FOXC1 interacting protein in Fujioka AML cells, HDAC1 was identified in BB475 primary AML cells (Table S2). Our prior studies have shown HDAC1 and HDAC2 exhibit overlapping sites genome wide (data not shown). To summarize the results, there was no change in mean ChIP signal for EP300, H3K4Me2, or SMARCC2 at FR-20, F-20, or R-20 enhancer sites following *FOXC1* KD; no change in H3K27Ac ChIP signal at FR-20 and F-20 sites; and no change in ATAC-seq signal at F-20 sites (Figures 4L, 4M, and S4D–S4H). There was a modest relative increase in H3K27Ac ChIP signal at R-20 sites (mean ± SEM, 648 ± 8 versus 704 ± 9 reads/600 bp; t test, $p = 10^{-8}$; Figure 4L), as well as a modest relative decrease in ATAC-seq signal (mean ± SEM, 155 ± 2 versus 150 ± 2 reads/600 bp; t test, p = 0.03; Figure S4D). There was also a modest relative decrease in ATAC-seq signal at FR-20 sites (mean ± SEM, 170 ± 6 versus 150 ± 5 reads/600 bp; t test, p = 0.02; Figure S4D).

The most notable changes were in HDAC1 ChIP signal. There was a significant relative reduction in mean HDAC1 ChIP signal

Figure 3. Chromatin context of FOXC1, RUNX1, CEBPA, and SPI1 binding peaks
(A) MEME-ChIP motif enrichment plots.
(B) Pie charts show genome annotations for the strongest 20% of transcription factor binding peaks.
(C) Exemplar ChIP-seq tracks for chromatin categories.
(D) Pie charts show chromatin categories for strongest 20% of transcription factor binding peaks.
(E) Graphs (upper panels) show mean ChIP signal for H3K27Ac (left) or H3K4Me1 (right) ± 1 kB surrounding the indicated sets of transcription factor binding peaks. Violin plots show distribution, median (thick dotted line), and interquartile range (light dotted lines) for ChIP signal.
(F) As for (E) but for ATAC-seq signal.
FPKM, fragments per kilobase per million mapped reads. See also Figure S3.

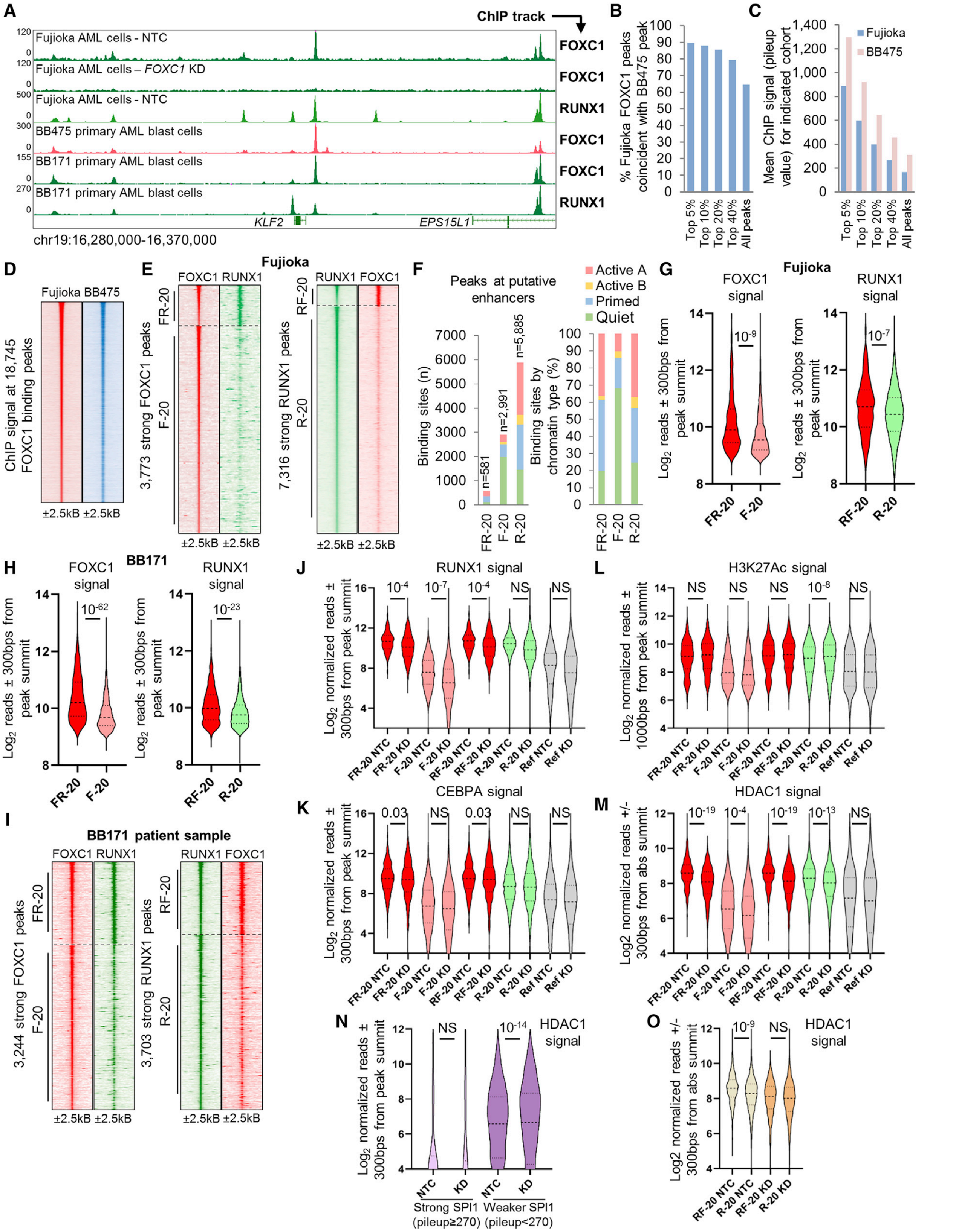

(legend on next page)

at RF-20 enhancer sites (mean ± SEM, 446 ± 12 versus 316 ± 8 reads/600 bp; t test, $p = 10^{-19}$; Figure 4M) and a smaller reduction at R-20 enhancer sites (mean ± SEM, 373 ± 6 versus 325 ± 5 reads/600 bp; t test, $p = 10^{-13}$; Figure 4M) upon *FOXC1* KD. There was also a small relative reduction in mean HDAC1 ChIP signal at F-20 sites (mean ± SEM, 144 ± 4 versus 123 ± 4 reads/600 bp; t test, $p = 10^{-4}$; Figure 4M). Balancing this was a relative increase in HDAC1 ChIP signal at lower-strength SPI1 binding sites (i.e., with pileup value < 270, equating to the lowest 60% of peaks by peak strength) (mean ± SEM, 246 ± 3 versus 281 ± 3 reads/600 bp; t test, $p = 10^{-14}$; Figure 4N); we speculate that other ETS family factors may co-occupy many of these sites.

In addition to there being a greater proportional loss of HDAC1 signal at RF-20 versus R-20 enhancer sites following FOXC1 KD (reduction of mean by 29.1% versus 12.9%), we also noted that in control cells there was significantly greater HDAC1 ChIP signal at RF-20 versus R-20 enhancer sites (mean ± SEM, 440 ± 12 versus 373 ± 6 reads/600 bp; t test, $p = 10^{-9}$; Figure 4N). In contrast, in *FOXC1* KD cells, there was no significant difference between HDAC1 ChIP signal at FR-20 versus R-20 enhancer sites (mean ± SEM, 316 ± 8 versus 325 ± 5 reads/600 bp; t test, p = not significant; Figure 4O), consistent with colocalized FOXC1 and RUNX1 stabilizing association of HDAC1 at enhancers.

In summary, these initial analyses indicate that depletion of FOXC1 leads overall to mean loss of RUNX1 and HDAC1 ChIP signal at the population of FR-20 enhancer sites, with gain of CEBPA, even though total cellular levels of RUNX1, CEBPA, and HDAC1 are largely unchanged (Figure S4I).

FOXC1 acts as a repressor at a subset of primed and active enhancers

Next, to evaluate the influence of FR-20 enhancer sites on gene expression, we performed RNA sequencing in control and *FOXC1* KD Fujioka cells and identified 9,910 expressed protein coding genes (i.e., expressed at ≥2 fragments per kilobase per million mapped reads [FPKMs] in at least one sample). After *FOXC1* KD, 349 genes were upregulated by at least 2-fold and 804 downregulated (Figure 5A). Upregulated genes included transcription factor genes with roles in monocyte/macrophage differentiation. Downregulated genes included those with roles in leukemic stem cell potential (e.g., *MYB*, *MYC*; Somervaille et al., 2009; Figure 5A). To further highlight the differentiation program induced by *FOXC1* KD in Fujioka cells, we used gene set enrichment analysis (GSEA) to compare the transcriptional changes with, as an example, those observed during phorbol ester-mediated terminal differentiation of THP1 AML cells into macrophages (Suzuki et al., 2009; Figure S5A; Table S3). There was a highly significant overlap. Of note, qPCR for key genes in primary patient AML cells (BB171) following *FOXC1* KD gave similar results (Figure S5B).

Given the overall relative loss of RUNX1 ChIP signal and gain of CEBPA ChIP signal from the 581 FR-20 enhancer sites (Figures 4I and 4J), we evaluated the proportional change in ChIP signal for both these factors at each enhancer. FR-20 enhancer sites exhibited four patterns of change in RUNX1 and CEBPA ChIP signal following *FOXC1* KD according to whether relative RUNX1 and CEBPA ChIP signal increased or decreased (Figure 5B). We used Genomic Regions Enrichment of Annotations Tool (GREAT) (McLean et al., 2010) to map genomic coordinates to the basal regulatory regions of nearby genes and then performed GSEA (Table S3). Within the limitations of this approach (regulatory elements do not necessarily control expression of the closest genes), we nevertheless observed strong enrichment of genes positioned close to group 1 enhancers (reduced RUNX1 signal, increased CEBPA signal; $RUNX1^{down}$ $CEBPA^{up}$; Figures 5B and 5C; Table S3) among those upregulated following *FOXC1* KD. This contrasted with GSEA for the other three groups (Figure 5C) where no significant enrichment was observed among either upregulated or downregulated genes. Leading edge analysis revealed that the enrichment of group 1 genes was driven by those upregulated during normal monocytic lineage expression, such as *KLF2*, *MBD2*, *ID1*, *S100A12*, and *RGS2*, among others (Figure S5C; Bagger et al., 2019). Eighty-eight percent of group 1 FR-20 enhancers exhibited primed or active chromatin configuration in basal conditions (Figure S5D). Of note, 122/224 (54%) of group 1 enhancers were also co-occupied by FOXC1 and RUNX1 in primary AML cells (BB171) with strong binding close to genes such as *KLF2*, *ID1*, and *MEF2C* (Figures 4A and S5E).

Group 1 ($RUNX1^{down}$ $CEBPA^{up}$) enhancers exhibited significantly greater baseline RUNX1 binding compared with group 2 ($RUNX1^{down}$ $CEBPA^{down}$) and 4 ($RUNX1^{up}$ $CEBPA^{down}$) enhancers, and intermediate levels of CEBPA binding compared with group 2 (lower) and group 4 (higher). There was no change

Figure 4. FOXC1 chromatin binding in AML cells and colocalization with RUNX1

(A) Exemplar ChIP-seq tracks.

(B) Bar chart shows percentage of FOXC1 binding peaks in Fujioka AML cells in the indicated peak cohorts, which are coincident with a FOXC1 binding peak in BB475 primary AML cells.

(C) Bar chart shows mean FOXC1 ChIP signal in the indicated peak cohorts in Fujioka cells and at the same genomic locations in BB475 primary AML cells.

(D) Heatmaps show FOXC1 ChIP signal at FOXC1 binding sites in Fujioka and at the same genomic locations in BB475 primary AML cells, ranked by peak strength.

(E and I) Heatmaps show ChIP signal for RUNX1 at strong FOXC1 binding sites (left panel) and FOXC1 at strong RUNX1 binding sites (right panel) in Fujioka cells (E) or BB171 primary patient AML blast cells (I).

(F) Bar charts show chromatin categories for the indicated classes of strong FOXC1 and RUNX1 binding peaks in Fujioka cells by number (left panel) and proportion (right panel).

(G, H, and J–O) Violin plots show distribution, median (thick dotted line), and interquartile range (light dotted lines) for ChIP signal for the indicated proteins at sites with strong FOXC1 and RUNX1 binding (FR-20, FOXC1 centered; RF-20 RUNX1 centered), strong FOXC1 binding (F-20), or strong RUNX1 binding (R-20) in Fujioka AML cells (G, J–M, and O), BB171 primary patient AML blast cells (H), or SPI1 binding sites (N) for, where indicated, control cells (NTC) or following *FOXC1* KD.

NS, not significant; Ref, reference cohort used for normalization between experiments. p values, unpaired t test. See also Figure S4.

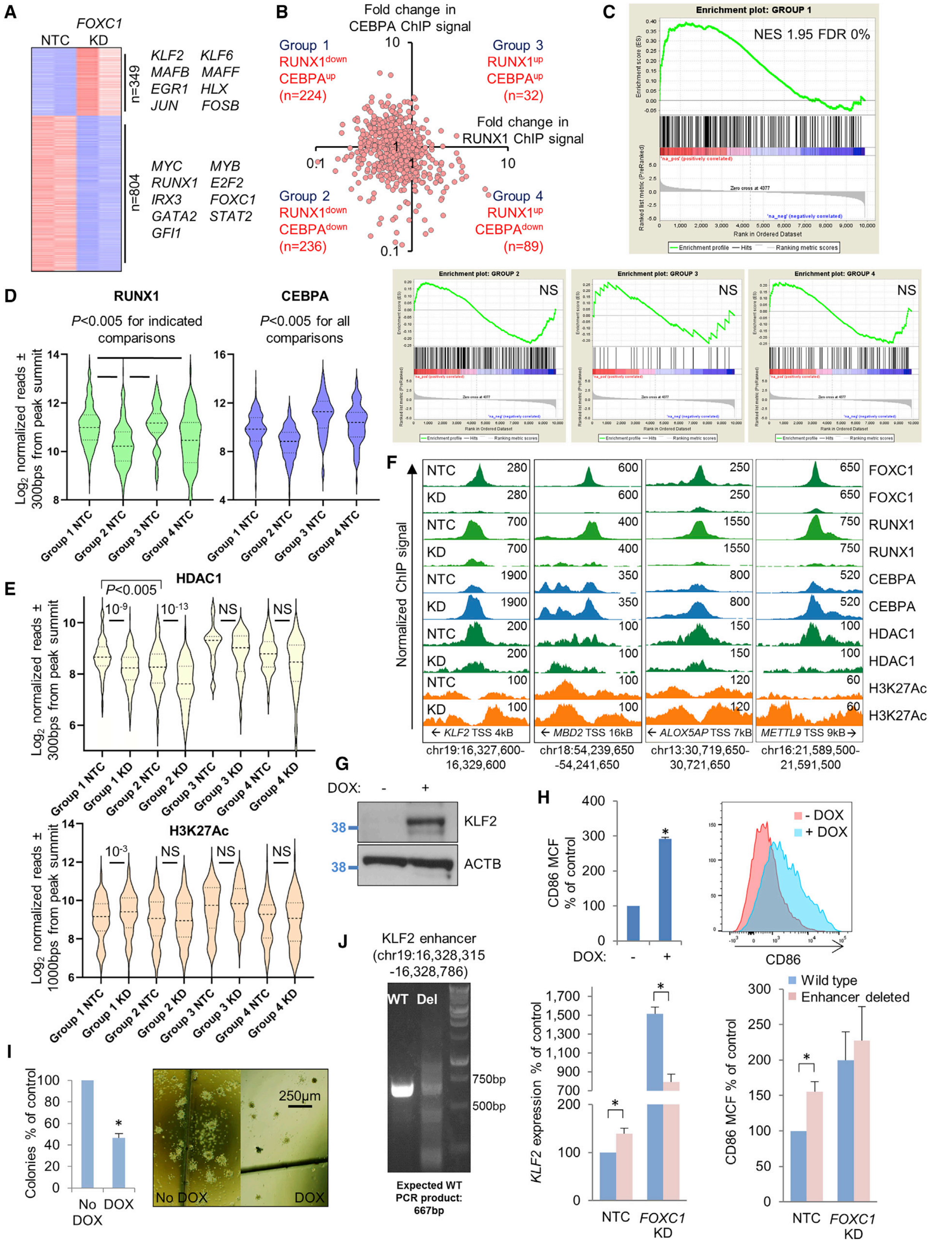

(legend on next page)

in H3K4Me2, SMARCC2, or EP300 ChIP signal, or ATAC-seq signal, for any of the enhancer groupings following *FOXC1* KD (data not shown). However, there was a significant reduction in HDAC1 ChIP signal at group 1 and group 2 enhancers (i.e., those where there was a reduction in RUNX1 signal) and a significant relative increase in H3K27Ac signal at group 1 sites (Figures 5E and 5F). As for RUNX1, there was significantly greater ChIP signal for HDAC1 at group 1 versus group 2 enhancers.

One of the group 1 FR-20 enhancers was positioned 4 kB downstream of the transcription start site for *KLF2*, a gene involved in monocytic lineage differentiation and upregulated following *FOXC1* KD. To confirm the ability of *KLF2* to promote differentiation of AML cells, we induced its expression in Fujioka cells (Figure 5G) and noted both upregulation of CD86 (Figure 5H) and reduction of clonogenic activity, with both fewer and smaller colonies in the presence of increased KLF2 expression (Figure 5I). Consistent with this FOXC1/RUNX1-bound regulatory element serving as a *KLF2* repressor in steady state, its CRISPR-mediated deletion induced a 40% increase in *KLF2* expression and upregulation of CD86 (Figure 5J). Interestingly, following *FOXC1* KD in enhancer-deleted cells, upregulation of *KLF2* was lower than in the control cells even though CD86 upregulation was similar, consistent with this element being required for maximal upregulation of *KLF2* expression during a differentiation process controlled by multiple genes and regulatory elements operating in parallel.

Thus, at a discrete set of regulatory elements distributed close to genes upregulated during myelomonocytic differentiation, and which exhibit high RUNX1 and intermediate CEBPA binding (i.e., group 1 FR-20 enhancers), FOXC1 serves as a transcription repressor through stabilizing RUNX1 and HDAC1 binding, thus limiting enhancer activity.

To further confirm the significance of the physical interaction of FOXC1 with RUNX1 in conferring a differentiation block in *FOXC1*^{high} AML cells, we generated a FOXC1 Forkhead domain-RUNX1 fusion protein (FKD-RUNX1) (Figure S6A). In the presence of induced FKD-RUNX1 in Fujioka cells, *FOXC1* KD failed to promote immunophenotypic differentiation (Figure S6B). In contrast, induced expression in Fujioka cells of FOXC1 mutants G165R and F112S (Figure S6C), which exhibit reduced interaction with RUNX1 (Figure 2F), promoted immunophenotypic differentiation (Figure S6D). We performed qPCR in Fujioka cells expressing the FOXC1 G165R mutant and observed similar gene expression changes to those observed in Fujioka cells following *FOXC1* KD (Figure S6E).

FOXC1 KD triggers redistribution of RUNX1 from enhancers to promoters

Evaluation of RUNX1 ChIP signal at FR-20 enhancer sites (Figure 5B) hinted at a redistribution of RUNX1 binding following *FOXC1* KD. MACS2 identified 17,589 RUNX1 binding peaks in FOXC1 KD Fujioka cells. Although the bulk of called peaks in the KD condition overlapped with peaks in the control condition (Figure S6F; i.e., absolute peak summits within 200 bp of each other), when the strongest 20.1% of RUNX1 peaks in KD cells (pileup value $\geq$ 168) were considered, only 37.2% overlapped with a strong RUNX1 peak in control cells (Figure 6A). We grouped strong RUNX1 peaks into three categories as shown in Figure 6A. Group A strong RUNX1 peaks (control cells only) were predominantly enhancer bound, with only 9% bound to 5′ UTR or promoter regions. By contrast, 58% of group C strong RUNX1 peaks (*FOXC1* KD cells only) were 5′ UTR or promoter bound (Figure 6B). The shared set of group B strong RUNX1 peaks (found in both control and *FOXC1* KD conditions) exhibited an intermediate pattern. There was a significant relative decrease in ChIP signal at group A peaks for RUNX1 (mean $\pm$ SEM, 1,425 $\pm$ 10 versus 1,056 $\pm$ 50 reads/600 bp; t test, $p = 10^{-13}$) and HDAC1 (mean $\pm$ SEM, 338 $\pm$ 4 versus 290 $\pm$ 3 reads/600 bp; t test, $p = 10^{-23}$), and a significant increase in CEBPA (mean $\pm$ SEM, 707 $\pm$ 18 versus 806 $\pm$ 27 reads/600 bp; t test, $p = 10^{-3}$) and H3K27Ac (mean $\pm$ SEM, 635 $\pm$ 6 versus 679 $\pm$ 7 reads/600 bp; t test, $p = 10^{-6}$) ChIP signal (Figures 6C–6F). Group C peaks displayed the opposite pattern: there was a significant relative increase in ChIP signal for RUNX1 (mean $\pm$ SEM, 543 $\pm$ 10 versus 2,523 $\pm$ 52 reads/600 bp; t test, $p < 10^{-50}$) and HDAC1 (mean $\pm$ SEM, 1,649 $\pm$ 26 versus 1,871 $\pm$ 28 reads/600 bp; t test, $p = 10^{-9}$), and a significant decrease in CEBPA (mean $\pm$ SEM, 1,220 $\pm$ 53 versus 821 $\pm$ 43 reads/600 bp; t test, $p = 10^{-9}$) and H3K27Ac (mean $\pm$ SEM, 975 $\pm$ 14 versus 896 $\pm$ 13 reads/600 bp; t test, $p = 10^{-5}$) ChIP signal (Figures 6C–6F). Group B peaks displayed an intermediate pattern between groups A and C. We confirmed the decrease and increase in RUNX1 ChIP signal by ChIP PCR for a number of group A and group C peaks (Figures 6G and S6G).

MEME-ChIP confirmed that genomic sequences at the center of group A and group B RUNX1 binding peaks were strongly enriched for RUNX1 motifs (Figure 6H). By contrast, there was no enrichment for RUNX1 motifs in genomic sequences surrounding group C RUNX1 peaks (found in *FOXC1* KD cells only). Rather, there was enrichment for several short ungapped GC-rich motifs identified by Discriminative Regular Expression Motif

Figure 5. Reduced RUNX1 and increased CEBPA ChIP signal at enhancers controlling differentiation genes after *FOXC1* KD

(A–F) Human Fujioka AML cells were infected with a lentivirus targeting *FOXC1* for KD or an NTC. (A) Heatmap shows differentially expressed genes on day 4 after KD initiation; transcription factor genes are highlighted. (B) Dot plot shows fold change in relative ChIP signal at 581 FR-20 enhancer sites and definition of four sub-groups. (C) GSEA plots. (D and E) Violin plots show distribution, median (thick dotted line), and interquartile range (light dotted lines) for ChIP signal for the indicated proteins and the indicated groups of FR-20 enhancer sites in control (NTC) or *FOXC1* KD cells on day 5. p value, one-way ANOVA with Tukey post hoc or unpaired t test. (F) Exemplar ChIP-seq tracks.

(G–I) Fujioka cells were infected with lentiviruses expressing *KLF2* under the control of a doxycycline-regulated promoter. (G) Western blot shows induced expression of KLF2. (H) Bar chart (left panel) shows mean + SEM mean cell fluorescence (MCF) for CD86 (n = 3). Right panel: representative flow cytometry plots. (I) Bar chart (left panel) shows means + SEM CFC frequencies of *KLF2*-expressing Fujioka cells relative to control cells after 10 days in semi-solid culture (n = 3). Right panel: representative colonies.

(J) CRISPR deletion of a *KLF2* regulatory element (left panel) and bar charts showing mean + SEM *KLF2* expression relative to *ACTB* as determined by qPCR (middle panel) and CD86 cell fluorescence (right panel) on day 4 after KD initiation (n = 3; *p < 0.05 by unpaired t test).

FDR, false discovery rate; NES, normalized enrichment score. See also Figure S5.

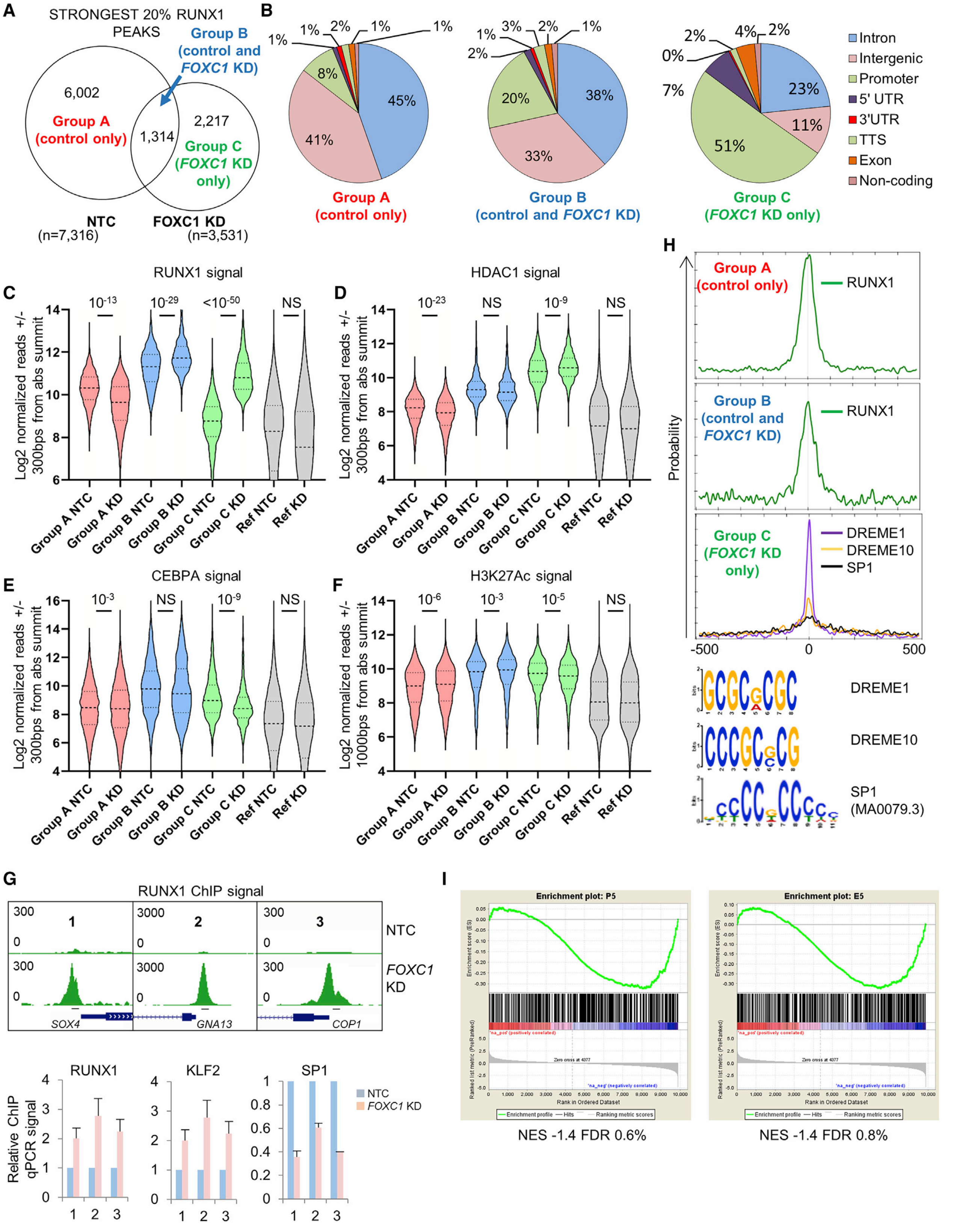

(legend on next page)

Elicitation (DREME), as well as for SP1, a member of the SP1/Kruppel-like family of transcription factors. Interestingly, using ChIP PCR at three exemplar group C promoters of genes whose expression was downregulated, in addition to an increase in RUNX1 ChIP signal, we observed an increase in KLF2 ChIP signal and a concomitant decrease in SP1 ChIP signal (Figure 6G). We speculated that KLF2 might interact with RUNX1, recruiting it to group C promoters through KLF2's interaction with GC-rich sequences (Figure S6H), in the process displacing related factors with transcription activating potential.

In the same way that loss of RUNX1 from group 1 FR-20 enhancers was associated with an increase in expression of nearby genes (Figure 5C), consistent with RUNX1's repressor activity, gain of RUNX1 at gene promoters was associated with downregulated expression. Following *FOXC1* KD using GSEA, we observed strong enrichment among downregulated genes of those whose promoters or nearby enhancers were subject to the strongest accumulation of RUNX1 (Figure 6I).

Together these data demonstrate that *FOXC1* KD triggers a differentiation process that involves the redistribution of the transcription repressive activity of RUNX1 from enhancers to promoters.

Enhanced recruitment of Groucho repressor TLE3 to RF-20 enhancer sites

Among the set of high-confidence FOXC1 interacting proteins (Table S2), we identified the Groucho co-repressor family member TLE3. TLE proteins bind via their WD40 β-propeller domain to a range of transcription factors via either a C-terminal WRPW/Y motif or an internal Engrailed homology motif (FxIxxIL) to confer transcription repression through mechanisms that remain incompletely understood (Jennings and Ish-Horowicz, 2008). *Drosophila* Runt and Groucho interact genetically, and the interaction can be mapped to a C-terminal VWRPY sequence present in all RUNX proteins (Aronson et al., 1997). We also noted that the Eukaryotic Linear Motif (ELM) resource (Kumar et al., 2020) predicted a putative Engrailed homology motif in the inhibitory domain of FOXC1 (GFSVDNIMT; amino acids 307–315).

Immunoprecipitation of endogenous FOXC1 or RUNX1 in Fujioka cells confirmed physical interactions with TLE3 (Figures 7A and 7B) and, consistent with the concept that TLE3 is a critical contributor to the myeloid differentiation block, *TLE3* KD induced immunophenotypic and morphological differentiation and loss of clonogenic potential (Figures 7C–7F). We observed similar findings in primary patient AML cells (Figures 7G and 7H). We performed ChIP-seq for TLE3 and observed a strong positive correlation genome wide of ChIP signal for TLE3 and RUNX1 (Figures 7I and S7A). Although there was no evidence in genome-wide analysis that FOXC1 alone was able to recruit TLE3 to chromatin (Figure S7B), there was nevertheless significantly more TLE3 ChIP signal at RF-20 enhancer sites by comparison with R-20 enhancer sites (mean ± SEM, 2,110 ± 123 versus 1,653 ± 26 reads/600 bp; t test, $p = 10^{-4}$) (Figures 7I and 7J). This indicates that FOXC1 enhances or stabilizes the recruitment of the Groucho repressor TLE3 to chromatin by RUNX1. Following *FOXC1* KD, there was a significant loss of TLE3 ChIP signal from group 1 ($RUNX1^{down}$ $CEBPA^{up}$) and group 2 ($RUNX1^{down}$ $CEBPA^{down}$) FR-20 enhancers (Figure 7K); indeed, the fold changes in RUNX1 and TLE3 ChIP signal at FR-20 enhancer sites following FOXC1 KD were highly correlated (Figure 7L). In the same way in which RUNX1 ChIP signal was redistributed from enhancers to promoters following *FOXC1* KD, so too was TLE3 ChIP signal (Figures 7M and 7N), with a similar pattern of motif enrichment (Figure S7C). As for the RUNX1 redistribution, the bulk of all called TLE3 peaks was identified in both control and *FOXC1* KD cells (Figure S7D), and cellular TLE3 protein levels were unchanged (Figure S7E). Thus, FOXC1 stabilizes association of RUNX1, HDAC1, and the Groucho family repressor protein TLE3 at a discrete set of enhancers to limit locoregional transcription. Depletion of FOXC1 triggers a redistribution of RUNX1, TLE3, and HDAC1 to promoters of critical growth and renewal genes, including, for example, *MYB* (Figure 7N) and *IRS2* (Figure S7F).

DISCUSSION

Although AML as a pathologic entity is marked by genetic heterogeneity, it is defined by a block to myeloid differentiation. Expression of *FOXC1*, which is not present in normal hematopoietic lineages, contributes to blocked myeloid differentiation in molecular subtypes of AML with concomitant high *HOX* gene expression (Somerville et al., 2015). Although the mechanisms by which *FOXC1* is de-repressed in a lineage-inappropriate manner remain to be determined, it is of note that, in addition to its defined genetic lesions, AML is also marked by widespread epigenetic changes, for example, in DNA methylation (Cancer Genome Atlas Research Network, 2013) or histone modifications. Genetic lesions may confer epigenetic plasticity, and thus be permissive for outgrowth of clones driven or sustained by lineage-inappropriate transcriptional networks. Such networks would be attractive targets for therapy, given their lack of expression or importance in normal hematopoietic cells.

Our studies in primary patient and Fujioka cells reveal the set of high-confidence protein interactions made by FOXC1 in

Figure 6. *FOXC1* knockdown triggers redistribution of RUNX1 binding
(A) Venn diagram shows intersection of the strongest 20% of RUNX1 binding peaks in control (NTC) or *FOXC1* KD Fujioka AML cells and classification of groups.
(B) Pie charts show genome annotations for RUNX1 binding peaks in groups A–C.
(C–F) Violin plots show distribution, median (thick dotted line), and interquartile range (light dotted lines) for ChIP signal for the indicated proteins and groups in control (NTC) or *FOXC1* KD Fujioka AML cells. p values, unpaired t test.
(G) Exemplar ChIP-seq tracks (upper panel) with confirmatory ChIP-PCR (lower panels; n = 3).
(H) MEME-ChIP and DREME motif enrichment plots for the indicated groups.
(I) GSEA plots.
E5, enhancers that exhibit ≥5-fold increase in RUNX1 ChIP signal; P5, genes whose promoters exhibit ≥5-fold increase in RUNX1 ChIP signal. See also Figure S6.

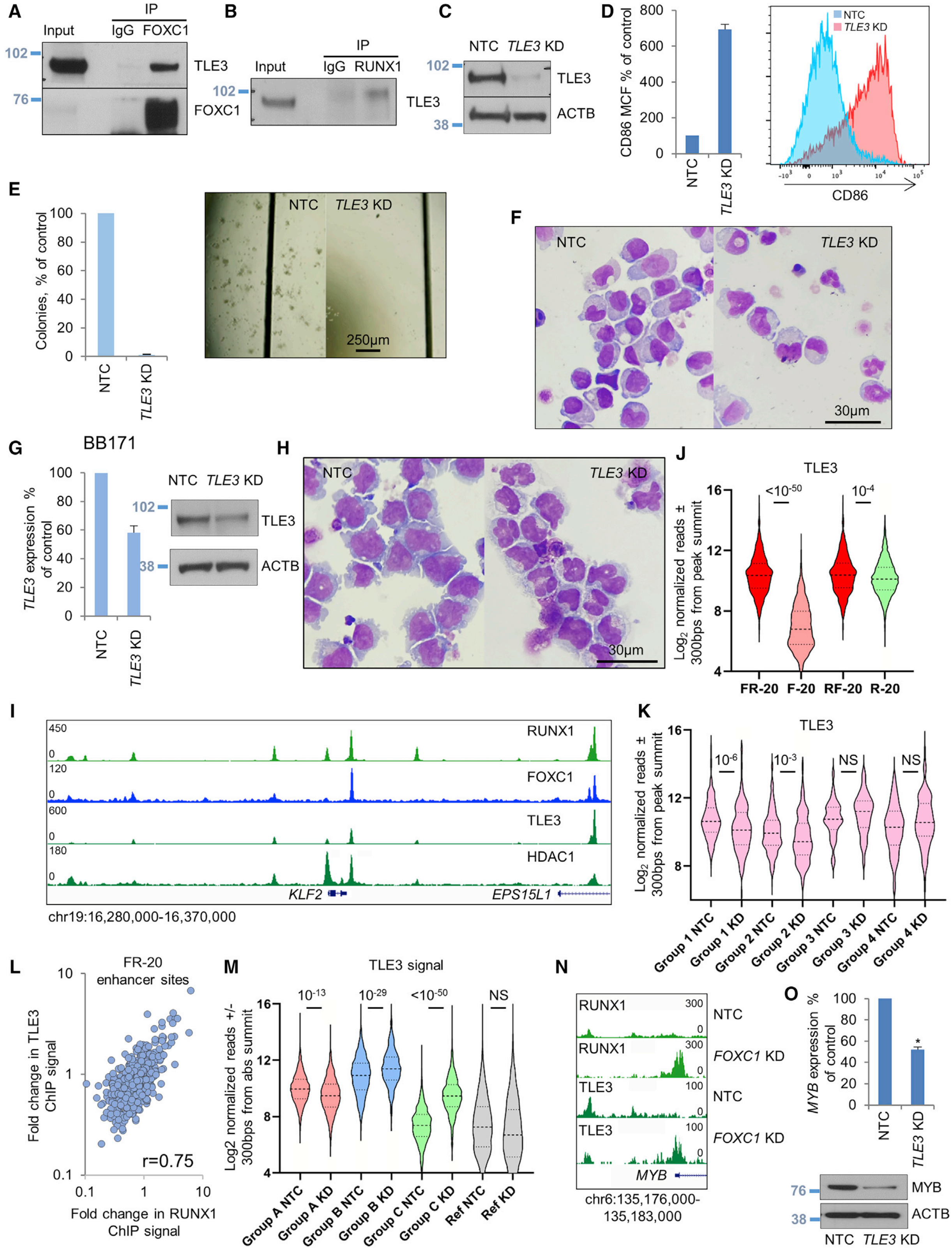

(legend on next page)

AML and highlight in particular its interactions with proteins with critical roles in AML biology: RUNX1 and CEBPA. The ability of FOXC1 to bind to and interfere with these factors suggests an unexpected, additional mechanism in myeloid leukemogenesis. RUNX1 is a master regulator of developmental hematopoiesis, controlling the emergence of hematopoietic stem cells from hemogenic endothelium (Lancrin et al., 2009); in adulthood it is required for proper megakaryocyte and lymphoid development and suppression of a myeloproliferative phenotype (Growney et al., 2005). Genetic lesions of *RUNX1* in AML, whether by somatic mutation or chromosomal translocation, are frequent, although the mechanisms by which they promote leukemic transformation are incompletely understood. RUNX1-RUNX1T1 (AML1-ETO) recruits a multitude of co-repressors to its binding sites, whereas somatic mutations of *RUNX1*, which often target sequences coding for the Runt Homology Domain, are inactivating or confer dominant-negative activity (Sood et al.,2017). Functionally, RUNX1 may be sequestered away from chromatin by CBFB-SMMHC or have its activity modified by interaction with CBFB-SMMHC, which also recruits co-repressors to sites of RUNX1 binding (Beghini, 2019). Biallelic mutations in *CEBPA*, which block CEBP factor homo- or heterodimerization, or DNA binding, are also frequent in AML (Wilhelmson and Porse, 2020).

We find that the Forkhead domain of FOXC1 interacts with RUNX1, and together these factors co-occupy hundreds of primed and active enhancers, including many of which are distributed close to genes upregulated in monocyte/macrophage differentiation. Sites of strong co-localized FOXC1 and RUNX1 binding exhibit higher levels of RUNX1, TLE3, and HDAC1 binding by comparison with strong RUNX1 binding sites that do not have co-localized FOXC1. In our studies, FOXC1 and RUNX1 serve as transcription repressors: in the genome-wide redistribution of RUNX1 binding, which follows *FOXC1* KD, loss of RUNX1 from enhancers associates with increased expression of nearby genes, whereas the opposite is the case for both enhancers and promoters that gain RUNX1. This is further emphasized by our observation of an extremely strong genome-wide correlation in both control and *FOXC1* KD cells of binding peaks for RUNX1, HDAC1, and the Groucho repressor TLE3. Because RUNX1 is redistributed with differentiation, so too is HDAC1 and TLE3. The Groucho/TLE family of corepressors interacts with at least five families of transcription factor and plays critical roles in development. The mechanisms by which Groucho family proteins confer transcription repression are poorly understood but may include reduction of chromatin accessibility and recruitment of deacetylase activity (Jennings and Ish-Horowicz, 2008). TLE3 has not previously been reported to have a role in AML, although Groucho homologs TLE1 and TLE4 have been suggested to restrain Kasumi-1 AML cell growth (Dayyani et al., 2008).

Our studies highlight the challenges associated with determining which among many thousands of genome-wide transcription factor binding sites are functionally the most important and the complexities of enhancer biology; it cannot be presumed that each binding site has equal biological significance. It is notable that those FOXC1 sites controlling expression of differentiation genes following *FOXC1* KD (group 1 enhancers; Figure 5B) account for fewer than 1.5% of the total. These sites were generally marked by primed or active histone modifications, accessible chromatin, strong RUNX1 and FOXC1 binding, and intermediate levels of CEBPA binding. The consequences of FOXC1 depletion at any one RUNX1-bound enhancer were nevertheless variable and likely dependent upon the presence or absence of many dozens of additional co-located factors. Presumably the inappropriate occupation of this discrete subset of primed and active enhancers by FOXC1 inhibits their normal activity by preventing, through RUNX1/TLE3/HDAC1 recruitment, the normal upregulation of critical genes required for differentiation. Consistent with FOXC1 having pioneer activity, we also observed widespread and strong binding at sites of quiet chromatin but found no evidence that cellular depletion of FOXC1 at these sites contributed acutely to cellular differentiation.

RUNX1 and CEBPA have been shown to interact through the C-terminal basic leucine zipper domain of CEBPA and the Runt domain of RUNX1 (Zhang et al., 1996), and at many FOXC1/RUNX1 co-occupied enhancers in our study, there was significant co-localized CEBPA binding. Nevertheless, overall, we noted that relative loss of RUNX1 from genomic binding sites correlated with gain of CEBPA. Following *FOXC1* KD, CEBPA was recruited to enhancers near to upregulated myeloid differentiation genes as RUNX1 was lost, and vice versa at enhancers and promoters close to downregulated genes. We speculate that obstruction of RUNX1 and CEBPA transcription factor switching at enhancers and promoters may contribute to myeloid differentiation blockade, a

Figure 7. FOXC1 stabilizes TLE3 and RUNX1 binding at enhancers controlling differentiation

(A and B) Western blots (representative of n = 3) show the indicated IPs from Fujioka cell lysates.

(C–F) Fujioka AML cells were infected with a lentivirus targeting *TLE3* for KD or an NTC. (C) Western blot. (D) Bar chart (left panel) shows mean + SEM CD86 mean cell fluorescence (n = 3). Right panel: representative flow cytometry plots. (E) Bar chart (left panel) shows the mean + SEM CFC frequencies for *TLE3* KD cells relative to control cells enumerated after 12 days (n = 3). Right panel: representative colonies. (F) Cytospins from (D).

(G and H) Primary patient AML cells (BB171) were infected with lentiviral vectors targeting *TLE3* for KD or an NTC. (G) Transcript and protein KD. (H) Cytospins from day 12 after KD initiation.

(I) Exemplar ChIP-seq tracks.

(J) Violin plot shows distribution, median (thick dotted line), and interquartile range (light dotted lines) for TLE3 ChIP signal at the indicated sites in control (NTC) Fujioka AML cells. p value, unpaired t test.

(K) Violin plot shows TLE3 ChIP signal at the indicated FR-20 enhancer sites in control and *FOXC1* KD Fujioka cells. p values, unpaired t test.

(L) Dot plot shows fold change in relative TLE3 and RUNX1 ChIP signal at each of 581 FR-20 enhancer sites.

(M) Violin plot shows TLE3 ChIP signal at the indicated RUNX1 group binding sites in control and *FOXC1* KD Fujioka cells. p values, unpaired t test.

(N) Exemplar ChIP-seq tracks.

(O) Bar chart (top panel) shows mean + SEM *MYB* expression relative to *ACTB* (qPCR). Bottom panel: western blot.

See also Figure S7.

concept supported by the finding that targeting RUNX1 to sites of FOXC1 binding using a FOXC1 FKD-RUNX1c fusion blocked up-regulation of differentiation genes.

The differentiation-associated redistribution of RUNX1 binding, which has also been observed following KD of *RUNX1-RUNX1T1* in Kasumi-1 cells (Ptasinska et al., 2012), is to short ungapped GC-rich DNA motifs rather than RUNX1 motifs. This suggests that the RUNX1/TLE3/HDAC1 repressor complex is likely recruited to these sites by another factor, for example, a Kruppel-like family transcription factor such as KLF2 or KLF4. It remains unclear whether additional mechanisms are involved in this switch, including post-translational modifications of RUNX1, for example.

Finally, our work suggests targets for therapeutic intervention and provides insights that may enhance understanding of *FOXC1*^{high} solid malignancies and the Axenfeld-Rieger syndrome. Compounds that target the interaction of the Forkhead domain of FOXC1 with RUNX1 would be predicted to destabilize enhancer-bound RUNX1/TLE3/HDAC1 to promote differentiation and may be beneficial in *FOXC1*^{high} *HOX*^{high} AMLs. Further, our studies of *TLE3* KD in primary patient AML cells suggest that compounds that target the interaction of the C-terminal VWRPY domain of RUNX1 with the WD40 β-propeller domain of TLE3, or the domains by which TLE3 undergoes oligomerization, may also be beneficial.

STAR★METHODS

Detailed methods are provided in the online version of this paper and include the following:

- KEY RESOURCES TABLE
- RESOURCE AVAILABILITY
  - Lead contact
  - Materials availability
  - Data and code availability
- EXPERIMENTAL MODEL AND SUBJECT DETAILS
  - Cell lines
  - Human tissue and ethical approvals
- METHOD DETAILS
  - Antibodies
  - FOXC1 antibody production
  - Flow cytometry, protocols and antibodies
  - Protein extraction, western blotting and IP
  - Mass Spectrometry
  - Expression constructs and vectors
  - Viral particle manufacture
  - RNA preparation and quantitative PCR
  - KLF2 enhancer deletion
  - RNA sequencing
  - Chromatin immunoprecipitation and next generation sequencing
  - ChIP PCR
  - ATAC sequencing
- QUANTIFICATION AND STATISTICAL ANALYSIS
  - Gene set enrichment analysis
  - ChIP sequencing normalization between experiments
  - Statistics

SUPPLEMENTAL INFORMATION

Supplemental information can be found online at https://doi.org/10.1016/j.celrep.2021.109725.

ACKNOWLEDGMENTS

This work was supported by Cancer Research UK grant numbers C5759/A20971, A27412 (to F.S., F.M.R.A., F.C., I.R.-C., G.J.S., T.C.P.S., M.L.-A.-L., and G.L.) and A20411 (to E.K.P. and J.S.C.); a Manchester-Melbourne GOLDEN PhD studentship (to O.S.); a Kay Kendall Leukaemia Fund Junior Research Fellowship (KKL1185) (to I.R.-C.); a Blood Cancer UK clinician scientist award (to D.H.W.); The Oglesby Charitable Trust (to D.H.W.); and The Christie Charity. J.S.C. acknowledges support from the University of Cambridge, Cancer Research UK, and Hutchison Whampoa Ltd. We are grateful to Mike Green, Jeff Barry, Wolfgang Breitwieser, Gillian Newton, Amy Priestman, John Weightman, and the Proteomics Facility at Cancer Research UK Cambridge Institute for technical support.

AUTHOR CONTRIBUTIONS

F.S., I.R.-C., F.C., G.J.S., and O.J.S. performed experiments; M.L.-A.-L. and G.L. generated RUNX1 mutant constructs; F.S., F.M.R.A., and T.C.P.S. performed bioinformatics analyses; F.S., E.K.P., and J.S.C. performed RIME; D.H.W. performed genotyping of Biobank samples; F.S. and T.C.P.S. wrote the manuscript; all authors reviewed the final version.

DECLARATION OF INTERESTS

The authors declare no competing interests.

Received: February 3, 2021
Revised: July 13, 2021
Accepted: August 26, 2021
Published: September 21, 2021

REFERENCES

Andrews, S. (2010). FastQC. Babraham Bioinformatics. https://www.bioinformatics.babraham.ac.uk/projects/fastqc/.

Aronson, B.D., Fisher, A.L., Blechman, K., Caudy, M., and Gergen, J.P. (1997). Groucho-dependent and -independent repression activities of Runt domain proteins. Mol. Cell. Biol. *17*, 5581–5587.

Assi, S.A., Imperato, M.R., Coleman, D.J.L., Pickin, A., Potluri, S., Ptasinska, A., Chin, P.S., Blair, H., Cauchy, P., James, S.R., et al. (2019). Subtype-specific regulatory network rewiring in acute myeloid leukemia. Nat. Genet. *51*, 151–162.

Bagger, F.O., Kinalis, S., and Rapin, N. (2019). BloodSpot: a database of healthy and malignant haematopoiesis updated with purified and single cell mRNA sequencing profiles. Nucleic Acids Res. *47* (*D1*), D881–D885.

Beghini, A. (2019). Core Binding Factor Leukemia: Chromatin Remodeling Moves Towards Oncogenic Transcription. Cancers (Basel) *11*, 1973.

Buenrostro, J.D., Giresi, P.G., Zaba, L.C., Chang, H.Y., and Greenleaf, W.J. (2013). Transposition of native chromatin for fast and sensitive epigenomic profiling of open chromatin, DNA-binding proteins and nucleosome position. Nat. Methods *10*, 1213–1218.

Cancer Genome Atlas Research Network; Weinstein, J.N., Collisson, E.A., Mills, G.B., Shaw, K.R., Ozenberger, B.A., Ellrott, K., Shmulevich, I., Sander, C., and Stuart, J.M. (2013). The Cancer Genome Atlas Pan-Cancer analysis project. Nat. Genet. *45*, 1113–1120.

Challen, G.A., and Goodell, M.A. (2020). Clonal hematopoiesis: mechanisms driving dominance of stem cell clones. Blood *136*, 1590–1598.

Dayyani, F., Wang, J., Yeh, J.R., Ahn, E.Y., Tobey, E., Zhang, D.E., Bernstein, I.D., Peterson, R.T., and Sweetser, D.A. (2008). Loss of TLE1 and TLE4 from

CellPress
OPEN ACCESS

the del(9q) commonly deleted region in AML cooperates with AML1-ETO to affect myeloid cell proliferation and survival. Blood *111*, 4338–4347.

Dobin, A., Davis, C.A., Schlesinger, F., Drenkow, J., Zaleski, C., Jha, S., Batut, P., Chaisson, M., and Gingeras, T.R. (2013). STAR: ultrafast universal RNA-seq aligner. Bioinformatics *29*, 15–21.

Gilding, L.N., and Somervaille, T.C.P. (2019). The Diverse Consequences of *FOXC1* Deregulation in Cancer. Cancers (Basel) *11*, 184.

Glont, S.E., Chernukhin, I., and Carroll, J.S. (2019). Comprehensive Genomic Analysis Reveals that the Pioneering Function of FOXA1 Is Independent of Hormonal Signaling. Cell Rep. *26*, 2558–2565.e3.

Growney, J.D., Shigematsu, H., Li, Z., Lee, B.H., Adelsperger, J., Rowan, R., Curley, D.P., Kutok, J.L., Akashi, K., Williams, I.R., et al. (2005). Loss of Runx1 perturbs adult hematopoiesis and is associated with a myeloproliferative phenotype. Blood *106*, 494–504.

Harris, W.J., Huang, X., Lynch, J.T., Spencer, G.J., Hitchin, J.R., Li, Y., Ciceri, F., Blaser, J.G., Greystoke, B.F., Jordan, A.M., et al. (2012). The histone demethylase KDM1A sustains the oncogenic potential of MLL-AF9 leukemia stem cells. Cancer Cell *21*, 473–487.

Heinz, S., Benner, C., Spann, N., Bertolino, E., Lin, Y.C., Laslo, P., Cheng, J.X., Murre, C., Singh, H., and Glass, C.K. (2010). Simple combinations of lineage-determining transcription factors prime cis-regulatory elements required for macrophage and B cell identities. Mol. Cell *38*, 576–589.

Hirose, M., Minato, K., Tobinai, K., Shimoyama, M., Watanabe, S., and Abe, T. (1982). A novel monocytoid cultured cell line, P31/Fujioka, derived from acute monoblastic leukemia. Gan *73*, 735–741.

Huang, L., Chi, J., Berry, F.B., Footz, T.K., Sharp, M.W., and Walter, M.A. (2008). Human p32 is a novel FOXC1-interacting protein that regulates FOXC1 transcriptional activity in ocular cells. Invest. Ophthalmol. Vis. Sci. *49*, 5243–5249.

Huang, X., Spencer, G.J., Lynch, J.T., Ciceri, F., Somerville, T.D., and Somervaille, T.C. (2014). Enhancers of Polycomb EPC1 and EPC2 sustain the oncogenic potential of MLL leukemia stem cells. Leukemia *28*, 1081–1091.

Iwasaki, H., Somoza, C., Shigematsu, H., Duprez, E.A., Iwasaki-Arai, J., Mizuno, S., Arinobu, Y., Geary, K., Zhang, P., Dayaram, T., et al. (2005). Distinctive and indispensable roles of PU.1 in maintenance of hematopoietic stem cells and their differentiation. Blood *106*, 1590–1600.

Jaiswal, S., and Ebert, B.L. (2019). Clonal hematopoiesis in human aging and disease. Science *366*, eaan4673.

Jennings, B.H., and Ish-Horowicz, D. (2008). The Groucho/TLE/Grg family of transcriptional co-repressors. Genome Biol. *9*, 205.

Jongen-Lavrencic, M., Grob, T., Hanekamp, D., Kavelaars, F.G., Al Hinai, A., Zeilemaker, A., Erpelinck-Verschueren, C.A.J., Gradowska, P.L., Meijer, R., Cloos, J., et al. (2018). Molecular Minimal Residual Disease in Acute Myeloid Leukemia. N. Engl. J. Med. *378*, 1189–1199.

Kelly, L.M., Liu, Q., Kutok, J.L., Williams, I.R., Boulton, C.L., and Gilliland, D.G. (2002). FLT3 internal tandem duplication mutations associated with human acute myeloid leukemias induce myeloproliferative disease in a murine bone marrow transplant model. Blood *99*, 310–318.

Khwaja, A., Bjorkholm, M., Gale, R.E., Levine, R.L., Jordan, C.T., Ehninger, G., Bloomfield, C.D., Estey, E., Burnett, A., Cornelissen, J.J., et al. (2016). Acute myeloid leukaemia. Nat. Rev. Dis. Primers *2*, 16010.

Kumar, M., Gouw, M., Michael, S., Sámano-Sánchez, H., Pancsa, R., Glavina, J., Diakogianni, A., Valverde, J.A., Bukirova, D., Čalyševa, J., et al. (2020). ELM-the eukaryotic linear motif resource in 2020. Nucleic Acids Res. *48* (*D1*), D296–D306.

Lancrin, C., Sroczynska, P., Stephenson, C., Allen, T., Kouskoff, V., and Lacaud, G. (2009). The haemangioblast generates haematopoietic cells through a haemogenic endothelium stage. Nature *457*, 892–895.

Lee, T.I., Johnstone, S.E., and Young, R.A. (2006). Chromatin immunoprecipitation and microarray-based analysis of protein location. Nat. Protoc. *1*, 729–748.

Li, H., and Durbin, R. (2009). Fast and accurate short read alignment with Burrows-Wheeler transform. Bioinformatics *25*, 1754–1760.

Li, H., Handsaker, B., Wysoker, A., Fennell, T., Ruan, J., Homer, N., Marth, G., Abecasis, G., and Durbin, R.; 1000 Genome Project Data Processing Subgroup (2009). The Sequence Alignment/Map format and SAMtools. Bioinformatics *25*, 2078–2079.

Love, M.I., Huber, W., and Anders, S. (2014). Moderated estimation of fold change and dispersion for RNA-seq data with DESeq2. Genome Biol. *15*, 550.

Machanick, P., and Bailey, T.L. (2011). MEME-ChIP: motif analysis of large DNA datasets. Bioinformatics *27*, 1696–1697.

Maiques-Diaz, A., Spencer, G.J., Lynch, J.T., Ciceri, F., Williams, E.L., Amaral, F.M.R., Wiseman, D.H., Harris, W.J., Li, Y., Sahoo, S., et al. (2018). Enhancer activation by pharmacologic displacement of LSD1 from GFI1 induces differentiation in acute myeloid leukemia. Cell Rep. *22*, 3641–3659.

Martin, M. (2012). Cutadapt removes adapter sequences from high-throughput sequencing reads. EMBnet.journal *17*, 10–12.

McLean, C.Y., Bristor, D., Hiller, M., Clarke, S.L., Schaar, B.T., Lowe, C.B., Wenger, A.M., and Bejerano, G. (2010). GREAT improves functional interpretation of cis-regulatory regions. Nat. Biotechnol. *28*, 495–501.

Mohammed, H., Taylor, C., Brown, G.D., Papachristou, E.K., Carroll, J.S., and D'Santos, C.S. (2016). Rapid immunoprecipitation mass spectrometry of endogenous proteins (RIME) for analysis of chromatin complexes. Nat. Protoc. *11*, 316–326.

Murphy, T.C., Saleem, R.A., Footz, T., Ritch, R., McGillivray, B., and Walter, M.A. (2004). The wing 2 region of the FOXC1 forkhead domain is necessary for normal DNA-binding and transactivation functions. Invest. Ophthalmol. Vis. Sci. *45*, 2531–2538.

Narita, M., Shimizu, K., Hayashi, Y., Taki, T., Taniwaki, M., Hosoda, F., Kobayashi, H., Nakamura, H., Sadamori, N., Ohnishi, H., et al. (1999). Consistent detection of CALM-AF10 chimaeric transcripts in haematological malignancies with t(10;11)(p13;q14) and identification of novel transcripts. Br. J. Haematol. *105*, 928–937.

Omatsu, Y., Seike, M., Sugiyama, T., Kume, T., and Nagasawa, T. (2014). Foxc1 is a critical regulator of haematopoietic stem/progenitor cell niche formation. Nature *508*, 536–540.

Papachristou, E.K., Kishore, K., Holding, A.N., Harvey, K., Roumeliotis, T.I., Chilamakuri, C.S.R., Omarjee, S., Chia, K.M., Swarbrick, A., Lim, E., et al. (2018). A quantitative mass spectrometry-based approach to monitor the dynamics of endogenous chromatin-associated protein complexes. Nat. Commun. *9*, 2311.

Ptasinska, A., Assi, S.A., Mannari, D., James, S.R., Williamson, D., Dunne, J., Hoogenkamp, M., Wu, M., Care, M., McNeill, H., et al. (2012). Depletion of RUNX1/ETO in t(8;21) AML cells leads to genome-wide changes in chromatin structure and transcription factor binding. Leukemia *26*, 1829–1841.

Quinlan, A.R., and Hall, I.M. (2010). BEDTools: a flexible suite of utilities for comparing genomic features. Bioinformatics *26*, 841–842.

Somervaille, T.C., Matheny, C.J., Spencer, G.J., Iwasaki, M., Rinn, J.L., Witten, D.M., Chang, H.Y., Shurtleff, S.A., Downing, J.R., and Cleary, M.L. (2009). Hierarchical maintenance of MLL myeloid leukemia stem cells employs a transcriptional program shared with embryonic rather than adult stem cells. Cell Stem Cell *4*, 129–140.

Somerville, T.D., Wiseman, D.H., Spencer, G.J., Huang, X., Lynch, J.T., Leong, H.S., Williams, E.L., Cheesman, E., and Somervaille, T.C. (2015). Frequent Derepression of the Mesenchymal Transcription Factor Gene FOXC1 in Acute Myeloid Leukemia. Cancer Cell *28*, 329–342.

Sood, R., Kamikubo, Y., and Liu, P. (2017). Role of RUNX1 in hematological malignancies. Blood *129*, 2070–2082.

Sperling, A.S., Gibson, C.J., and Ebert, B.L. (2017). The genetics of myelodysplastic syndrome: from clonal haematopoiesis to secondary leukaemia. Nat. Rev. Cancer *17*, 5–19.

Subramanian, A., Tamayo, P., Mootha, V.K., Mukherjee, S., Ebert, B.L., Gillette, M.A., Paulovich, A., Pomeroy, S.L., Golub, T.R., Lander, E.S., and Mesirov, J.P. (2005). Gene set enrichment analysis: a knowledge-based approach for interpreting genome-wide expression profiles. Proc. Natl. Acad. Sci. USA *102*, 15545–15550.

Suzuki, H., Forrest, A.R., van Nimwegen, E., Daub, C.O., Balwierz, P.J., Irvine, K.M., Lassmann, T., Ravasi, T., Hasegawa, Y., de Hoon, M.J., et al.; FANTOM Consortium; Riken Omics Science Center (2009). The transcriptional network that controls growth arrest and differentiation in a human myeloid leukemia cell line. Nat. Genet. *41*, 553–562.

Sykes, S.M., Lane, S.W., Bullinger, L., Kalaitzidis, D., Yusuf, R., Saez, B., Ferraro, F., Mercier, F., Singh, H., Brumme, K.M., et al. (2011). AKT/FOXO signaling enforces reversible differentiation blockade in myeloid leukemias. Cell *146*, 697–708.

Telfer, J.C., and Rothenberg, E.V. (2001). Expression and function of a stem cell promoter for the murine CBFalpha2 gene: distinct roles and regulation in natural killer and T cell development. Dev. Biol. *229*, 363–382.

van Gosliga, D., Schepers, H., Rizo, A., van der Kolk, D., Vellenga, E., and Schuringa, J.J. (2007). Establishing long-term cultures with self-renewing acute myeloid leukemia stem/progenitor cells. Exp. Hematol. *35*, 1538–1549.

Vassiliou, G.S., Cooper, J.L., Rad, R., Li, J., Rice, S., Uren, A., Rad, L., Ellis, P., Andrews, R., Banerjee, R., et al. (2011). Mutant nucleophosmin and cooperating pathways drive leukemia initiation and progression in mice. Nat. Genet. *43*, 470–475.

Warren, R.W., Nagy, L., Selegue, J., Gates, J., and Carroll, S. (1994). Evolution of homeotic gene regulation and function in flies and butterflies. Nature *372*, 458–461.

Wilhelmson, A.S., and Porse, B.T. (2020). CCAAT enhancer binding protein alpha (CEBPA) biallelic acute myeloid leukaemia: cooperating lesions, molecular mechanisms and clinical relevance. Br. J. Haematol. *190*, 495–507.

Wiseman, D.H., Williams, E.L., Wilks, D.P., Sun Leong, H., Somerville, T.D., Dennis, M.W., Struys, E.A., Bakkali, A., Salomons, G.S., and Somervaille, T.C. (2016). Frequent reconstitution of IDH2(R140Q) mutant clonal multilineage hematopoiesis following chemotherapy for acute myeloid leukemia. Leukemia *30*, 1946–1950.

Zhang, D.E., Hetherington, C.J., Meyers, S., Rhoades, K.L., Larson, C.J., Chen, H.M., Hiebert, S.W., and Tenen, D.G. (1996). CCAAT enhancer-binding protein (C/EBP) and AML1 (CBF alpha2) synergistically activate the macrophage colony-stimulating factor receptor promoter. Mol. Cell. Biol. *16*, 1231–1240.

Zhang, Y., Liu, T., Meyer, C.A., Eeckhoute, J., Johnson, D.S., Bernstein, B.E., Nusbaum, C., Myers, R.M., Brown, M., Li, W., and Liu, X.S. (2008). Model-based analysis of ChIP-Seq (MACS). Genome Biol. *9*, R137.

CellPress
OPEN ACCESS

STAR★METHODS

KEY RESOURCES TABLE

REAGENT or RESOURCE	SOURCE	IDENTIFIER
Antibodies		
anti-monomethyl-H3K4	Abcam	Cat#ab8895; RRID:AB_306847
anti-dimethyl-H3K4	Abcam	Cat#ab7766; RRID:AB_2560996
anti-acetyl H3K27	Abcam	Cat#ab4729; RRID:AB_2118291
anti-HDAC1	Abcam	Cat#ab46985; RRID:AB_880347
anti-RUNX1	Abcam	Cat#ab23980; RRID:AB_2184205
anti-EP300	Abcam	Cat#ab14984; RRID:AB_301550
anti-TLE3	Abcam	Cat#ab94972; RRID:AB_10860535
anti-CBFb	Abcam	Cat#ab125191; RRID:AB_10999861
anti-MYB	Abcam	Cat#ab117635; RRID:AB_10900735
Anti-FLAG	Sigma	Cat#F3165; RRID:AB_259529
anti-SP1	Abcam	Cat#ab13370; RRID:AB_300283
anti-SMARCC2	Cell Signaling	Cat#12760; RRID:AB_2798017
anti-SPI1	Cell Signaling	Cat#2258; RRID:AB_2186909
anti-Myc tag	Cell Signaling	Cat#2278; RRID:AB_490778
anti-RUNX1	Cell Signaling	Cat#8529; RRID:AB_10950225
anti-STAT3	Cell Signaling	Cat#30835; RRID:AB_2798995
anti-CEBPA	Diagenode	Cat#C15410225; RRID:AB_2737367
anti-FOXC1	This study	In house
anti-ACTB	Millipore	Cat#MAB1501; RRID:AB_2223041
anti-KLF2	Millipore	Cat#09-820; RRID:AB_10807287
IgG Goat	Millipore	Cat#NI02; RRID:AB_11213183
IgG Rabbit	Merk	Cat#12-307;
anti-CD11b-PE	Biosciences	Cat#557321; RRID:AB_396636
anti-CD14-FITC	Biosciences	Cat#557153; RRID:AB_396589
anti-CD86-PerCP-eFlour710	ThermoFisher	Cat#46086282; RRID:AB_2815140
Bacterial and virus strains		
Rosetta BL21(DE3) Competent cells	Merck	Cat#70954
One shot Stbl3 Chemically Competent E.coli	NEB	Cat#C737303
Biological samples		
Primary human AML samples	Manchester Cancer Research Centre Tissue Biobank	the-christie.biobank@nhs.net
Chemicals, peptides, and recombinant proteins		
Doxycycline	Sigma	Cat#24390-14-5
Puromycin	Sigma	Cat#P8833
Methylcellulose medium	Stem Cell Technologies	Cat#04230
Hydrocortisone	Merck	Cat#31719
IL3	Peprotech	Cat#300-03
G-CSF	Peprotech	Cat#300-23
TPO	Peprotech	Cat#300-18
Dynabeads Protein G	Invitrogen	Cat#10004D
Protein G Sepharose	Sigma	Cat#P3296
T4 DNA Ligase	NEB	Cat#M0202L
ECL Western Blotting Reagent	GE Healthcare	Cat#RPN2209

(Continued on next page)

Continued		
REAGENT or RESOURCE	SOURCE	IDENTIFIER
Polybrene	Milipore	Cat#TR100G
Trypan Blue Solution	GIBCO	Cat#15250-061
10% tetracycline-free FBS	Lonza	Cat#77227
IPTG	Fluorochem	Cat#M02726
EcoR1-HF	NEB	Cat#R3101L
Xho1	NEB	Cat#R0146L
Age1	NEB	Cat#R3552S
Spe1	NEB	Cat#R3133L
Dpn1	NEB	Cat#R0176S
Taqman Fast Universal PCR Mastermix	Applied Biosystems	Cat#4352042
Benzonase	Sigma	Cat#B1014
di(N-succinimidyl)glutarate	Sigma	Cat#80424
ChIP Crosslink Gold	Diagenode	Cat#C01019027
May-Grunwald Giemsa	Sigma	Cat#MG500
Alt-R Cas9	Integrated DNA Technologies	Cat#108158
AMPure XP	Beckman Coulter	Cat# A63880
Critical commercial assays		
AminoLink Plus Immobilisation Kit	ThermoFisher	Cat#44894
APC Annexin Kit	BD Pharmigen	Cat#550474
RNeasy Plus Micro Kit	QIAGEN	Cat#74034
Nextera Sample preparation kit	Illumina	Cat#FC-131-1096
High-Capacity cDNA Reverse Transcription	ThermoFisher	Cat#4368814
Pierce BCA Protein Assay Kit	ThermoFisher	Cat#23225
DNeasy blood and tissue kit	QIAGEN	Cat#69504
QIAshredder	QIAGEN	Cat#79656
RNeasy Plus Micro Kit	QIAGEN	Cat#74034
RNeasy Plus Mini Kit	QIAGEN	Cat#74134
Neon Transfection System	ThermoFisher	Cat#MPK10096
Microplex Library Preparation Kit	Diagenode	Cat#C05010012
NextSeq 500/550 High Output v2 kit	Illumina	Cat#20024906
TruSeq Stranded Total RNA with Ribo-Zero Globin	Illumina	Cat#20020612
Deposited ata		
Proteomics data	This study	ProteomeXchange: PXD027740
All DNA and RNA sequencing data	This study	GEO: GSE159693
TS41_Fujioka _NTC1	This study	GSM4837692
TS41_Fujioka _NTC2	This study	GSM4837693
TS41_Fujioka _FOXC1 KD1	This study	GSM4837694
TS41_Fujioka _FOXC1 KD2	This study	GSM4837695
TS85_ FOXC1_BB475	This study	GSM4837696
TS49_ H3K27Ac_ NTC	This study	GSM4837697
TS49_ H3K27Ac_ KD	This study	GSM4837698
TS49_ H3K4me2_ NTC	This study	GSM4837699
TS49_ H3K4me2_ KD	This study	GSM4837700
TS53_ATAC_NTC	This study	GSM4837701
TS53_ATAC_KD	This study	GSM4837702
TS69_FOXC1	This study	GSM4837703
TS83_H3K4me1	This study	GSM4837704
TS105_SPI1	This study	GSM4837705

(*Continued on next page*)

Continued

REAGENT or RESOURCE	SOURCE	IDENTIFIER
TS120_HDAC1_NTC	This study	GSM4837706
TS120_HDAC1_KD	This study	GSM4837707
TS120_RUNX1_NTC	This study	GSM4837708
TS120_RUNX1_KD	This study	GSM4837709
TS120_CEBPA_NTC	This study	GSM4837710
TS120_CEBPA_KD	This study	GSM4837711
TS120_P300_NTC	This study	GSM4837712
TS120_P300_KD	This study	GSM4837713
TS120_SMARCC2_NTC	This study	GSM4837714
TS120_SMARCC2_KD	This study	GSM4837715
TS147_TLE3_NTC	This study	GSM4837716
TS147_TLE3_KD	This study	GSM4837717
TS159_01_Fox_C1_KD	This study	GSM5366264
TS154_01_BB171_FOXC1	This study	GSM5416297
TS154_01_BB171_RUNX1	This study	GSM5416298
TS154_01_BB171_Input	This study	GSM5416299
Experimental models: Cell lines		
THP1	DSMZ	RRID:CVCL_0006
HL60	DSMZ	RRID:CVCL_0002
Fujioka	JCRB	RRID:CVCL_1632
K562	DSMZ	RRID:CVCL_0004
MonoMac-1	DSMZ	RRID:CVCL_1425
MV-4-11	ATCC	RRID:CVCL_0064
Kasumi-1	DSMZ	RRID:CVCL_0589
HEK293	Invitrogen	RRID:CVCL_0045
Oligonucleotides		
See Table S4	This study	N/A
Recombinant DNA		
pET-28: FOXC1 Δ(69-178)	This study	N/A
pLKO.1: NTC	This study	N/A
pLKO.1: *FOXC1* KD	This study	N/A
pLKO.1: *ADPN* KD1	This study	N/A
pLKO.1: *ADPN* KD2	This study	N/A
pLKO.1: *ARID3A* KD1	This study	N/A
pLKO.1: *ARID3A* KD2	This study	N/A
pLKO.1: *CBFB* KD1	This study	N/A
pLKO.1: *CBF2* KD2	This study	N/A
pLKO.1: *CEBPA* KD1	This study	N/A
pLKO.1: *CEBPA* KD2	This study	N/A
pLKO.1: *CEBPE* KD1	This study	N/A
pLKO.1: *CEBPE* KD2	This study	N/A
pLKO.1: *ELF1* KD1	This study	N/A
pLKO.1: *ELF1* KD2	This study	N/A
pLKO.1: *ETV6* KD1	This study	N/A
pLKO.1: *ETV6* KD2	This study	N/A
pLKO.1: *HOXA10* KD1	This study	N/A
pLKO.1: *HOXA10* KD2	This study	N/A
pLKO.1: *IKZF1* KD1	This study	N/A

(*Continued on next page*)

Continued

REAGENT or RESOURCE	SOURCE	IDENTIFIER
pLKO.1: *IKZF1* KD2	This study	N/A
pLKO.1: *MAX* KD1	This study	N/A
pLKO.1: *MAX* KD2	This study	N/A
pLKO.1: *RUNX1* KD1	This study	N/A
pLKO.1: *RUNX1* KD2	This study	N/A
pLKO.1: *SPI1* KD1	This study	N/A
pLKO.1: *SPI1* KD2	This study	N/A
pLKO.1: *STAT3* KD1	This study	N/A
pLKO.1: *STAT3* KD2	This study	N/A
pLKO.1: *TLE3* KD1	This study	N/A
pLKO.1: *FOXK2* KD1	This study	N/A
pLKO.1: *FOXN2* KD1	This study	N/A
pLKO.1: *FOXN2* KD2	This study	N/A
pLKO.1: *FOXJ3* KD1	This study	N/A
pLKO.1: *FOXJ3* KD2	This study	N/A
pLenti-GS-minCMV-TET-blasticidin: FOXC1	This study	N/A
pLenti-GS-minCMV-TET-blasticidin: FOXC1 Δ(69-178)	This study	N/A
pLenti-GS-minCMV-TET-blasticidin: FOXC1 Δ(1-50)	This study	N/A
pLenti-GS-minCMV-TET-blasticidin: FOXC1 Δ(215-366)	This study	N/A
pLenti-GS-minCMV-TET-blasticidin: FOXC1 Δ(436-553)	This study	N/A
pLenti-GS-minCMV-TET-blasticidin: FOXC1 G165R	This study	N/A
pLenti-GS-minCMV-TET-blasticidin: FOXC1 F112S	This study	N/A
pLenti-GS-minCMV-TET-blasticidin: FOXC1-DBD-RUNX1c	This study	N/A
pLenti-GS-minCMV-TET-blasticidin: KLF2	This study	N/A
pRetroX-Tet-On Advanced	Clontech	Cat#632104
pcDNA3.1: RUNX1b	This study	N/A
pcDNA3.1: RUNX1b Δ(1-56)	This study	N/A
pcDNA3.1: RUNX1b Δ(242-451)	This study	N/A
pcDNA3.1: RUNX1b Δ(186-241) Δ(372-451)	This study	N/A
pcDNA3.1: RUNX1b Δ(50-175)	This study	N/A
pcDNA3.1: RUNX1b Δ(372-451)	This study	N/A
Software and algorithms		
STAR	Dobin et al., 2013	N/A
DESeq2	Love et al., 2014	N/A
MACS2	Zhang et al., 2008	N/A
Homer version 4.10	Heinz et al., 2010	N/A
SDS software v2.1	Applied Biosystems	N/A
FASTQC	Andrews, 2010	N/A
Samtools	Li et al., 2009	N/A
Cutadapt	Martin, 2012	N/A
GREAT	McLean et al., 2010	N/A

(*Continued on next page*)

Continued

REAGENT or RESOURCE	SOURCE	IDENTIFIER
GSEA v2.0.14	Subramanian et al., 2005	N/A
MEME-ChIP	Machanick & Bailey, 2011	N/A
Microsoft Excel 2007	N/A	N/A
StatsDirect software (v.1.9.7)	StatsDirect	N/A
QuantStudio Real Time PCR system	ThermoFisher	N/A

RESOURCE AVAILABILITY

Lead contact

- Further information and requests for resources and reagents should be directed to and will be fulfilled by the lead contact, Tim Somervaille (tim.somervaille@cruk.manchester.ac.uk).

Materials availability

- In this work, the newly generated material is represented by mammalian expression plasmids generated by Fabrizio Simeoni and Gary Spencer, and the FOXC1 antibody generated by Fabrizio Simeoni. All plasmids listed in the Key resources table are available upon request. Aliquots of the FOXC1 antibody are available on request, subject to a Material Transfer Agreement.

Data and code availability

- ChIP-seq and RNA-seq data have been deposited at GEO and are publicly available as of the date of publication. Proteomics data have been deposited at the Proteomics Identifications Database (PRIDE). Accession numbers are listed in the Key resources table.
- This paper does not report original code.
- Any additional information required to reanalyze the data reported in this paper is available from the lead contact on request.

EXPERIMENTAL MODEL AND SUBJECT DETAILS

Cell lines

Cell lines used were THP1 (M), HL60 (F) and MonoMac-1 (M), MV(4;11) (M), Fujioka (M), HEK293 (F), K562 (F) and Kasumi-1 (M). The sex of the cell lines is indicated by either M (male) or F (female). Leukaemia cell lines were cultured in 90% RPMI, 10% FBS and 2mM glutamine. HEK293 cells were cultured in 90% DMEM, 10% FBS and 2mM glutamine. Cell line identity was verified where possible by STR analysis.

Human tissue and ethical approvals

Use of human tissue was in compliance with the ethical and legal framework of the UK's Human Tissue Act, 2004. Primary human AML samples were from Manchester Cancer Research Centre's Tissue Biobank and used with the informed consent of donors. The Biobank holds a generic ethics approval (18/NW/0092) which can be conferred to users of banked samples via the MCRC Biobank Access Policy. Samples used in this project were approved for use under application number 08_TISO-02. Details of primary samples used are in Table S1.

Cryopreserved primary leukemic blast cells from blood or bone marrow of patients were thawed and co-cultured on MS5 stromal cells in alpha-MEM medium supplemented with 12.5% heat-inactivated FBS, 12.5% heat-inactivated horse serum, 2mM L-glutamine, 57.2mM β-mercaptoethanol, 1 μM hydrocortisone and IL3, G-CSF and TPO (all at 20ng/ml) (van Gosliga et al., 2007). For clonogenic assays, Fujioka cells were cultured at a density of 2-5x10^3 cells/ml in methylcellulose medium (H4320, Stem Cell Technologies, Vancouver, Canada). Clonogenic assays of primary AML cells were performed in the same methylcellulose medium with addition of IL3, G-CSF and TPO (all at 20ng/ml) with puromycin where appropriate (3 μg/ml), as described (Somerville et al., 2015). Cytospin preparations were stained with May-Grunwald Giemsa (Sigma, St Louis, MO).

METHOD DETAILS

Antibodies

For western blotting (WB), all antibodies were used at a dilution of 1:1000 except anti-Myc tag (1:2000) and anti-ACTB (1:10,000). An anti-RUNX1 antibody from Abcam was used for RUNX1 ChIP-seq, ChIP-qPCR and immunoprecipitation experiments, while an anti-RUNX1 antibody from Cell Signaling was used for WB.

FOXC1 antibody production

To generate a bacterial expression vector for FOXC1, FOXC1 cDNA (NM_001453) was excised from pcDNA3.1-FOXC1 (a gift from Jane Sowden) using EcoR1 and Xho1 restriction sites and sub-cloned into the EcoR1/Xho1 sites of pET-28 (a gift from Iain Hagan). To generate the FOXC1 mutant FOXC1 Δ(69-178), site direct mutagenesis was performed using overlap extension PCR followed by Dpn1 digestion with pET-28-FOXC1 as a template. The vector was then transformed into Rosetta BL21 (DE3) Competent Cells and spread on agar plates containing kanamycin and chloramphenicol, and incubated overnight at 37°C. Cells from a single colony were grown up overnight, diluted to an OD (600nm) of 0.05 and then grown on until the OD reached 0.4. One liter of cells was then supplemented with 1mM of isopropyl β-D-1-thiogalactopyranoside (IPTG) and incubated at 37°C for three hours. Cells were harvested and suspended in bacterial lysis buffer and then subjected to a freeze-thaw cycle. The following day, the sample was defrosted at 37°C for 30 minutes and then supplemented with 4mg of lysozyme and incubated again at 37°C for 30 minutes. Following this second incubation, the sample was supplemented with 10 μg/ml DNase1 and left at room temperature for 30 minutes. Next, 1ml of 10% sodium deoxycholate and 1% Triton were added to the tube and the sample was incubated on ice for 15 minutes. Finally, the lysate was centrifuged at 10000rpm for 15 minutes and the inclusion bodies (pellet) was suspended in 1.5ml PBS supplemented with 10x NuPAGE sample reducing agent and 4x NuPAGE lithium dodecyl sulfate (LDS) sample loading buffer. Recovered proteins were subjected to acrylamide gel electrophoresis and Coomassie gel staining, and the protein band corresponding to FOXC1 was excised. The excised band was then placed into a dialysis membrane and protein eluted overnight at 100mA. The purified protein solution was then transferred into a new dialysis membrane overnight in order to eliminate SDS. Following dialysis, the protein concentration was calculated using a Pierce BCA Protein Assay Kit and 2μg FOXC1 protein was then provided for goat immunisation at Eurogentec (Liege, Belgium) using the Speedy 28-Day program of immunisation.

Antibodies were purified from the resulting goat serum using an AminoLink Plus Immobilization Kit according to manufacturer's instructions. After column elution, the purified antibody was dialysed in PBS, supplemented with an equal volume of glycerol 100% and 0.05% of sodium azide, and stored at −80°C.

Flow cytometry, protocols and antibodies

Flow cytometry analyses were performed using a LSR Model II flow cytometer (BD Biosciences, Oxford, UK). Antibodies used for flow cytometry were all used at a dilution of 1/200. Apoptosis was assessed using a BD PharMingen APC Annexin V kit. Propidium iodide cell cycle analyses were performed as described (Somerville et al., 2015). Throughout the study, geometric mean cell fluorescence values are used.

Protein extraction, western blotting and IP

Cells to be lysed were first counted, pelleted by centrifugation and resuspended twice in ice cold PBS in order to wash away media and any debris from cell culture. Cells were lysed in ice-cold high salt lysis buffer (45mM HEPES (pH 7.5), 400mM NaCl, 1mM EDTA, 10% Glycerol, 0.5% NP40, 6.25mM NaF, 20mM β-glycerophosphate, 1mM DTT, 20mM sodium butyrate and 1x Protease Inhibitor cocktail (Roche, Burgess Hill, UK)), typically at concentrations of $1x10^6$ cells in 50 μL lysis buffer. Samples were then centrifuged at 20,000xg at 4°C for 15 minutes to pellet cell debris, and the supernatant was then collected. Lysates were stored at −80°C. Equal amounts of protein were loaded and separated by SDS-PAGE.

Immunoprecipitation (IP) experiments were performed using lysate generated from Fujioka AML cells and 293 cells. Cells were washed twice with ice-cold PBS and lysed in 1mL (for 10 million cells) of ice-cold TNN buffer (50mM Tris-Cl (pH7.5), 100mM NaCl, 5mM EDTA, 0.5% Nonidet p40) supplemented with 6.25mM NaF, 20mM β-glycerophosphate, 1mM DTT and 1 μL Benzonase® nuclease (Sigma Aldrich), by rotation at 40rpm at 4°C for 15 minutes. Samples were then centrifuged at 20,000xg at 4°C for 15 minutes to pellet cell debris, and 10 μL of the supernatant per sample was taken for input control with the rest being used for IP. For the IP, Protein G Sepharose® Fast Flow beads (Sigma Aldrich; 20 μL for 10 million cells) were washed three times in TNN buffer before being resuspended in 500 μL TNN buffer with the appropriate antibody or isotype control. Antibodies were used at 10 μg per 100 million cells.

The beads were incubated with antibodies overnight at 4°C with constant rotation. Following this incubation, beads were centrifuged at 1700xg at 4°C for 1 minute, combined with the prepared lysates and rotated at 40rpm at 4°C overnight. The following day the antibody-bound beads were centrifuged (1700xg at 4°C for 1 minute) and washed four times in 1mL TNN buffer before being resuspended in 20 μL elution buffer (10x NuPAGE® sample reducing agent, 4x NuPAGE® LDS). Proteins bound to the beads were eluted by heating the samples for 10 minutes at 70°C and the beads were subsequently removed by centrifuging the sample through a 0.45 μm Spin-X® centrifuge tube filter within a 2mL DNase/RNase-free polypropylene tube (Costar®, Corning). Immunoprecipitated and co-immunoprecipitated proteins were assayed by western blotting.

For western blotting, proteins were separated by SDS-PAGE. Equal amounts of lysate were diluted in ddH_2O containing 10x NuPAGE® sample reducing agent and 4xNuPAGE® lithium dodecyl sulfate (LDS) sample loading buffer (both from Life Technologies). Samples were then incubated at 95°C for 10 minutes in order to ensure complete unfolding of the protein secondary structure. Lysates were then loaded into pre-cast NuPAGE® 4%–12% Bis-Tris acrylamide gels in a gel tank filled with 1x MOPS® running buffer (50mM MOPS, 50mM Tris Base, 0.1% SDS, 1 mM EDTA, pH 7.7) to ensure electric conduction. Gels, tanks and MOPS® running buffer were all from Life Technologies. For molecular weight estimation, 5 μL of PageRuler Plus prestained protein ladder (ThermoFisher) was run

together with the samples. Empty wells were filled with 4xNuPAGE® LDS loading buffer diluted in ddH_2O to ensure an even run of the samples. Gels were electrophoresed for at 150 V for approximately 1 hour.

Following electrophoresis, the pre-cast gel was transferred to a nitrocellulose membrane (Whatman Protram® - https://www.ge.com). Transfer was performed at 4°C at 70 V for 1 hour 15 minutes in a semi-dry transfer tank (Bio-Rad) filled with transfer buffer. Transfer buffer was prepared by diluting 50mL of transfer buffer 10x solution (30 g Tris and 143 g glycine made up to 1L with deionised water) and 100mL of methanol (Fisher Scientific) with deionised water to a final volume of 500 mL. Following completion of transfer the nitrocellulose membrane was stained with Ponceau Red (Sigma Aldrich) to confirm equal loading of the samples and successful transfer to the nitrocellulose membrane.

Following Ponceau Red staining, membranes were rinsed with tap water and cut with a sterile scalpel to isolate proteins of the appropriate molecular weight for subsequent staining. Ponceau Red was washed away with 1x PBS-Tween (prepared from a 20x stock solution consisting of 560 g NaCl, 14 g KCl, 100.8g Na_2HPO_4, 16.8g KH_2PO_4, 70mL Tween20 diluted in deionised water to a final volume of 10L), prior to blocking with 5% skimmed milk in 1x PBS-Tween for 30 minutes at room temperature. Residual milk was washed away with 1x PBS- Tween and primary antibody incubation was performed on rollers at 4°C overnight. Primary and secondary antibodies were diluted in 5% BSA (Sigma Aldrich) and 2% Western Blocking reagent (Roche) in 1x PBS-Tween. After 3x 10 minutes washes with 1x PBS-Tween, membranes were incubated with secondary Horseradish peroxidase (HRP)-linked secondary antibodies (GE Healthcare - https://www.gelifesciences.com) on rollers for 1 hour at room temperature. After 3x 10 minutes washes with 1x PBS-Tween, membranes were incubated with either ECL (enhanced chemiluminescence; GE Healthcare) or Supersignal (Pierce, Rockford, IL, USA) and the signal generated by the HRP-conjugated immune complexes was exposed using a high performance chemiluminescence film (AmershamTM Hyperfilm - https://www.ge.com) and an X-ray cassette and detected using a Curix 60 film processor (AGFA - https://global.agfahealthcare.com/) in a dark room.

Mass Spectrometry

For Rapid Immunoprecipitation Mass spectrometry of Endogenous protein (RIME), Fujioka cells (1×10^8) were grown in RPMI with 10% FBS. The medium was then removed and replaced with PBS containing 2mM di(N-succinimidyl) glutarate (DSG) and crosslinked for 30 minutes. PBS and DSG were then removed and cells were washed twice in PBS. Cell were then further crosslinked in PBS containing 1% formaldehyde for 10 minutes. Crosslinking was quenched by adding glycine to a final concentration of 0.125M. Cells were then washed with ice-cold PBS. The nuclear fraction was extracted by first suspending the pellet in 10ml of LB1 buffer (50mM HEPES-KOH [pH 7.5], 140mM NaCl, 1mM EDTA, 10% glycerol, 0.5% NP-40 or Igepal CA-630, and 0.25% Triton X-100) for 10 min at 4°C. Cells were pelleted and suspended in 10ml of LB2 buffer (10mM Tris-HCL [pH 8.0], 200mM NaCl, 1mM EDTA, and 0.5mM EGTA) and mixed at 4°C for 5 minutes. Cells were pelleted and suspended in 300ml of LB3 buffer (10mM Tris-HCl [pH 8], 100mM NaCl, 1mM EDTA, 0.5mM EGTA, 0.1% Na deoxycholate and 0.5% N-lauroylsarcosine) and sonicated using a Bioruptor Pico (Diagenode, Liege, Belgium) for 8 cycles, with 30 s ON, 30 s OFF settings. Triton X-100 was added at 10% concentration and lysate was centrifuged for 10 minutes at 20,000*rcf.* to purify the debris. The supernatant was then incubated with Dynabeads (Protein G) pre-bound with 10 μg antibody and IP was conducted overnight at 4°C. The beads were washed ten times in 1ml of RIPA buffer and twice in 100mM ammonium hydrogen carbonate (AMBIC) solution. For the second AMBIC wash, beads were transferred to new tubes. RIME samples were prepared and analyzed by mass spectrometry as described (Mohammed et al., 2016; Papachristou et al., 2018; Glont et al., 2019). Briefly, the proteins bound to the beads were digested by adding trypsin prepared in 100mM ammonium bicarbonate buffer. Samples were incubated overnight at 37°C followed by a second-step of digestion the next day for four hours. Samples were acidified with the addition of 5% formic acid and purified using C18 columns according to manufacturer's instructions (Harvard Apparatus, Cambridge, UK). After purification, samples were dried with SpeedVac and reconstituted in 15μl of 0.1% formic acid. A volume of 5μl of each sample was injected on the Dionex Ultimate 3000 UHPLC system coupled with the Q-Exactive mass spectrometer. The full MS scan on Q-Exactive was at 70K resolution and the MS2 scans were performed at 35K resolution with collision energy 28% and isolation window 2.0Th. For the HCD data processing, the SequestHT search engine implemented on Proteome Discoverer 1.4 software was used with Precursor Mass Tolerance 20ppm and Fragment Mass Tolerance 0.02Da. Dynamic modifications were oxidation of M (+15.995Da) and deamidation of N/Q (+0.984Da).

Expression constructs and vectors

Lentiviral vectors for KD experiments (non-targeting control pLKO.1 (SHC002), FOXC1 KD3 pLKO.2 (TRCN0000235693) and a lentiviral vector for expression of FOXC1 cDNA resistant to KD) were from Somerville at al., 2015.

To generate lentiviral KD constructs, pLKO.1 puro was digested with Age1 and EcoR1 and ligated with HPLC purified oligonucleotides previously annealed by incubating at 98°C for 5 minutes and slowly cooling to room temperature.

To generate doxycycline-inducible Fujioka cells, a lentiviral plasmid expressing the rtTA protein under the control of the EF1α promoter was generated by cloning the rtTA-IRES-neomycin expression cassette from pRetroX-Tet-On Advanced into the BamH1/Sal1 sites of pLentiGS (Huang et al., 2014). Fujioka cells constitutively expressing rtTA protein were generated by infection with pLentiGS EF1α- rtTA-IRES-neo followed by neomycin selection (500 μg/ml) for two weeks.

To generate the tetracycline inducible FOXC1 lentiviral expression construct, human FOXC1 cDNA was PCR amplified from pcDNA3.1-FOXC1 (a gift from Jane Sowden) using oligonucleotides which introduced coding sequences for a C-terminal GSG linker

and Myc tag. Oligonucleotide sequences are shown in Table S4. The product was subcloned into pGEM-T. The sequence was verified and cDNA was excised and ligated into the EcoR1 and Spe1 sites of pLentiGS-minCMV-TET-blasticidin (Huang et al., 2014).

To generate FOXC1 mutants FOXC1 Δ(1-50) and FOXC1 Δ(436-553), FOXC1 cDNA was amplified from pLentiGS-minCMV-TET-blasticidin vector-FOXC1-Myc using the oligonucleotide sequences shown in Table S4. The products were then digested using EcoR1 and Spe1 and ligated into the pLentiGS-minCMV-TET-blasticidin vector.

To generate FOXC1 mutants FOXC1 Δ(69-178), FOXC1 Δ(215-366), FOXC1 G165R and FOXC1 F112S, site direct mutagenesis was performed using overlap extension PCR followed by Dpn1 digestion with pLentiGS-minCMV-TET-blasticidin vector-FOXC1-Myc as a template. Oligonucleotide primer sequences are shown in Table S4.

To generate a doxycycline-inducible FOXC1-DBD RUNX1c lentiviral fusion construct, the DNA binding domain (DBD) of FOXC1 was PCR amplified using the following oligonucleotide primers and full length FOXC1 as template. Oligonucleotide primer sequences are shown in Table S4. The amplicon was subcloned into pGEM-T, sequence verified, excised and cloned into the EcoR1/Nhe1 sites of the doxycycline-inducible lentiviral vector pLentiGS-minCMV-TET-blasticidin (Huang et al., 2014). Full length *RUNX1c* was then PCR amplified using the oligonucleotides shown in Table S4 and then subcloned into pGEM-T, sequence verified, excised and cloned into Nhe1/Cla1 sites of FOXC1 DBD pLentiGS-minCMV-TET-blasticidin.

To generate tetracycline inducible KLF2 lentiviral expression construct, human KLF2 cDNA was PCR amplified from Fujioka cells cDNA using the oligonucleotides shown in Table S4. The product was subcloned into pGEM-T. Sequence was verified and cDNA was excised and ligated into the EcoRI and Xba1 sites of pLentiGS-minCMV-TET-blasticidin vector. rtTA Fujioka cells were then infected with doxycycline-inducible vectors and selected for 10 days in blasticidin (6 μg/ml). Cells were maintained in RPMI containing 10% tetracycline-free FBS in the presence of 250 μg/ml neomycin and 3 μg/ml blasticidin. Protein expression was induced using 1 μg/ml doxycycline.

To generate RUNX1b mutants, pcDNA3.1_Runx1b was created by cloning a PCR amplified FLAG-tagged (DYKDDDDK) murine proximal Runx1 isoform (Runx1b) cDNA (Telfer and Rothenberg, 2001) into mammalian expression vector pcDNA3.1 (Invitrogen) using the restriction enzymes BglII and Xho1. pcDNA3.1_Runx1b was used as a template to construct additional pcDNA3.1 vectors containing truncated Runx1b cDNA sequences (Δ amino acids: 1-56, 243-451, 372-451, 186-241+372-451, 50-175) using either standard PCR and/or site directed mutagenesis. Oligonucleotide primer sequences are shown in Table S4.

Viral particle manufacture

Lentiviral supernatants were prepared and leukemic human cells were infected with viral particles as previously described (Harris et al., 2012). Briefly, the day prior to transfection, 293FT cells were plated in 10cm dishes at a density of 4.5×10^6 cells per dish in 9mL DMEM with 10% FBS (D10). Next day cells were typically at ~90% confluence. For the transfection, 21 μg polyethylenimine (PEI) was diluted in 500 μL serum-free DMEM at room temperature for each 10cm dish. For the manufacture of lentiviral particles, plasmids containing viral structural genes were combined with lentiviral expression plasmids and diluted as follows: lentiviral vector - 4 μg, pCMVD8.91 - 2 μg, pMDG.2 - 1 μg, serum-free DMEM to 500 μl. Equal volumes of the diluted PEI and plasmid constructs were then combined, mixed by pipetting and left to incubate for 20-30 minutes at room temperature to allow formation of DNA-PEI complexes. The mixture was then added dropwise to 293FT cells. Next day the medium was replaced with 10mL of fresh, pre-warmed D10 medium per dish prior to further overnight incubation and subsequent harvest of viral particle-containing supernatants. All viral supernatants were filtered through a 0.45 μm polyethersulfone filter prior to use. Lentiviral supernatants were either used immediately or stored long-term at −80°C.

To increase transduction efficiency of target cells Polybrene was added to all viral supernatants to a final concentration of 8 μg/mL. For lentiviral infection of primary human cells or human cell lines $0.5\text{-}1 \times 10^6$ cells or 1.5×10^6 cells respectively were resuspended in 6ml viral supernatant and centrifuged for 30 minutes at 900xg and 37°C. After centrifugation cell line cells were incubated at standard conditions overnight. The following morning, cells were pelleted and resuspended in 10ml of R10 to reduce Polybrene toxicity. For human cell lines infected with lentiviral shRNA constructs, 24 hours following spinoculation 3 μg/mL puromycin (Sigma-Aldrich) was added for 48 hours to select for successfully transduced cells prior to further manipulation. For primary AML cells, after spinoculation, cells were incubated overnight in AML culture medium with cytokines, without stromal support. The following morning, cells were spinoculated a second time as above. Next day 3 μg/mL puromycin was added for 72 hours to select for successfully transduced cells prior to further manipulation.

RNA preparation and quantitative PCR

RNA was extracted and quantitative PCR performed as described (Somerville et al., 2015). Briefly, RNA extraction was performed using the RNeasy Plus Micro kit (for 5×10^5 cells or less) or Mini kit (for greater than 5×10^5 cells) and QIAshredder spin columns. Cells were washed twice in PBS and lysed by vortexing in 350 μL of RLT lysis buffer supplemented with 1% β-mercaptoethanol. Cell lysate was subsequently passed through a QIAshredder spin column for homogenization and homogenized lysates were then passed through a gDNA eliminator spin column to remove genomic DNA contamination. 350 μL of 70% ethanol was added prior to loading the sample onto a MinElute spin column. Following several washes of the column and a 5 minute high speed spin to remove residual ethanol from the column, RNA bound to the column was eluted with RNase-free water. RNA yield was quantified through spectrophotometric analysis using a Nanodrop.

For reverse transcription, between 1 μg and 100ng of extracted RNA from each cell population was diluted in 10 μL of nuclease-free water and with 10 μL of a reverse transcriptase "mastermix" (High Capacity Reverse Transcription kit) according to the manufacturer's instructions. The cDNA generated was diluted with nuclease-free water to an appropriate concentration (typically 10ng/μL).

qPCR reactions were performed in MicroAmp optical 384-well reaction plates and analyzed using QuantStudio Real Time PCR system. Reactions were performed in triplicate and included primers for β-Actin (*ACTB*) as a housekeeping gene. Primers were designed using the Universal Probe Library Assay Design Center and purchased from Integrated DNA Technologies (Coralville, IA). For some assays 20xTaqMan primer/probe assays (Life Technologies) were used, as shown in Table S4. Raw fluorescence data was converted to Ct values using the Thermo Fisher Cloud facility or SDS software v2.1 and normalized to *ACTB*.

KLF2 enhancer deletion

Guides for KLF2 enhancer (chr19:16,328,315-16,328,786) were designed using the CRISPOR tool (http://crispor.tefor.net/). Potential restriction sites were identified in high scoring pairs at alternate ends of the enhancer region. Guides were produced as chemically modified single guide RNA by Synthego. The resulting guides are shown in Table S4. Fujioka cells were cultured in antibiotic free medium to improve electroporation efficiency. Formation of the RNP complex required the reconstitution of sgRNA in TE buffer (Synthego) to a concentration of 50 μM. For the KLF2 enhancer knockout, guides were mixed in a 1:1:1:1 ratio and supplemented with TE buffer to give a total guide concentration of 44 μM in a volume of 0.5 μL per transfection reaction. The non-targeting control scrambled guide was reconstituted to 50 μM and diluted with TE buffer to 0.44 μM in a volume of 0.5 μL per reaction. Alt-R Cas9 was diluted to 36 μM in electroporation buffer R available in the Neon Transfection System kit (ThermoFisher Scientific) to a total volume of 0.5 μL per reaction. The enzyme mix was then incubated with the guide mix in a 1:1 ratio for 20 minutes at room temperature to form the RNP complex.

Each electroporation reaction involved the electroporation of 200,000 cells. To achieve the 2 million cells required for each condition the reaction was performed ten times. For both RNP-KLF2 enhancer KO and RNP-WT 2 million Fujioka cells were centrifuged at 400 g for 5 minutes at 37°C. The pellet was washed in 5ml of PBS to remove any remaining media and spun down again. Cells were resuspended in 10 μL buffer R per reaction, totalling 100 μL per condition. 1 μL of RNP complex per reaction was added to the cell suspension totalling 10 μL, mixed by aspiration and allowed to incubate for 5 minutes.

Electroporation was performed using Neon Transfection System 10 μL tips. For each condition 10 electroporation reactions were performed using a pulse voltage of 1700V and a pulse time of 20 s for a single pulse. Cells were immediately transferred to prewarmed medium following electroporation and returned to incubation at 37°C. 72 hours following electroporation, a sub-fraction of cells was collected for genomic extraction and deletion analysis using the DNeasy Blood and Tissue kit. PCR primers (shown in Table S4) were designed to flank the CRISPR restriction sites.

The remaining cells were infected with viral particles. Five days later cells were collected for RNA extraction, qPCR and flow cytometry.

RNA sequencing

Total RNA was extracted from cells using QIAshredder spin columns and an RNeasy Plus Micro Kit. RNA quality was checked using the Agilent Bioanalyzer. Indexed total RNA libraries were prepared with an input of 500ng of total RNA and 10 cycles of amplification using the TruSeq Stranded Total RNA LT Sample Preparation Kit – Set A (with Ribozero Gold). Library quality was checked using the Agilent Bioanalyzer. Libraries were quantified by qPCR using the KAPA Library Quantification Kit for Illumina. 1.8 pM pooled libraries were loaded onto the NextSeq 500 and 2x75bp sequencing was carried out using a NextSeq 500/550 High Output v2 kit. Reads were aligned to the human genome (GRCh38 and gene annotated with its corresponding GTF files (GENCODE GRCh38) using STAR version 2.4.2a with the settings–outFilterMultimapNmax 20,–outFilterType BySJout,–alignSJoverhangMin 8,–quantMode GeneCounts (Dobin et al., 2013). DESeq2 was used to perform differential gene expression analysis and calculate FPKM (fragments per kilobase of transcript per million mapped reads) values for each gene, counting only reads that mapped to exonic regions (Love et al., 2014).

Chromatin immunoprecipitation and next generation sequencing

Fujioka cells were infected with lentiviral particles targeting *FOXC1* for KD, or a non-targeting control. Next day, cells were drug selected with puromycin 3 μg/mL and incubated for three days. Cells were counted and cross-linked using 1% formaldehyde for 10 minutes (H3K4Me1, H3K4Me2, H3K27Ac, SPI1 and FOXC1), or double cross-linked (for CEBPA, SMARCC2, RUNX1, TLE3, EP300 and HDAC1) with ChIP Cross-link Gold for 30 minutes in PBS with 1mM MgCl2 and then with 1% formaldehyde for 10 minutes. The reaction was stopped by incubation for five minutes with 0.125M glycine. Cell pellets were washed twice with cold PBS containing protease inhibitors (Complete EDTA-free tablets). 10^8 cells were used per ChIP, as per Lee et al. (2006). Briefly, nuclear lysates were sonicated using a Bioruptor Pico for 8 cycles, 30 s ON, 30 s OFF settings. Immunoprecipitation was performed overnight at 20 rpm and 4°C, with 100μl Dynabeads (Protein G) per 10 μg antibody.

After washing six times with RIPA buffer (50mM HEPES pH7.6, 1mM EDTA, 0.7% Na deoxycholate, 1% NP40, 0.5M LiCl), chromatin IP-bound fractions were extracted at 65°C for 30 minutes with elution buffer (50mM TrisHCl pH8, 10mM EDTA, 1% SDS) vortexing frequently. RNaseA (0.2mg/ml) and proteinase K (0.2 μg/ml) were used to eliminate any RNA or protein from the samples. Finally DNA was extracted using phenol:chloroform:isoamyl alcohol extraction and precipitated with ethanol (adding two volumes

of ice-cold 100% ethanol, glycogen (20 μg/μl) and 200mM NaCl) for at least one hour at −80°C. Pellets were washed with 70% ethanol and eluted in 50μl 10mM TrisHCl pH8.0.

ChIP DNA samples were prepared for sequencing using the Microplex Library Preparation Kit and 1ng ChIP DNA. Libraries were size selected with AMPure beads for 200-800 base pair size range and quantified by Q-PCR using a KAPA Library Quantification Kit. ChIP-seq data were generated using the NextSeq platform from Illumina with 2x75bp Hi Output. Reads were aligned to the human genome (GRCh38) using BWA-MEM version 0.7.15 (Li and Durbin, 2009). Reads were filtered using Samtools (version 0.1.9) (Li et al., 2009) (to keep only reads that mapped to standard chromosomes) and Bedtools version 2.25.0 (Quinlan and Hall, 2010) (to remove reads mapped to blacklisted regions defined by ENCODE (http://mitra.stanford.edu/kundaje)). Peaks were called with Model-based Analysis of ChIP-Seq version 2 (MACS2) using the following parameters -f BAMPE,–keep-dup 5 to keep only paired-end reads with up to 5 duplicates (Zhang et al., 2008). Annotation of peaks was performed with Homer version 4.10 (Heinz et al., 2010).

ChIP PCR

For ChIP quantitative PCR, assays were performed in 384-well MicroAmp optical reaction plates using Taqman Fast Universal PCR Mastermix and Universal Probe Library System designed primers and probes. Signal was detected using an ABI PRISM 7900HT Sequence Detection System. Primers and probes used are shown in Table S4.

ATAC sequencing

Fujioka cells were infected with lentiviral particles targeting *FOXC1* for KD, or a non-targeting control. Next day, cells were drug selected with puromycin 3 μg/mL and incubated for three days. Next the Assay for Transposase Accessible Chromatin (ATACseq) protocol (Buenrostro et al., 2013) was performed using 50,000 viable Fujioka cells. Cell pellets were re-suspended in 50 μL lysis buffer (10mM Tris-HCL pH7.4, 10mM NaCl, 3mM MgCl2, 0.1% IGEPAL CA-630) and nuclei were pelleted by centrifugation for 10 minutes at 500 g. Supernatant was discarded and nuclei were suspended in 25 μL reaction buffer containing 2 μL Tn5 transposase and 12.5μl TD buffer (Nextera Sample preparation kit). The reaction was incubated for 30 minutes at 37°C and 300rpm, and purified using the QIAGEN MinElute Kit. Library fragments were amplified using 1x NEB Next High-Fidelity PCR master mix and 1.25 μM of custom PCR primers and conditions (Buenrostro et al., 2013). The PCR reaction was monitored to reduce GC and size bias by amplifying the full libraries for five cycles and taking an aliquot to run for 20 cycles using the same PCR cocktail and 0.6x SYBR Green. The remaining 45μl reaction was amplified for additional cycles as determined by qPCR. Libraries were finally purified using the QIAGEN MinElute Kit. Libraries were size selected with AMPure beads for 200-800 base pair size range and quantified by Q-PCR using a Kapa Library Quantification Kit. ATACseq data were generated using the NextSeq platform from Illumina with a 2x75bp High Output.

Sequencing reads were quality checked using FASTQC (Andrews, 2010). Any adaptor sequences present in the data were removed using Cutadapt (Martin, 2012). The cleaned and trimmed FASTQ files were mapped to the hg38 reference assembly using BWA (Li and Durbin, 2009) and processed using Samtools (Li et al., 2009). The data were cleaned for duplicates, low mapping quality reads (i.e., MAPQ < 30), non-uniquely mapped reads, not properly paired reads and reads mapped to non-conventional chromosomes and mitochondrial DNA.

QUANTIFICATION AND STATISTICAL ANALYSIS

Gene set enrichment analysis

Pre-ranked gene set enrichment analysis was performed with GSEA v2.0.14 software from https://www.broadinstitute.org/gsea (Subramanian et al., 2005). Genes were rank ordered according to log2 fold change in expression (Table S3).

ChIP sequencing normalization between experiments

To normalize ChIP signal between control and *FOXC1* KD Fujioka cells, reads surrounding the absolute summit of 160,041 transcription factor binding peaks were counted. The binding peak sets used were (i) all CEBPA peaks in control Fujioka cells (n = 36,856), (ii) all RUNX1 peaks in control Fujioka cells (n = 34,180), (iii) all SPI1 peaks in control Fujioka cells (n = 34,717), (iv) all FOXC1 peaks in control Fujioka cells (n = 18,745), (v) all RUNX1 peaks in FOXC1 KD Fujioka cells (n = 17,589) and (vi) the coordinates of all MYB binding peaks in THP1 AML cells (n = 17,954) (Maiques-Diaz et al., 2018). For histone modifications reads were counted ± 1,000 base pairs each side of each absolute summit; for all other ChIP-seq experiments reads were counted ± 300 base pairs. For each of the 160,041 value pairs, a fold change in ChIP signal between control and FOXC1 KD conditions was calculated. The list of 160,041 value pairs was then ranked from high to low in Excel based on the number of reads in the control condition. The normalized read count surrounding each peak in the *FOXC1* KD condition was the total KD read count multiplied by the mean of the 2499 subsequent fold change values in the rank ordered list as well as the value for that peak. This "2500 value running mean" approach to normalization is superior to normalization using total mapped reads because it accounts for variations in fold change in ChIP signal according to peak strength and also excludes background reads. Relative increases and decreased in ChIP-seq signal strength were confirmed using ChIP PCR.

Statistics

Statistical analyses were performed using Microsoft Excel 2007 or StatsDirect software (v.1.9.7). Details of the statistical tests used for each analysis shown may be found in the figure legends.

Cell Reports, Volume *36*

Supplemental information

Enhancer recruitment of transcription repressors RUNX1 and TLE3 by mis-expressed FOXC1 blocks differentiation in acute myeloid leukemia

Fabrizio Simeoni, Isabel Romero-Camarero, Francesco Camera, Fabio M.R. Amaral, Oliver J. Sinclair, Evangelia K. Papachristou, Gary J. Spencer, Michael Lie-A-Ling, Georges Lacaud, Daniel H. Wiseman, Jason S. Carroll, and Tim C.P. Somervaille

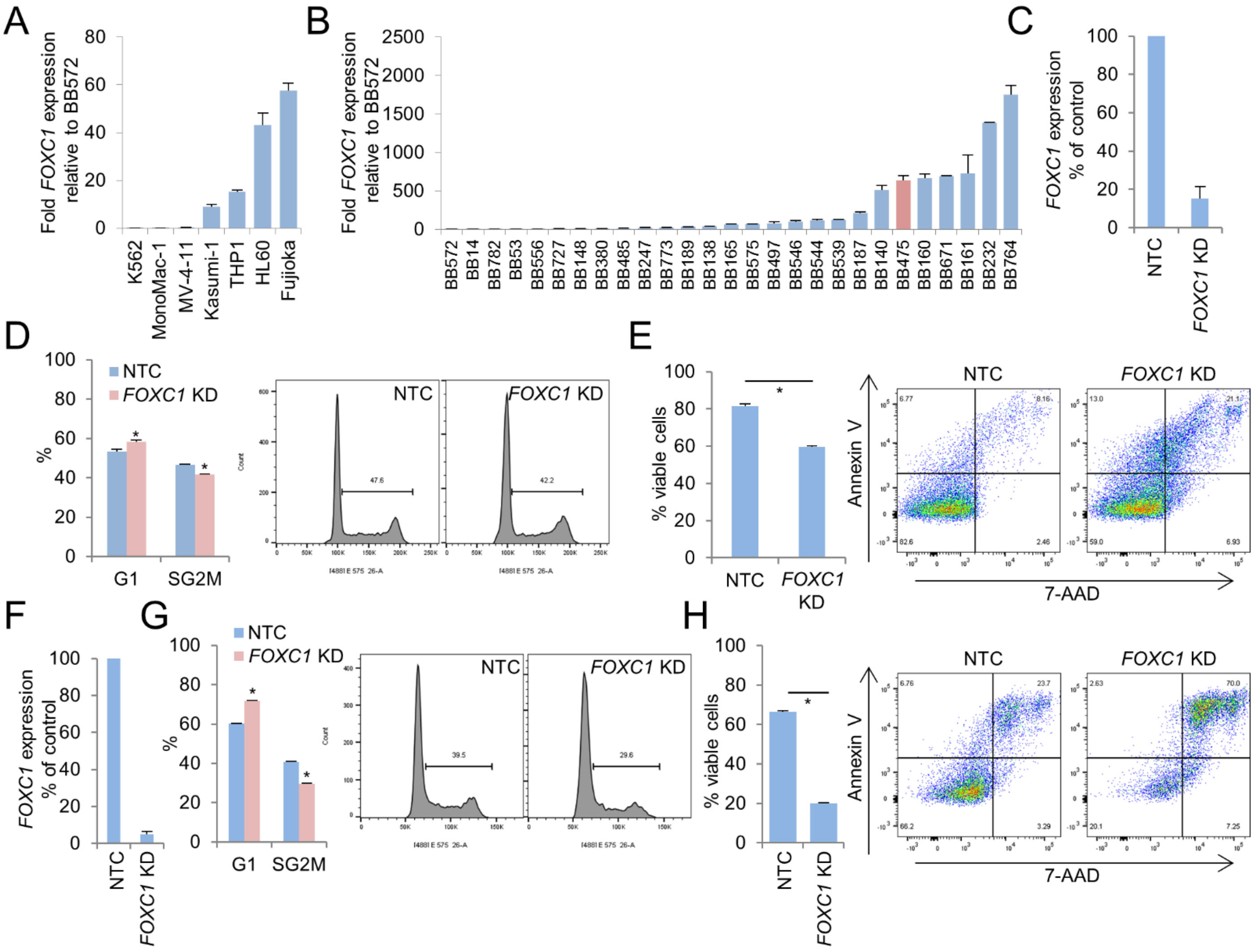

Figure S1. *FOXC1* expression in AML cells. Related to Figure 1.

Bar charts shows mean+SEM relative expression of *FOXC1* in (A) the indicated human AML cell lines and (B) bulk primary human AML samples (n=28), by Q-PCR. (C-E) Human Fujioka AML cells were infected with a lentivirus targeting *FOXC1* for KD or a non-targeting control vector (NTC). (C) Bar chart shows mean+SEM relative expression of *FOXC1* in KD versus control cells (n=3) 72hrs after initiation of KD. (D) Bar chart (left panel) shows mean+SEM percentage of cells in G1 or SG_2M six days following initiation of KD. Right panels: representative cell cycle profiles. (E) Bar chart (left panel) shows mean+SEM percentage of viable cells as determined by Annexin-V/7-AAD analysis seven days following initiation of KD (n=3). Right panels: representative flow cytometry plots. (F-H) Primary patient AML cells (BB475) were infected with a lentivirus targeting *FOXC1* for KD or NTC (n=2). (F) Bar chart shows mean+SEM relative expression of *FOXC1* KD in KD versus control cells (n=2) 72hrs after initiation of KD. (G) Bar chart (left panel) shows mean+SEM percentage of cells in G1 or SG_2M six days following initiation of KD. Right panels: representative cell cycle profiles (n=2). (H) Bar chart (left panel) shows mean+SEM percentage of viable cells as determined by Annexin-V/7-AAD analysis seven days following initiation of KD (n=2). * indicates $P<0.05$ for the indicated comparisons by t-test.

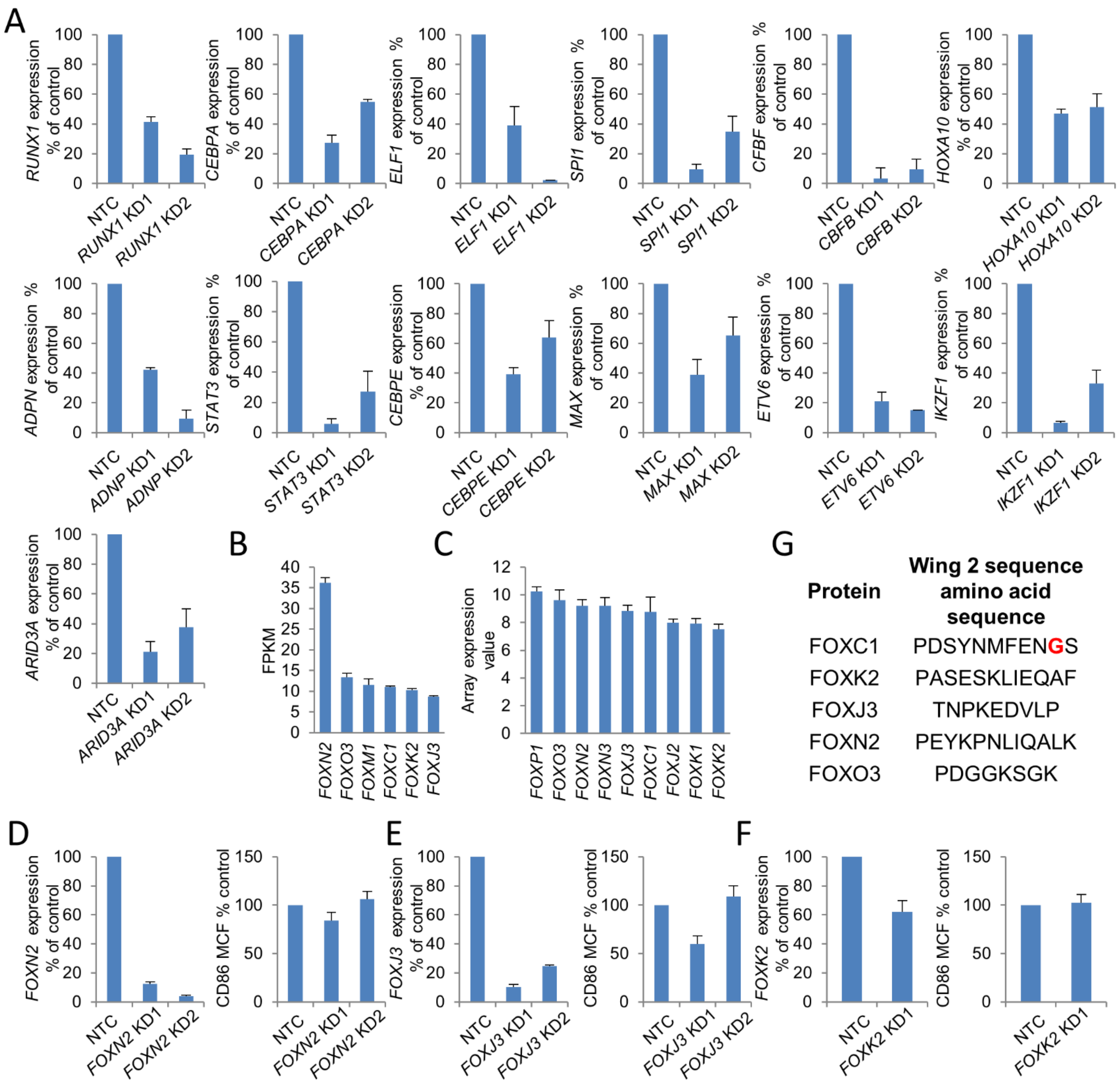

Figure S2. Knockdown of transcription factor genes in Fujioka AML cells. Related to Figure 2.

(A) Graphs show mean+SEM (n=3) relative expression following KD of the indicated genes in human Fujioka AML cells with the indicated lentiviral KD constructs relative to a non-targeting control (NTC). Mean+SEM expression values for all Forkhead family genes where expression values were among the top 33% of all protein coding genes in (B) Fujioka cells (fragments per kilobase per million mapped reads) (n=2) or (C) primary human FOXC1^{high} AML cases (n=100); data extracted from Wouters et al. (2009). (D-F) Graphs (left panels) show mean+SEM (n=3) relative expression following KD of the indicated genes in human Fujioka AML cells with the indicated lentiviral KD constructs relative to a non-targeting control (NTC) and (right panels) mean+SEM CD86 cell fluorescence as determined by flow cytometry analysis on Day 5 (n=3). (G) Amino acid sequences of Wing 2 of the Forkhead domains in the indicated human proteins. G165 of FOXC1 is highlighted in red.

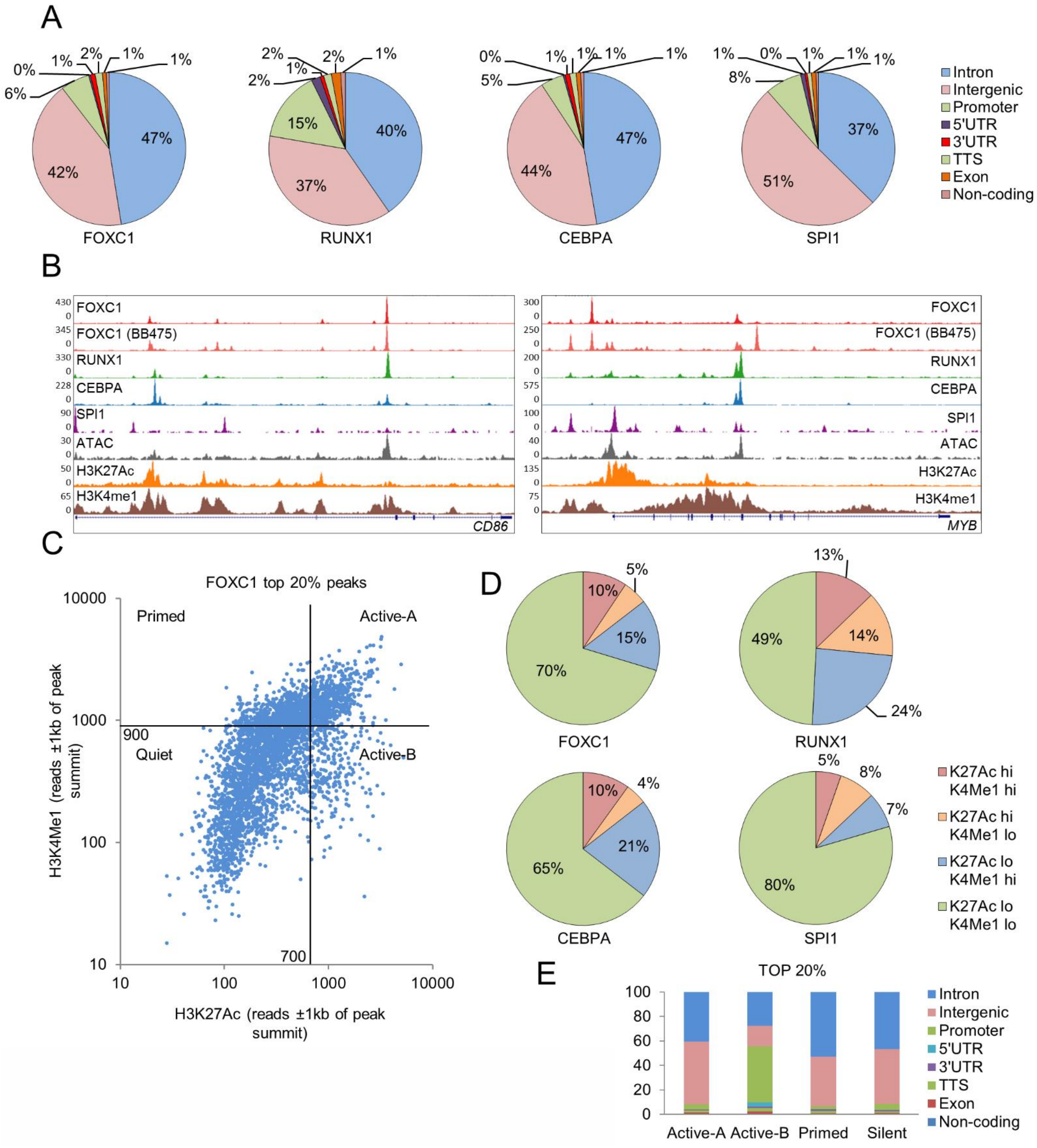

Figure S3. Chromatin context of FOXC1, RUNX1, CEBPA and SPI1 binding peaks. Related to Figure 3.

(A) Pie charts show genome annotations for all transcription factor binding peaks. (B) Exemplar ChIP-seq tracks. (C) Dot plot shows H3K27Ac versus H3K4Me1 reads ±1kB from the absolute summit of each of the strongest 20% of FOXC1 peaks (n=3,773). This facilitated the annotation of transcription factor binding peaks according to their surrounding chromatin into four categories. (D) Pie charts show chromatin categories for all transcription factor binding peaks. (E) Genome region annotations for the strongest 20% of FOXC1 peaks (n=3,773) according to chromatin category shown in (C).

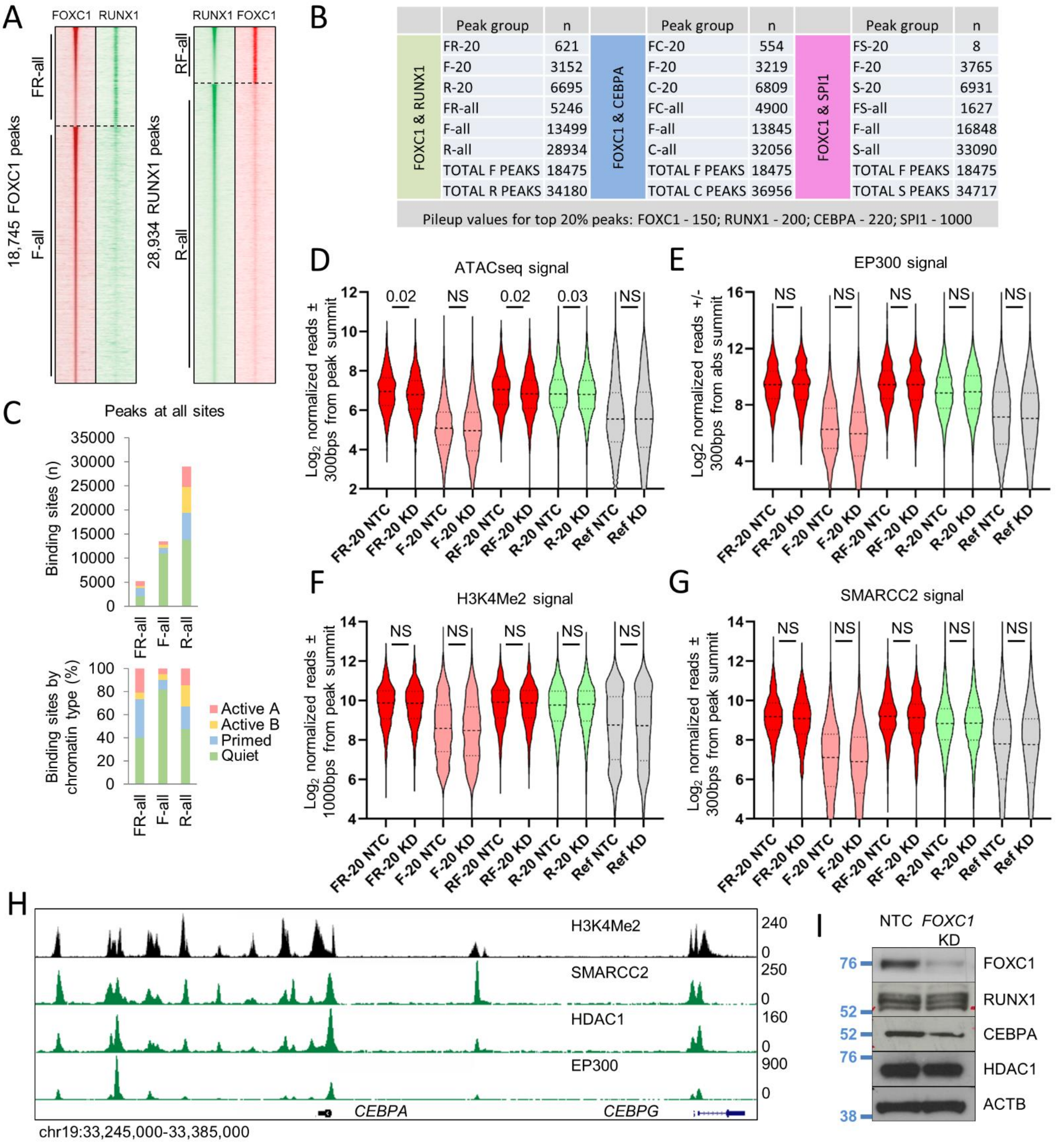

Figure S4. FOXC1 colocalization with transcription and epigenetic factors. Related to Figure 4.

(A) Heatmaps show ChIP signal for RUNX1 at all FOXC1 binding sites (left panel) and FOXC1 at all RUNX1 binding sites (right panel). (B) Table shows the number of binding peaks in each of the indicated categories. (C) Bar charts show chromatin categories for the indicated classes of FOXC1 and RUNX1 binding peaks in Fujioka cells by number (upper panel) and proportion (lower panel). (D-G) Violin plots show distribution, median (thick dotted line) and interquartile range (light dotted lines) for ChIP signal for the indicated proteins at sites with strong FOXC1 and RUNX1 binding (FR-20, FOXC1 centered; RF-20 RUNX1 centered), FOXC1 binding (F-20) or RUNX1 binding (R-20) in Fujioka AML cells in control cells (NTC) or following *FOXC1* KD. Ref, reference cohort used for normalization between experiments; NS, not significant. *P* values, unpaired t-test. (H) Exemplar ChIP-seq tracks. (I) Western blots for the indicated proteins in Fujioka cells on Day 4 following initiation of FOXC1 knockdown.

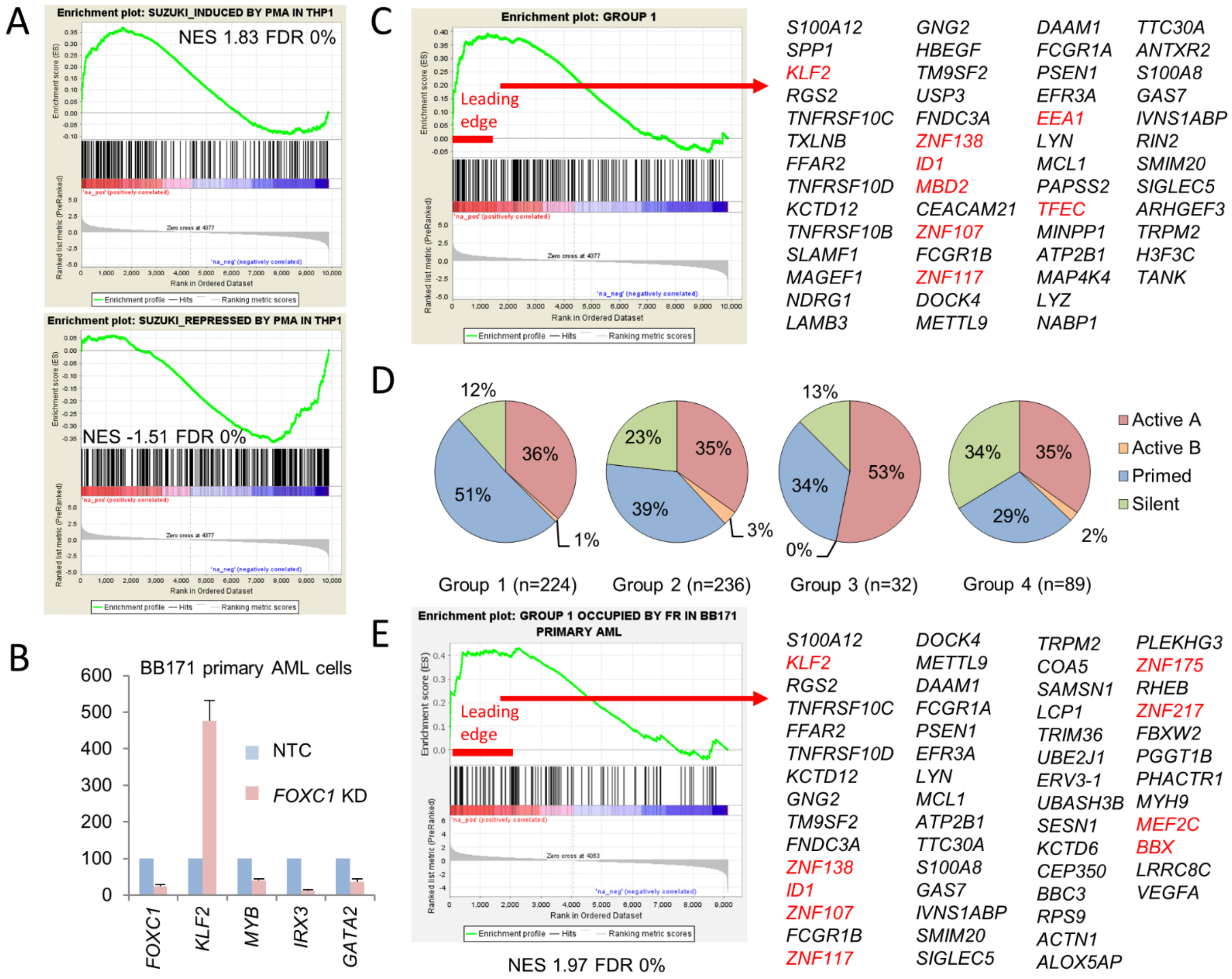

Figure S5. Gene expression and enhancer binding changes after *FOXC1* knockdown. Related to Figure 5.

(A) GSEA plots. NES, normalized enrichment score: FDR, false discovery rate. (B) Bar chart shows mean+SEM relative expression of the indicated genes in BB171 primary AML cells on D5 after initiation of FOXC1 KD. (C) Leading edge analysis of genes located near Group 1 FR-20 enhancers. Genes shown in red code for transcription regulators. (D) Pie charts show chromatin categories of the four FR-20 enhancer groups. (E) GSEA plot and leading edge analysis of Group 1 genes which are also co-bound by FOXC1 and RUNX1 in primary AML cells (BB171).

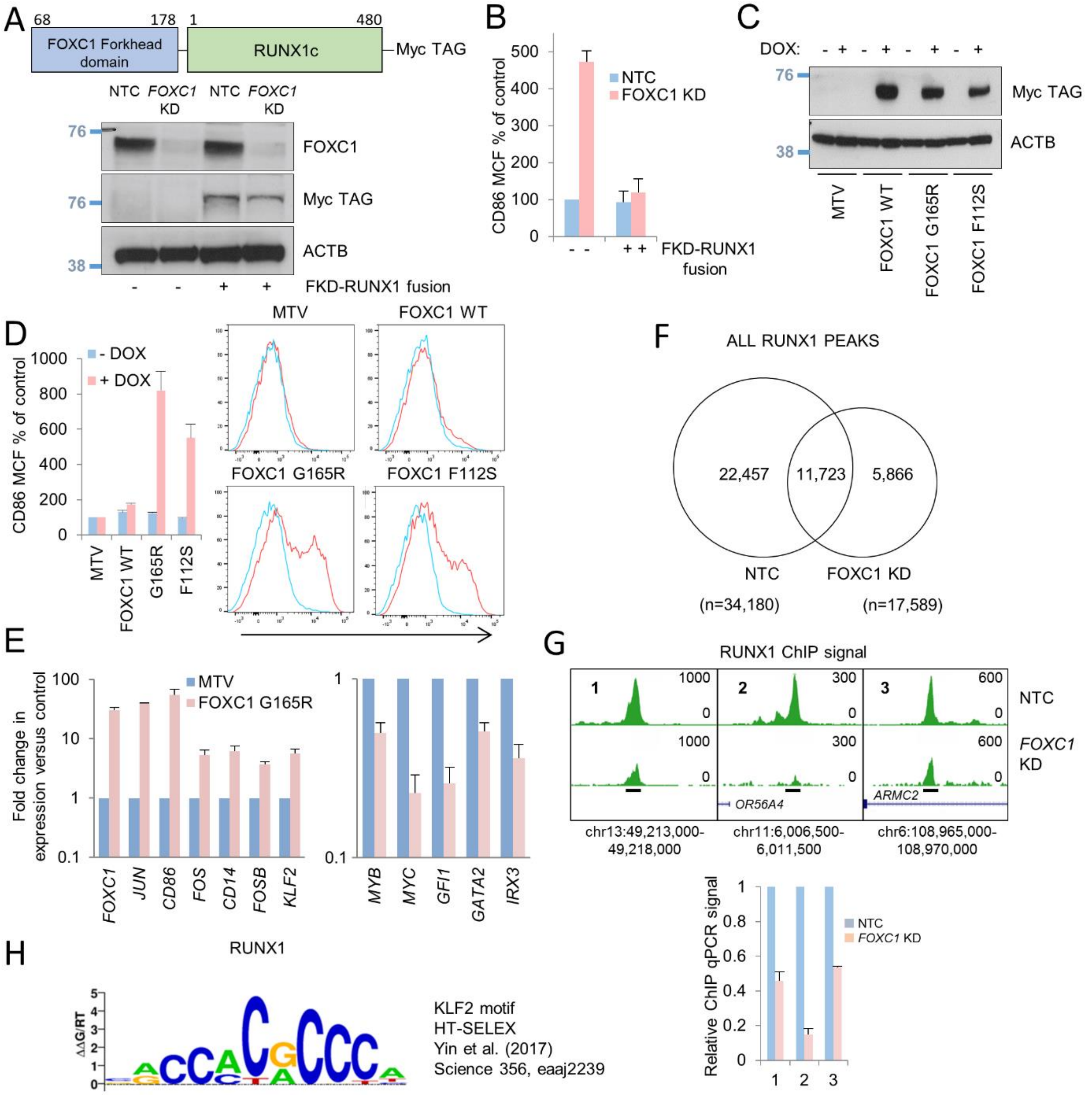

Figure S6. Forced recruitment or displacement of RUNX1 from FOXC1 binding sites regulates expression of differentiation genes; *FOXC1* knockdown triggers redistribution of RUNX1 binding. Related to Figure 6.

(A-B) Fujioka cells were infected with lentiviruses expressing a FOXC1 Forkhead domain–RUNX1c fusion protein under the control of a doxycycline-regulated promoter. (A) Western blots show expression of the indicated protein and transcription factors constructs. (B) Bar chart shows mean+SEM CD86 mean cell fluorescence (MCF) as determined by flow cytometry in the indicated conditions (n=3). (C-E) Fujioka cells were infected with lentiviruses expressing sequences coding for FOXC1 WT, FOXC1 G165R and FOXC1 F112S under the control of a doxycycline-regulated promoter. (C) Western blots show expression of the indicated proteins. (D) Bar chart (left panel) shows mean+SEM CD86 mean cell fluorescence as determined by flow cytometry in the indicated conditions (n=3). Right panel: representative flow cytometry plots. (E) Bar chart shows mean+SEM relative expression of the indicated genes in Fujioka cells of Day 4 following induced expression of FOXC1 G165R. (F) Venn diagram shows intersection of all RUNX1 binding peaks in control (NTC) or *FOXC1* KD Fujioka AML cells. (G) Exemplar ChIPseq tracks at three genomic locations (upper panel) with confirmatory ChIP-PCR for the indicated proteins (lower panel); mean+SEM relative ChIP signal is shown (n=3). (H) KLF2 binding motif.

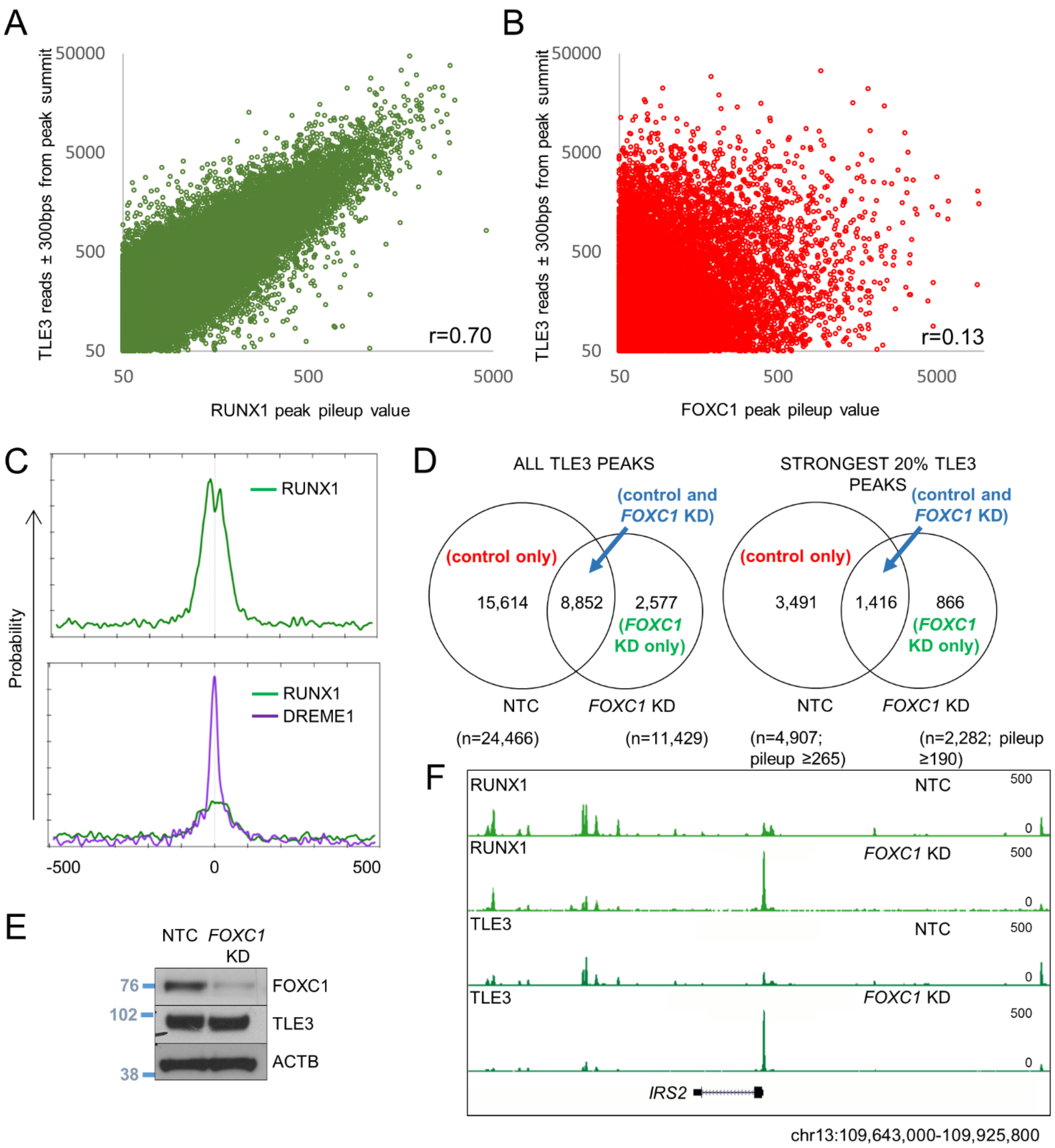

Figure S7. Correlation of RUNX1 and TLE3 ChIP signal and redistribution upon *FOXC1* KD. Related to Figure 7.

Dot plots show TLE3 ChIP signal at (A) RUNX1 and (B) FOXC1 binding peaks in control Fujioka AML cells. (C) MEME-ChIP motif enrichment plots at the strongest 20% of TLE3 binding peaks in control (upper panel) and *FOXC1* KD Fujioka AML cells (lower panel). The DREME1 motif is shown in Figure 7. (D) Venn diagrams show intersection of all (left panel) or the strongest 20% (right panel) of TLE3 binding peaks in control (NTC) or FOXC1 KD Fujioka AML cells. (E) Western blots for the indicated proteins in Fujioka cells on Day 4 following initiation of FOXC1 knockdown (panels for FOXC1 and ACTB are from the same experiment as shown in Figure S4I). (F) Exemplar ChIP-seq tracks.

Table S1. Karyotype & mutations of Fujioka AML cells and primary patient AML samples. Related to Figures 1-7.

Cell line name or biobank number	BM or PB	Karyotype	Identified mutations
Fujioka AML cells		50,X,?add(Y)(q11.2),del(2q)(q35q37),del(4)(q2?8),7,der(7)t(3;7)(q12;q32),inv(9)(p13q12)?c,t(10;11)(p12;q21),+13,+13,+del(13)(q12q14),add(18)(q?21),?+add(18)(q?23),+19	EZH2 p.A736fs, TP53 R196X, ETV6 R399C, NRAS G12C, TP53 Y236C
BB14	PB	46,XY [20]	SFSR2 P95R, ASXL1 G646Wfs*, FLT3 D835E, BCORL1 S953X, CEBPA P189del
BB53	PB	46,XY [20]	FLT3-ITD, BPA P112Sfs, K313dup
BB138	BM	46,XX [20]	NPM1 L287fs, NRAS G12A/G12S/G13D, RAD21 W18X, NOTCH1 V2229
BB140	PB	46,XY [20]	FLT3-ITD, NPM1 L287fs
BB148	PB	47,XY,+11[1]/48,sl,+8[7]/49,sdl,+4[2]	KRAS G12V G13D
BB160	PB	46,XY [20]	IDH2 R140Q, BCOR L884P
BB161	PB	46,XY,t(6;11)(q27;q23)[10]/48,idem,+der(6)t(6;11),+21[4]	SRSF2 P95H, ASXL1 T655Pfs*63, IDH2 R140Q, NMP1 W288Cfs*12, GATA2 G200Vfs*18, CEBPA P14L
BB165	BM	46,XX,t(8;22)(p11;q13),del(9)(q13q32)[10]	TET2 G1754R, DNTM3A R55H
BB171	PB	46,XX [20]	IDH1 R132H EZH2 P432LFS*31
BB187	BM	47,XY,+8[5]/46,XY[5]	SRSF2 P95R, IDH1 R132C, DNMT3A R882H, RUNX1 Y414Ffs*187, PHF6 A288_I290del, BCOR P910L
BB189	PM	46,XX [20]	DNMT3A R882L, NPM1 L287fs, FLT-ITD
BB232	PB	46,XX [20]	ASXL1 T655Pfs*63 (44bp ins), IDH1 R132C, DNMT3A R882H, WT1<br>R302Lfs*3/S313Lfs*70, NOTCH1 A1778V, CELSR2 T1454M, CSMD3 D2372E
BB247	PB	46,XY [20]	DNMT3A S349X, FLT3 D835V, NMP1 W288Cfs*12, GATA2 T354delinsTQ, CDKN2A I27L
BB380	PB	46,XX [20]	TET2 Q913Ffs*11, SH2B3 G451S
BB475	PB	46,XX [20]	IDH2 R140Q, DNMT3A R882H, FLT3-ITD, NPM1 W288C fs*12, RAD21 A544V
BB485	PB	46,XY [20]	IDH1 R132H, DNMT3A S714C, FLT3 D839G, NMP1 W288Cfs*12, KRAS Q61L, PTPN11 D61H
BB497	PM	Failed	DNMT3A R882H, FLT3-ITD, NPM1 W288Cfs*12, WT1 T382Ifs*9
BB539	BM	46,XY [20]	IDH1 R132H, NMP1 W290Efs*10, PTPN11 W290Efs*10
BB544	PM	46,XX [20]	FLT3 D835Y, NMP1 W288Cfs*12
BB546	PB	46,XY [20]	SF3B1 K666N, FLT3-ITD, WT1<br>V368fs, STAG2 K692R
BB556	PB	46,XY [20]	DNMT3A R882C, NMP1 L287fs, NRAS G13D, STAG2 T626fs
BB572	PB	46,XY [20]	TET2 T1091fs / Q1274E, ASLX1 G642fs, RUNX1 R201P / Y287fs / S318fs , KRAS A59E, BCOR E1185fs, ZRSR2 E79fs
BB575	PM	46,XX [20]	SRSF2 M89V, DNMT3A R882H, FLT3-ITD, NPM1 L287fs, SMC3 L242P
BB671	PB	46,XY [20]	DNMT3A R882H, FLT3-ITD, NPM1 W288Cfs*12
BB727	PB	Inv(16)	FLT3-ITD, FLT3 D835Y
BB764	BM	Failed	DNMT3A F755S, FLT3-ITD, FLT3 D835Y, NPM1 W288Cfs*12, PTPN11 E76K
BB773	PB	46,XY [20]	DNMT3A R882H, FLT3-ITD, NPM1 W288Cfs*12,
BB782	PB	46,XX,t(9;11)(p21.3;q23)[10]	No detected mutation
